# Pyrrole-Based Enaminones as Building Blocks for the Synthesis of Indolizines and Pyrrolo[1,2-*a*]pyrazines Showing Potent Antifungal Activity

**DOI:** 10.3390/molecules28207223

**Published:** 2023-10-23

**Authors:** Diter Miranda-Sánchez, Carlos H. Escalante, Dulce Andrade-Pavón, Omar Gómez-García, Edson Barrera, Lourdes Villa-Tanaca, Francisco Delgado, Joaquín Tamariz

**Affiliations:** 1 Departamento de Química Orgánica, Escuela Nacional de Ciencias Biológicas, Instituto Politécnico Nacional, Prolongación de Carpio y Plan de Ayala S/N, Mexico City 11340, Mexico; dmirandas2000@alumno.ipn.mx (D.M.-S.); cescalantep1700@alumno.ipn.mx (C.H.E.); jogomezga@ipn.mx (O.G.-G.); ebarrerac0800@alumno.ipn.mx (E.B.); jdelgador@ipn.mx (F.D.); 2 Departamento de Fisiología, Escuela Nacional de Ciencias Biológicas, Instituto Politécnico Nacional, Av. Wilfrido Massieu S/N, Mexico City 07738, Mexico; dandradep@ipn.mx; 3 Departamento de Microbiología, Escuela Nacional de Ciencias Biológicas, Instituto Politécnico Nacional, Prolongación de Carpio y Plan de Ayala S/N, Mexico City 11340, Mexico; mvillat@ipn.mx

**Keywords:** pyrrolo[1,2-*a*]pyrazines, substituted pyrroles, enaminones, indolizines, antifungal activity, multidrug-resistant *Candida* spp., HMGR inhibition

## Abstract

As a new approach, pyrrolo[1,2-*a*]pyrazines were synthesized through the cyclization of 2-formylpyrrole-based enaminones in the presence of ammonium acetate. The enaminones were prepared with a straightforward method, reacting the corresponding alkyl 2-(2-formyl-1*H*-pyrrol-1-yl)acetates, 2-(2-formyl-1*H*-pyrrol-1-yl)acetonitrile, and 2-(2-formyl-1*H*-pyrrol-1-yl)acetophenones with DMFDMA. Analogous enaminones elaborated from alkyl (*E*)-3-(1*H*-pyrrol-2-yl)acrylates were treated with a Lewis acid to afford indolizines. The antifungal activity of the series of substituted pyrroles, pyrrole-based enaminones, pyrrolo[1,2-*a*]pyrazines, and indolizines was evaluated on six *Candida* spp., including two multidrug-resistant ones. Compared to the reference drugs, most test compounds produced a more robust antifungal effect. Docking analysis suggests that the inhibition of yeast growth was probably mediated by the interaction of the compounds with the catalytic site of HMGR of the *Candida* species.

## 1. Introduction

Pyrrolo[1,2-*a*]pyrazines and indolizines, including their partial or complete saturated analogues, belong to the diverse family of pyrrole-fused aza-bridged heterocyclic compounds, which are abundant in nature. They are known for their strong pharmacological activity. Pyrrolo[1,2-*a*]pyrazines and structural analogues show a wide range of biological effects [1,2], as illustrated by reports on their anxiolytic [3], anticancer [4,5], and antifungal activity [1,6], as well as their use as insect feeding deterrents [7] and potent and selective non-competitive antagonists of mGluR5 [8]. Antibiotic activity has only been found for the hexahydropyrrolo[1,2-*a*]pyrazine-1,4-dione analogues, which provides a strong effect against gram-negative bacteria [9,10,11]. Indolizines, on the other hand, have potential as anticancer, herbicidal, anti-inflammatory, antiparasitic, antioxidant, antihistaminic, anticonvulsant, antiviral, and analgesic agents [12,13,14,15,16,17]. In contrast to pyrrolo[1,2-*a*]pyrazines, antibiotic activity is more widely distributed among indolizines [12,13], in particular against *Mycobacterium tuberculosis* [18,19]. However, the antifungal effect of indolizines is modest [12]. Although the mechanism of action is not clearly established for either heterocycle, it is probably associated with the nitrogen-bridged heterocyclic scaffold [1,12].

**Scheme 1:**
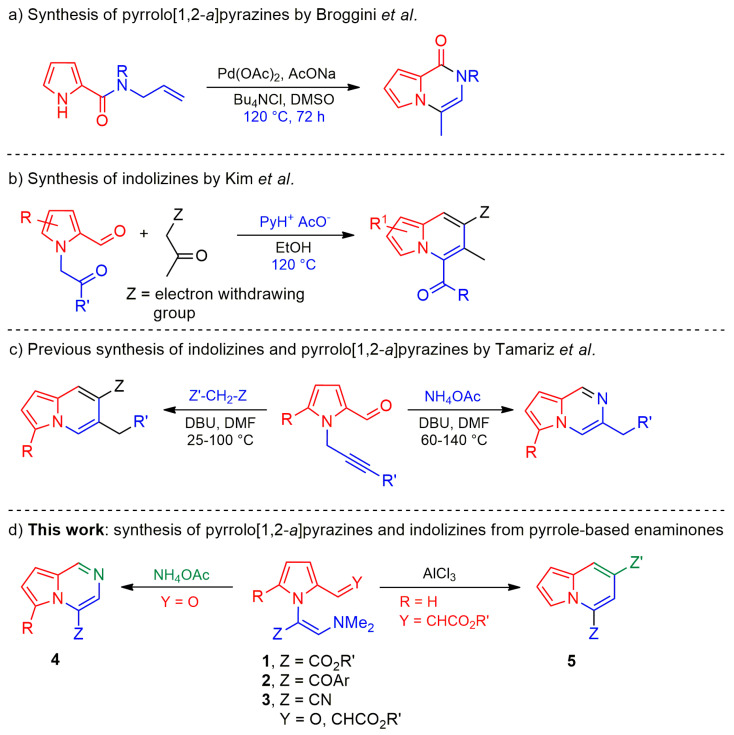
Previously reported synthetic procedures: (**a**) a Pd(II)-promoted vinyl cyclization to achieve pyrrolo[1,2-*a*]pyrazines; (**b**) a condensation process to afford indolizines; and (**c**) the use of 2-formylpyrroles as the starting material to obtain pyrrolo[1,2-*a*]pyrazines and indolizines. (**d**) With the current strategy, pyrrole-based enaminones (**1**–**3**) were the building block for the preparation of pyrrolo[1,2-*a*]pyrazines (**4**) and indolizines (**5**) [23,25,30].

Although pyrrolo[1,2-*a*]pyrazines have received less attention from researchers than indolizines, their broad spectrum of activity has stimulated the discovery of versatile synthetic strategies [1,2]. One of the most efficient approaches involves pyrrole-containing substrates, which undergo a cyclization process to build the pyrazine ring [20,21,22,23,24] (Scheme 1a). Indolizines have been prepared by following an analogous methodology, starting from functionalized pyrroles as the building block and completing the synthesis by a proper annulation to build the six-membered ring [25,26,27,28,29] (Scheme 1b). Our group has recently described effective procedures for constructing the scaffold of both indolizines and pyrrolo[1,2-*a*]pyrazines by appropriately substituting pyrrole-based precursors [30] (Scheme 1c).

The capacity of indolizines [31] and pyrrolo[1,2-*a*]pyrazines [1,6] to treat drug-resistant fungal infections is particularly attractive. Due to the pharmacological value of these heterocycles, the development of short and highly efficient synthetic methods should be a priority.

The aim of the current contribution was to design and test new synthetic approaches for synthesizing pyrrolo[1,2-*a*]pyrazines and indolizines. For the elaboration of pyrrolo[1,2-*a*]pyrazines, the potential of the annulation of 2-formylpyrrole-based enaminones (**1**–**3**) in the presence of ammonium acetate was evaluated. On the other hand, analogous enaminones were exposed to Lewis acids to prepare indolizines (Scheme 1d). All precursors and the corresponding products were assessed for their in vitro antifungal effect on six strains of *Candida* species. The most active compounds were analyzed in silico by docking simulations to examine their pharmacological profile as antifungal agents at the active site of the 3-hydroxy-methyl-glutaryl-CoA reductase (HMGR) enzyme of the six *Candida* spp. HMGR is the rate-limiting enzyme for the formation of ergosterol in *Candida* species, which is generated through the mevalonate pathway. Ergosterol is an essential component in the yeast cell membrane.

## 2. Results and Discussion

### 2.1. Chemistry

The design of pyrrole-containing compounds as potential antifungal agents was based on the evidence that benzofuran [32,33], benzothiophene [34], and indole [35,36] frames (including those of several natural products) can be built through intramolecular cyclization of 2-phenoxy-, 2-thiophenoxy-, and 2-anilino-3-(dimethylamino)propenoates, respectively. The latter derivatives have generically been called enaminones and proven to be versatile substrates in heterocyclic chemistry [37,38].

In the current investigation, the nitrogen atom of the 2-formylpyrrole ring was incorporated into the C-2 position of the alkyl (*Z*)-3-(dimethylamino)propenoate-equivalent (**1**, Y = O, Z = CO_2_R, Scheme 1d), the (*Z*)-1-aryl-3-(dimethylamino)prop-2-en-1-one-equivalent (**2**, Y = O, Z = COAr, Scheme 1d), and the (*Z*)-3-(dimethylamino)acrylonitrile-equivalent (**3**, Y = O, Z = CN) (Scheme 1d), followed by the cyclization process in the presence of ammonium acetate to provide the corresponding pyrrolo[1,2-*a*]pyrazines **4**. It was assumed that 2-formylpyrrole (**6**) can serve as a building block for the divergent synthesis of substituted pyrrolizines [30,39] and pyrrolo[1,2-*a*]pyrazines [30] to furnish the desired pyrrole-fused aza-bridged heterocyclic derivatives.

**Scheme 2:**
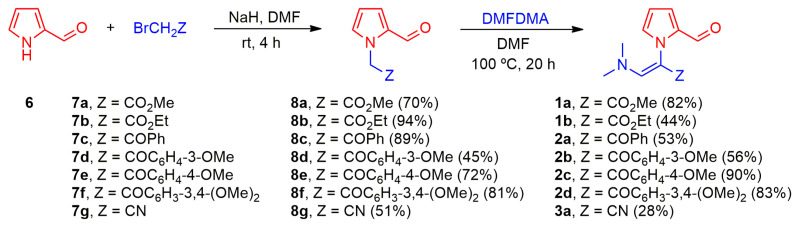
Preparation of 2-formylpyrrole-based enaminones **1a**–**b**, **2a**–**d**, and **3a**.

Thus, the alkylation of **6** with alkyl bromoacetates **7a** and **7b** under basic conditions produced the series of N-substituted pyrroles **8a** [40] and **8b** in good yields (Scheme 2). Similarly, the reaction of **6** with the series of bromoacetophenones **7c**–**f** generated **8c**–**f**, and the reaction of **6** with bromoacetonitrile (**7g**) gave pyrrole **8g**. The thermal treatment of **8a**–**g** with *N,N*-dimethylformamide dimethyl acetal (DMFDMA) afforded the series of enaminones **1a**–**b** and **2a**–**d** in acceptable yields as a single *Z* isomer, as well as enaminone **3a** as an inseparable mixture of *Z*/*E* (54:46) isomers (Scheme 2).

For the conversion of 2-formylpyrrole-based enaminones **1a**–**b**, **2a**–**d**, and **3a** into the series of 4-substituted pyrrolo[1,2-*a*]pyrazines **4a**–**g**, a combination of ammonium acetate (as the source of nitrogen) and DBU (as the base) was employed as previously described [30]. However, the yields were low. Similar low yields were obtained with sodium hydride, potassium *tert*-butoxide, or cesium carbonate as the base. The reaction was improved when applying lithium carbonate as the base and DMF as the solvent at variable temperatures and reaction times (Table 1).

**Scheme 3:**
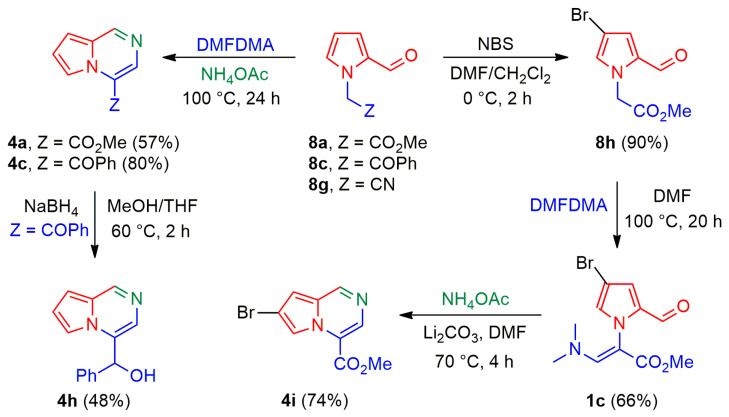
Preparation of pyrrolo[1,2-*a*]pyrazines **4a** and **4c** via a multicomponent procedure, and of **4h** and **4i** by the transformation of **4c** and **8h**, respectively.

As an alternative route, a multicomponent reaction was explored for the preparation of pyrrolo[1,2-*a*]pyrazines **4**, starting from substituted pyrroles **8a**, **8c**, and **8g** in the presence of DMFDMA and ammonium acetate (Scheme 3). Thus, pyrroles **8a** and **8c** were reacted with an excess of DMFDMA (5.0 mol eq.) and ammonium acetate (3.0 mol eq.) to provide the corresponding pyrrolo[1,2-*a*]pyrazines **4a** and **4c** in 57% and 80% yields, respectively. When comparing this one-step process to that of the previously described two-step procedure, the current overall yield was lower for **4a** (57% vs. 74%) and higher for **4c** (80% vs. 46%). The reduction of **4c** with NaBH_4_ resulted in alcohol **4h** in moderate yield. The reaction of **8g** with DMFDMA generated a complex mixture of products that included the starting material.

Considering the important biological properties of brominated pyrroles [40,41] and pyrrolo[1,2-*a*]pyrazines [5], the brominated derivatives **8h** [40], **1c**, and **4i** were conceived as potential antifungal compounds. The bromination of **8a** with NBS (1.0 mol eq.) afforded **8h** as a single regioisomer in high yield (Scheme 3). Treatment of the latter with DMFDMA gave enaminone **1c**, which was reacted with ammonium acetate to deliver 7-bromopyrrolo[1,2-*a*]pyrazine **4i** in good yield.

**Scheme 4:**
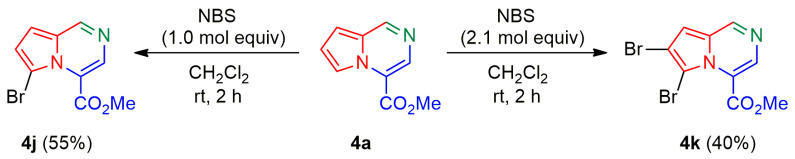
Preparation of pyrrolo[1,2-a]pyrazines **4j** and **4k** by bromination of **4a**.

In the same context, pyrrolo[1,2-*a*]pyrazine **4a** was brominated with an equimolar quantity of NBS to yield 6-bromopyrrolo[1,2-*a*]pyrazine **4j** (Scheme 4), and the reaction of **4a** with two mol equivalents of NBS furnished 6,7-dibromopyrrolo[1,2-*a*]pyrazine **4k**.

**Scheme 5:**
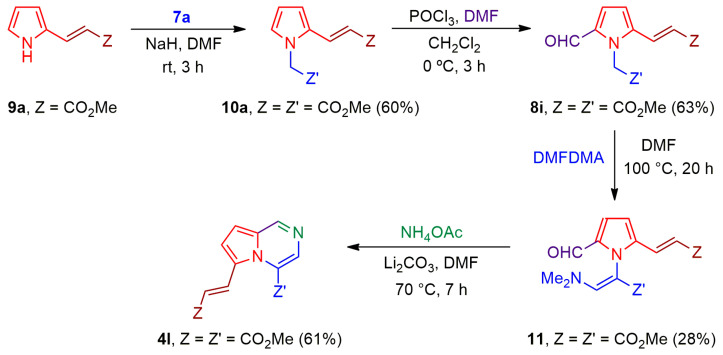
Preparation of pyrrolo[1,2-a]pyrazine **4l** by transformation of pyrrole **9a**.

With the aim of expanding the scope of the methodology, the synthesis of 4,6-disubstituted pyrrolo[1,2-*a*]pyrazine **4l** was undertaken. Starting from **6**, alkyl 3-(pyrrol-2-yl)acrylate **9a** was obtained by a Horner–Wadsworth–Emmons reaction, followed by N-alkylation with **7a** to give pyrrole **10a**, as reported [42] (Scheme 5). The latter was formylated under the usual Vilsmeier–Haack conditions to give rise to the respective 5-formyl analog **8i**. Treatment of **8i** with DMFDMA generated a complex mixture of products, from which it was possible to isolate the single Z isomer of enaminone **11** in low yield. Finally, the amination of the latter with ammonium acetate delivered the desired pyrrolo[1,2-*a*]pyrazine **4l** in modest yield, in contrast to the high yields for **4a**–**g** (Table 1).

**Scheme 6:**
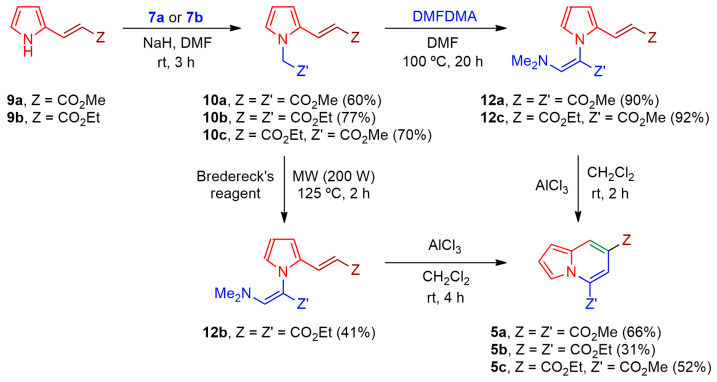
Conversion of the series **10a**–**c** into indolizines **5a**–**c**.

It was decided to start with similar enaminones to test a new approach for the construction of indolizines. Therefore, **9a** or **9b** were reacted with the corresponding methyl or ethyl bromoacetates **7a** and **7b** under basic conditions to afford pyrroles **10a**–**c** [42], which were reacted with DMFDMA to convert them into enaminones **12a**–**c** (Scheme 6). Unfortunately, enaminone **12b** was formed in a mixture of methyl esters as a result of transesterification with the methoxy ions in the reaction medium. Hence, this compound was more efficiently obtained in modest yield when using Bredereck’s reagent under MW irradiation.

With the aim of achieving the direct annulation of **12a**–**c** to the corresponding indolizines **5a**–**c**, the reaction was promoted by diverse Lewis acids. By treating **12a** with ZnI_2_ or CuCl in methylene chloride as the solvent at 0 °C, the starting material was recovered. An increase in the temperature led to the decomposition of the reaction mixture. When utilizing POCl_3_ in DMF at 0 °C, the yield of **5a** was 28%, and the conversion of **12b** to **5b** with this Lewis acid was insignificant. The transformation of **12c** to **5c** with iodine [36] provided a low yield (18%). Finally, a 1.0 M nitrobenzene solution of AlCl_3_ in methylene chloride as the solvent at room temperature efficiently furnished the series **5a**–**c** in variable yields (Scheme 6). Disappointingly, when this strategy was started by reacting pyrrole **9a** with bromoacetophenones **7c**–**f**, the conversion into the corresponding *N*-alkyl pyrroles did not proceed efficiently. The structure of each product obtained in all the synthetic schemes was fully established by ^1^H and ^13^C NMR, assisted by 2D experiments (ROESY, HSQC, and HMBC) and HRMS.

### 2.2. Antifungal Activity

According to previous studies, brominated and non-brominated pyrrole-containing compounds structurally related to derivatives **8a**, **8h**, and **1c** inhibit yeast growth and the synthesis of ergosterol by *Candida glabrata* [40]. Therefore, selected compounds of the present series, consisting of pyrrole-based compounds (**8a**–**i** and **10a**–**c**), enaminones (**1a**–**c**, **2a**–**d**, **11**, and **12a**–**c**), pyrrolo[1,2-*a*]pyrazines (**4a**–**l**), and indolizines (**5a**–**c**), were assessed at their minimum inhibitory concentration (MIC) against six *Candida* spp.: *C. albicans*, *C. glabrata*, *C. dubliniensis*, *C. krusei*, *C. auris*, and *C. haemulonii*. The latter two species are multidrug-resistant. Most of the compounds evaluated afforded substantial yeast growth inhibition. Table 2 summarizes the lowest MIC_50–70_ values in regard to the inhibition of the *Candida* spp. by the test compounds and reference drugs (fluconazole, simvastatin, and atorvastatin). Simvastatin and atorvastatin are well-known inhibitors of human HMGR (hHMGR) and are proposed as inhibitors of HMGR of *Candida* spp. [40,43,44]. Fluconazole is a reference inhibitor of the enzyme C-14 alpha demethylase of lanosterol (CYP51) that participates in the synthesis of ergosterol [45]. Interestingly, simvastatin and atorvastatin showed a more efficient profile than fluconazole for some of the yeasts, including the two multidrug-resistant species.

Inhibition of yeast growth was observed for all the test compounds, which had lower MICs than the three reference drugs in all cases. Nevertheless, the majority of the MIC_50–70_ values for the compounds on *C. auris* and *C. haemulonii* were higher than those obtained on *C. albicans*, *C. glabrata*, *C. dubliniensis*, and *C. krusei*. This can perhaps be explained by the strong resistance of *C. auris* and *C. haemulonii* to a variety of traditional antifungal drugs [46,47].

Pyrrole derivatives are reported to inhibit the growth of *C. albicans*, *C. glabrata*, *C. parapsilosis*, *C. tropicalis*, *Cryptococcus neoformans*, *Saccharomyces cerevisiae*, *Aspergillus fumigatus*, *A. niger*, *Geotrichum candidum*, *Syncephalastrum racemosum*, and dermatophytes (*Trichophyton rubrum*, *T. mentagrophytes*, and *Microsporum gypseum)*, exhibiting in almost all the cases MICs higher than those determined presently [48,49,50,51,52]. These results suggest that the N-alkyl group attached to the pyrrole ring of derivatives **8a**–**g** improves the antifungal effect (especially for N-alkyl 2-formylpyrroles **8a**, **8c,** and **8g**), as seems to be demonstrated for the pyrrole-based drug atorvastatin. Low to modest antifungal activity has also been observed for pyrrole- and/or pyridine-containing fused-bis-heterocyclic compounds [53,54,55].

The few reports on enaminone-containing compounds have evidenced their antibacterial activity against *Escherichia coli*, *Bacillus subtilis*, and *Salmonella typhi* [56] as well as antifungal activity against *C. albicans*, in each case showing a modest inhibition [57]. In the current contribution, the enaminones **1a**, **1c**, **2a**–**d**, **11**, and **12a**–**c** exhibited good inhibition of the six *Candida* spp. (Table 2).

Pyrrole-based compounds **8i** and **10**–**12** bear the acrylate moiety as another potentially active pharmacophore group, as does pyrrolo[1,2-*a*]pyrazine **4l**, one of the most active derivatives against the six *Candida* species. Some acrylate-based heterocyclic derivatives have been proposed as antifungals due to their growth inhibition of *Aspergillus*, *Geotrichum*, and various other pathogenic plant fungi [58,59].

Pyrrolo[1,2-*a*]pyrazines **4a**–**l** were highly active agents against all the *Candida* spp. herein tested, particularly derivatives **4b**, **4g**, and **4l**. The conjugated C-4 carbonyl group does not seem to be a key pharmacophore, given that the antifungal activity is comparable for alcohol **4h** and precursor **4c**. Moderate activity was previously found for analogous heterocycles against *Penicillium chrysogenum* AUMC 530-15, four species of *Aspergillus*, *Fusarium solani* AUMC 2690-6, and *Trichothecium roseum* AUMC 7410-2 [60]. Interestingly, the brominated pyrrolo[1,2-*a*]pyrazines **4i**–**k** were more active than their corresponding non-halogenated **4a**.

The indolizines **5a**–**c** also significantly inhibited the growth of the majority of the *Candida* spp., in agreement with recent reports on other analogous compounds [61]. However, these results are in contrast with a series of 7-cyano-1,2-diphenylindolizines, which were not active on *C. krusei* [18].

### 2.3. Molecular Docking Study

Molecular docking is a computational tool used to explore the interaction, binding mode (e.g., protein–protein), and affinity between two molecules, generally an organic compound and an endogenous receptor (protein or nucleic acid) of a cell [62,63,64]. Because docking simulations serve to explore the molecular recognition between the active site of a certain therapeutic target (an enzyme) and an organic molecule (ligand), they are widely used to design and develop new drugs that may improve the treatment for diseases or for infections by microorganisms, such as a pathogenic fungus. The binding interaction between two molecules depends on the hydrophilic, electrostatic, and hydrophobic intermolecular forces that take place. The strength of such interactions is expressed in binding energy (ΔG, kcal/mol). A more negative value of ΔG is related to a more stable enzyme–ligand complex and a greater biological activity.

To explore the binding interactions of the test compounds on HMGR, the following compounds were selected among the most active derivatives: enaminone-containing pyrroles **1a**, **2a**, and **2c**, pyrrolo[1,2-*a*]pyrazines **4b**, **4g**, and **4l**, indolizine **5a**, *N*-alkyl 2-formylpyrroles **8a**, **8c**, and **8g**, and pyrrole-based alkyl acrylates **10a** and **12a**. The molecular docking study was carried out on the active site of the HMGR enzyme of *C. albicans* (CaHMGR), *C. glabrata* (CgHMGR), *C. dubliniensis* (CdHMGR), *C. krusei* (CkHMGR), *C. auris* (CauHMGR), and *C. haemulonii* (ChaHMGR). The models of the six HMGRs were built (Figure S77, Supplementary Materials) and overlapped with human HMGR, noting a close structural identity between the 3D structures. The high quality of the models employed in the study is evidenced by the fact that over 90% of the amino acid residues were in permitted areas of the Ramachandran diagram (Figures S78–S83, Supplementary Materials), as previously reported for CgHMGR [40,44,65].

The binding energy values of the reference compound atorvastatin were in all cases lower than those for simvastatin (Table 3). A similar pattern of binding energy values was observed for the entire series of compounds. For example, enaminones **2a** and **2c** and the *N*-alkyl pyrrole **8c** showed more negative values than simvastatin for all the species of *Candida* spp. herein tested. The pyrrolo[1,2-*a*]pyrazine **4l** had better binding energy values than simvastatin on five of the yeasts. In contrast to the experimental antifungal effect, enaminone **1a** only exhibited better binding energy values than simvastatin on CaHMGR and CkHMGR. Overall, the binding energy values found for these series of compounds were better than those previously described for other derivatives proposed as inhibitors of CgHMGR [40,44].

**Figure 1:**
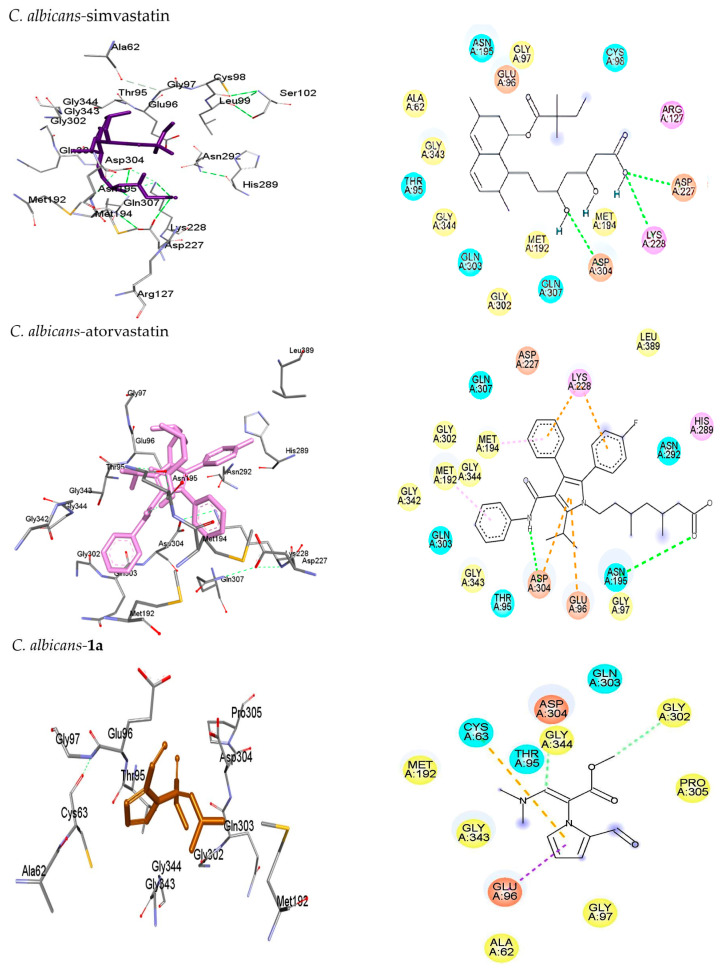

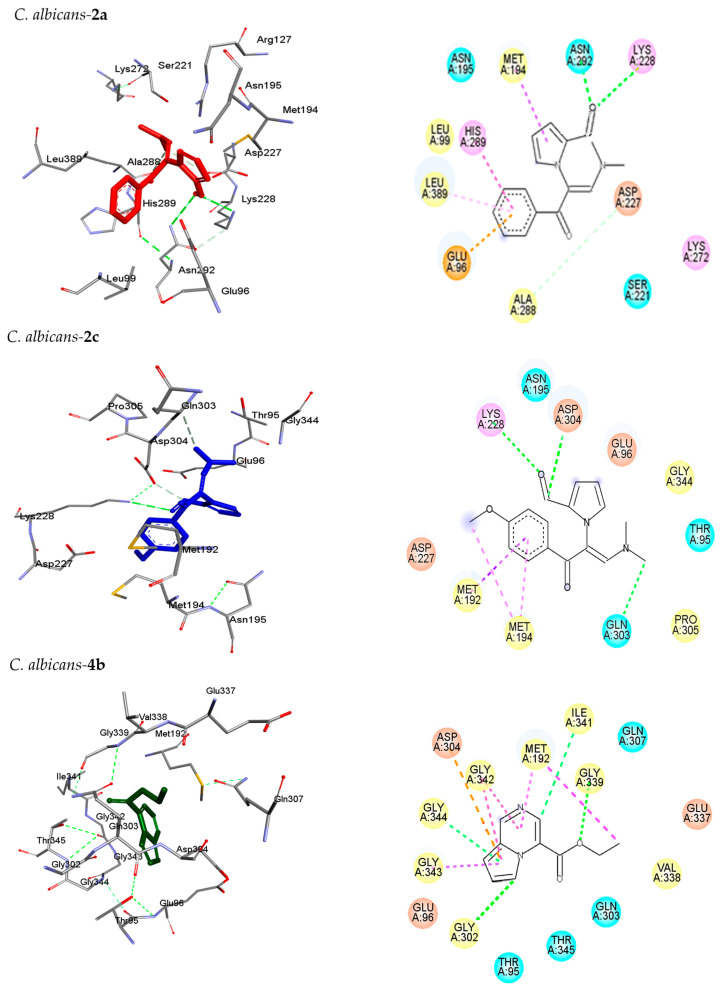

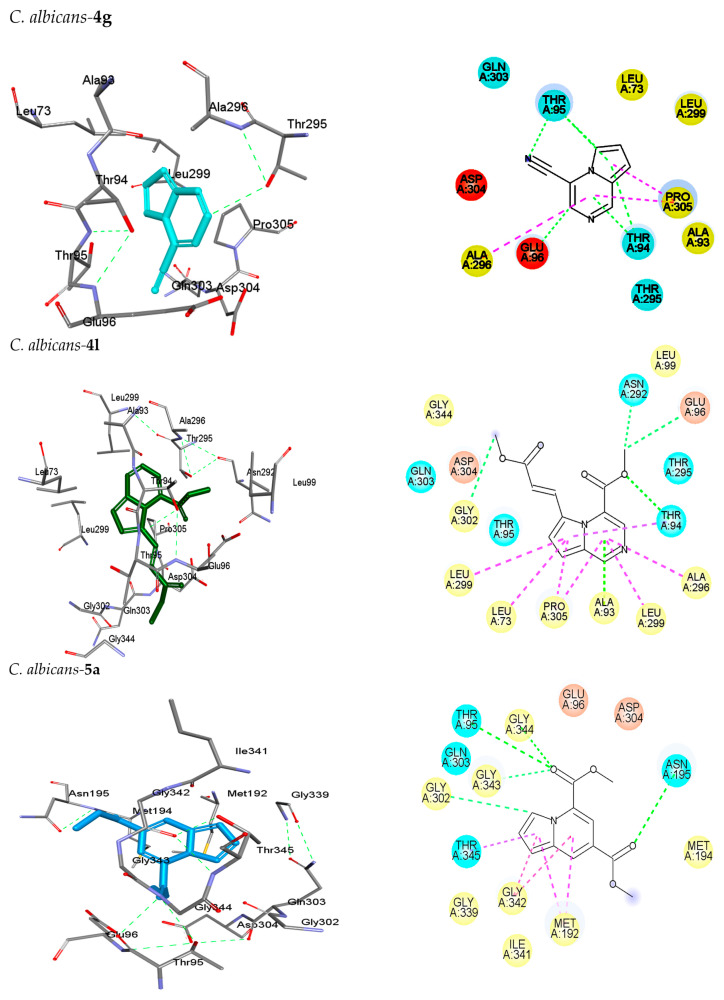

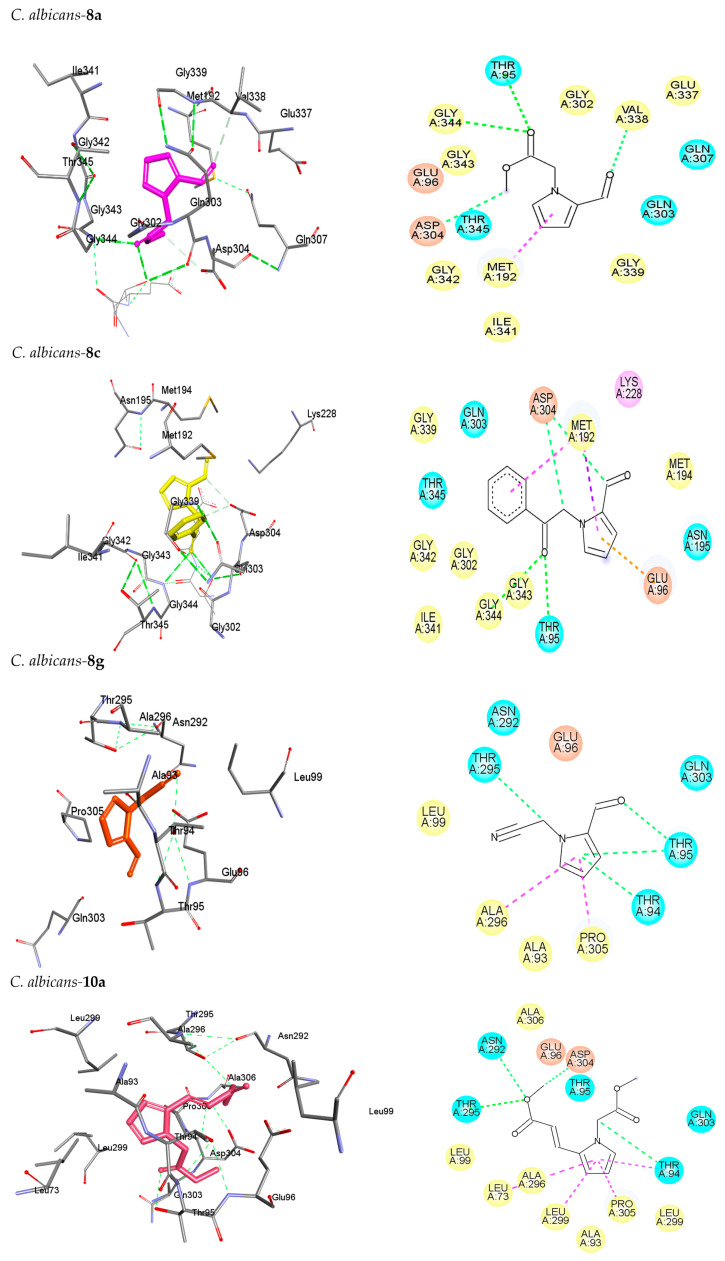

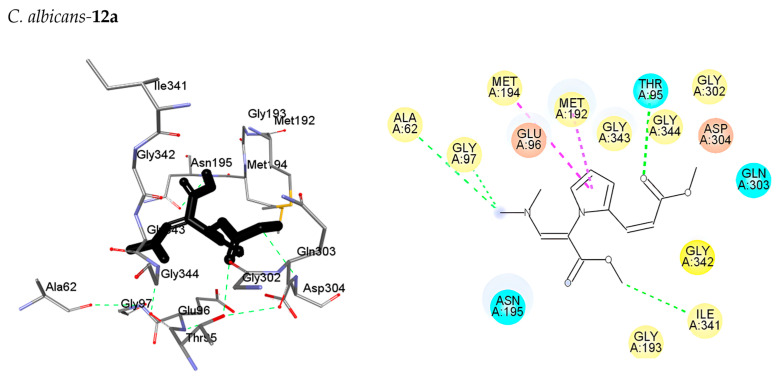
Representation of the interactions between the active site of HMGR of *C. albicans* and simvastatin, atorvastatin, and enaminone-containing pyrroles **1a**, **2a**, and **2c**, pyrrolo[1,2-*a*]pyrazines **4b**, **4g**, and **4l**, indolizine **5a**, *N*-alkyl 2-formylpyrroles **8a**, **8c**, and **8g**, and pyrrole-based alkyl acrylates **10a** and **12a**. The 3D model shows the amino acid residues involved in ligand binding to the active site of the enzyme. In the 2D model, the following interactions are depicted with dotted lines: conventional hydrogen bond (dark green), carbon hydrogen (light green), π–sigma (purple), π–π T-shaped and π–π stacked (fuchsia), π–alkyl and alkyl (pink), π–anion (orange), and π–cation (yellow). The amino acids are represented in pink circles (for basic amino acids), orange (for acid), cyan (for polars), and yellow (for non-polar).

To predict the binding mode of the twelve selected compounds at the active site of the HMGRs, the three most prevalent species (*C. albicans*, *C. glabrata*, and *C. auris*) were selected. The hydrophilic and hydrophobic interactions and the amino acid residues involved are summarized in Table 4 for *C. albicans*, and in Tables S1 and S2 for *C. glabrata*, and *C. auris*, respectively. Likewise, the interactions are illustrated in Figure 1 for *C. albicans* and in Figures S84 and S85 for *C. glabrata* and *C. auris*. Regarding their binding modes to the HMGRs of the three *Candida* spp., the twelve test compounds share key hydrophilic and hydrophobic interactions with simvastatin and atorvastatin. The residues participating in binding are the following: Glu96, Lys228, and Asp304 in CaHMGR; Glu93, Lys227, and Asp303 in CgHMGR; and Glu96, Lys229, and Asp305 in CauHMGR. Considering the binding interactions of either of the test compounds, at least one of these residues is shared with the binding interactions of the reference compounds, suggesting that all the test compounds and simvastatin and atorvastatin may have a similar mechanism of action. These types of interactions have been previously reported in other works for the HMGR of *H. sapiens* and CgHMGR [40,44,66].

Hydrophilic interactions involving a conventional hydrogen bond are predominant for the carbonyl groups of compounds **1a**, **2a**, **2c**, and **8a** with the amino acids Asp227, Lys228, and Asp304 of CaHMGR as well as with the amino acids Thr92 and Glu93 of CgHMGR. Similar interactions are observed for indolizine **5a** and enaminone **13a** with residues Gln303, Asn195, and Thr295 of CaHMGR. For more than one of the compounds assessed on CauHMGR, the principal interactions are both conventional hydrogen bond and carbon hydrogen with Thr94, Thr95, Lys229, Asn292, Thr295, and Asp305. In addition, the amino acids that predominate in the hydrophobic interactions with CaHMGR are Glu96 (π-anion), Met192 (π-sigma or π-alkyl), and Met194 (π-sigma or π-alkyl). Compounds **2a** and **8g** show π-alkyl interactions with Met189 in CgHMGR, and **1a**, **2a**, **2c**, **4l**, **8c**, and simvastatin display π-alkyl interactions with Pro306 in CauHMGR. The presence of the aromatic rings in the test compounds plays an important role in stabilizing the p interactions with the enzyme residues. According to the results, the test compounds can be considered as inhibitors of the HMGR enzymes of the *Candida* spp. herein evaluated, because they bind to amino acid residues of the catalytic site and share certain hydrophilic and hydrophobic interactions with simvastatin and atorvastatin.

## 3. Materials and Methods

### 3.1. General Information

Melting points were determined on a Krüss KSP 1N (KRÜSS GmbH, Hamburg, Germany) capillary melting point apparatus. IR spectra were recorded on FT-IR 2000 Perkin-Elmer (PerkinElmer, Waltham, MA, USA) and Bruker Vertex 70 (ATR-FT) (Bruker Corporation, Billerica, MA, USA) spectrophotometers. ^1^H and ^13^C NMR spectra were recorded on the following instruments: Varian Mercury (300 MHz) (Varian, Inc., Palo Alto, CA, USA), Bruker Ascend 400 (400 MHz) (Bruker Corporation, Billerica, MA, USA), Varian VNMR System (500 MHz) (Varian, Inc., Palo Alto, CA, USA), Bruker 600Avance III (600 MHz), and Bruker Avance III HD (750 MHz) (Bruker Corporation, Billerica, MA, USA), with CDCl_3_ as the solvent and TMS or CDCl_3_ as the internal standards. Signal assignments were based on 2D NMR spectra (HSQC, HMBC, and ROESY). High-resolution mass spectra (HRMS) (in electron impact mode) were obtained on Jeol JMS-GCMateII and Jeol JMS T100-LC AccuTOF DART (JEOL, Ltd., Tokyo, Japan) spectrometers. MW irradiation was emitted from a CEM MW reactor (CEM Corporation, Matthews, NC, USA). A Multi-Therm Benchmark, Model H5000-HC (Benchmark Scientific, Inc., Sayreville, NJ, USA) was utilized as a heating and cooling shaker in enzymatic stability assays. Yeast growth was quantified in a Multiskan™ GO microplate spectrophotometer (Thermo Fischer Scientific, Waltham, MA, USA) at 620 nm. Analytical thin-layer chromatography was carried out on 0.25 plates coated with silica gel 60 F254 (E. Merck, Darmstadt, Germany), which were visualized by a long- and short-wavelength UV lamp. Flash column chromatography was performed over silica gel (230–400 mesh, Natland International Co., Morrisville, NC, USA). Commercial reagents were used as received, and anhydrous solvents were obtained by a distillation process. Compounds **1a** and **8a**, **9a**, **9b**, and **10b** were prepared as reported [40,42].

### 3.2. Chemistry

*Ethyl 2-(2-formyl-1H-pyrrol-1-yl) acetate* (**8b**). At 0 °C and under N_2_ atmosphere, NaH (60%, 0.051 g, 1.27 mmol) was added to a solution of **6** (0.100 g, 1.05 mmol) and anhydrous DMF (2.0 mL), and the mixture was stirred for 20 min. Then, **7b** (0.210 g, 1.26 mmol) was added, and the solution was stirred at room temperature (rt) for 4 h. The reaction mixture was extracted with hexane/EtOAc (1:1, 15.0 mL). The organic layer was washed with brine (5.0 mL × 3) and dried (Na_2_SO_4_), and the solvent was removed under vacuum. The residue was purified by column chromatography over silica gel (hexane/EtOAc, 9:1) to obtain **8b** (0.179 g, 94%) as a pale violet oil. R*_f_* 0.64 (hexane/EtOAc, 1:1). IR (film): ῡ 3130, 2993, 2978, 2798, 1735, 1656, 1482, 1402, 1315, 1218, 1027, 752 cm^−1^. ^1^H NMR (400 MHz, CDCl_3_): δ 1.28 (t, *J* = 7.2 Hz, 3H, CO_2_CH_2_C*H*_3_), 4.22 (q, *J* = 7.2 Hz, 2H, CO_2_C*H*_2_CH_3_), 5.05 (s, 2H, C*H*_2_), 6.29 (dd, *J* = 4.0, 2.4 Hz, 1H, H-4′), 6.90–6.93 (m, 1H, H-5′), 6.98 (dd, *J* = 4.0, 1.6 Hz, 1H, H-3′), 9.52 (d, *J* = 0.8 Hz, 1H, C*H*O). ^13^C NMR (100 MHz, CDCl_3_): δ 14.1 (CO_2_CH_2_*C*H_3_), 50.2 (*C*H_2_), 61.6 (CO_2_*C*H_2_CH_3_), 110.2 (C-4′), 124.6 (C-3′) 131.7 (C-2′), 132.0 (C-5′), 168.3 (*C*O_2_CH_2_CH_3_), 179.7 (*C*HO). HRMS (EI): *m*/*z* [M^+^] calcd. for C_9_H_11_NO_3_: 181.0739; found: 181.0733.
*1-(2-Oxo-2-phenylethyl)-1H-pyrrole-2-carbaldehyde* (**8c**). Following the method described for **8b**, a mixture of **6** (0.100 g, 1.05 mmol), NaH (60%, 0.505 g, 1.26 mmol), and **7c** (0.251 g, 1.26 mmol) furnished **8c** (0.200 g, 89%) as white needles. R*_f_* 0.19 (hexane/EtOAc, 9:1); mp 114–115 °C. IR (film): ῡ 3112, 2938, 2812, 1702, 1648, 1402, 1366, 1330, 1222, 1080, 1019, 1005, 748, 690 cm^−1^. ^1^H NMR (300 MHz, CDCl_3_): δ 5.81 (s, 2H, C*H*_2_), 6.36 (dd, *J* = 4.2, 2.5 Hz, 1H, H-4), 6.94–6.98 (m, 1H, H-5), 7.04 (dd, *J* = 4.2, 1.5 Hz, 1H, H-3), 7.48–7.55 (m, 2H, H-3″), 7.59–7.67 (m, 1H, H-4″), 7.98–8.03 (m, H-2″), 9.51 (d, *J* = 0.9 Hz, 1H, C*H*O). ^13^C NMR (187.5 MHz, CDCl_3_): δ 54.7 (*C*H_2_), 110.3 (C-4), 124.7 (C-3), 128.0 (C-2″), 128.9 (C-3″), 131.6 (C-2), 132.5 (C-5), 133.8 (C-4″), 134.8 (C-1″), 179.8 (*C*HO), 192.9 (*C*O). HRMS (EI): *m*/*z* [M^+^] calcd. for C_13_H_11_NO_2_: 213.0790; found: 213.0790.
*1-(2-(3-Methoxyphenyl)-2-oxoethyl)-1H-pyrrole-2-carbaldehyde* (**8d**). Following the method described for **8b**, a mixture of **6** (0.060 g, 0.63 mmol), NaH (60%, 0.030 g, 0.76 mmol), and **7c** (0.174 g, 0.76 mmol) afforded **8d** (0.070 g, 45%) as white needles. R*_f_* 0.48 (hexane/EtOAc, 1:1); mp 106–107 °C. IR (KBr): ῡ 2939, 2799, 1694, 1649, 1591, 1403, 1260, 1192, 1024, 857, 754 cm^−1^. ^1^H NMR (750 MHz, CDCl_3_): δ 3.85 (s, 3H, C*H*_3_O), 5.79 (s, 2H, C*H*_2_), 6.35 (dd, *J* = 3.9, 2.6 Hz, 1H, H-4), 6.95 (br s, 1H, H-5), 7.03 (dd, *J* = 3.9, 1.5 Hz, 1H, H-3), 7.16 (ddd, *J* = 8.3, 2.6, 1.1 Hz, H-4″), 7.42 (t, *J =* 8.3 Hz, 1H, H-5″), 7.50 (dd, *J* = 2.6, 1.5 Hz, 1H, H-2″), 7.58 (dt, *J* = 7.5, 1.5 Hz, 1H, H-6″), 9.51 (d, *J* = 0.8 Hz, 1H, C*H*O). ^13^C NMR (187.5 MHz, CDCl_3_): δ 54.9 (*C*H_2_), 55.5 (*C*H_3_O), 110.3 (C-4), 112.4 (C-2″), 120.4 (C-4″), 120.5 (C-6″), 124.8 (C-3), 129.9 (C-5″), 131.6 (C-2), 132.5 (C-5), 136.1 (C-1″), 160.0 (C-3″), 179.8 (*C*HO), 192.8 (*C*O). HRMS (EI): *m*/*z* [M^+^] calcd. for C_14_H_13_NO_3_: 243.0895; found: 243.0891.
*1-(2-(4-Methoxyphenyl)-2-oxoethyl)-1H-pyrrole-2-carbaldehyde* (**8e**). Following the method described for **8b**, a mixture of **6** (0.200 g, 2.10 mmol), NaH (60%, 0.100 g, 2.52 mmol), and **7e** (0.577 g, 2.52 mmol) provided **8e** (0.413 g, 72%) as white needles. R*_f_* 0.70 (hexane/EtOAc, 1:1); mp 118–119 °C. IR (KBr): ῡ 2939, 1652, 1601, 1406, 1366, 1226, 1178, 1024, 846, 754 cm^−1^. ^1^H NMR (600 MHz, CDCl_3_): δ 3.88 (s,1H, C*H*_3_O), 5.77 (s, 2H, C*H*_2_), 6.35 (dd, *J* = 3.9, 2.4 Hz, 1H, H-4), 6.95 (br s, 1H, H-5), 6.96–6.99 (m, 2H, H-3″), 7.02 (dd, *J* = 3.9, 1.8 Hz, 1H, H-3), 7.97–8.00 (m, 2H, H-2″), 9.50 (d, *J* = 0.6 Hz, 1H, C*H*O). ^13^C NMR (150 MHz, CDCl_3_): δ 54.3 (*C*H_2_), 55.5 (*C*H_3_O), 110.2 (C-4), 114.1 (C-3″), 124.7 (C-3), 127.8 (C-1″), 130.4 (C-2″), 131.6 (C-2), 132.6 (C-5), 164.1 (C-4″), 179.8 (*C*HO), 191.3 (*C*O). HRMS (EI): *m*/*z* [M^+^] calcd. for C_14_H_13_NO_3_: 243.0895; found: 243.0885.
*1-(2-(3,4-Dimethoxyphenyl)-2-oxoethyl)-1H-pyrrole-2-carbaldehyde* (**8f**). Following the method described for **8b**, a mixture of **6** (0.030 g, 0.32 mmol), NaH (60%, 0.015 g, 0.38 mmol), and **7f** (0.098 g, 0.38 mmol) gave **8f** (0.070 g, 81%) as white needles. R*_f_* 0.13 (hexane/EtOAc, 1:1); 148–149 °C. IR (film): ῡ 3011, 1707, 1356, 1219, 1417, 762 cm^−1^. ^1^H NMR (600 MHz, CDCl_3_): δ 3.93 (s, 3H, C*H*_3_O), 3.97 (s, 3H, C*H*_3_O), 5.79 (s, 2H, C*H*_2_), 6.35 (dd, *J* = 3.9, 2.7 Hz, 1H, H-4), 6.94 (d, *J* = 8.1 Hz, 1H, H-5″), 6.96 (br s, 1H, H-5), 7.03 (dd, *J* = 3.9, 1.8 Hz, 1H, H-3), 7.53 (d, *J* = 2.1 Hz, 1H, H-2″), 7.66 (dd, *J* = 8.1, 2.1 Hz, 1H, H-6″), 9.52 (d, *J* = 0.6 Hz, 1H, C*H*O). ^13^C NMR (150 MHz, CDCl_3_): δ 54.2 (*C*H_2_), 56.0 (*C*H_3_O), 56.1 (*C*H_3_O), 110.2 (C-2″, C-4), 110.3 (C-5″), 122.6 (C-6″), 124.8 (C-3), 128.0 (C-1″), 131.7 (C-2), 132.6 (C-5), 149.3 (C-3″), 154.0 (C-4″), 179.9 (*C*HO), 191.5 (*C*O). HRMS (EI): *m*/*z* [M^+^] calcd. for C_15_H_15_NO_4_: 273.1001; found: 273.1002.
*2-(2-Formyl-1H-pyrrol-1-yl)acetonitrile* (**8g**). Following the method described for **8b**, a mixture of **6** (0.050 g, 0.53 mmol), NaH (60%, 0.025 g, 0.63 mmol), and **7g** (0.076 g, 0.63 mmol) produced **8g** (0.036 g, 51%) as pale violet oil. R*_f_* 0.64 (hexane/EtOAc, 1:1). IR (film): ῡ 3113, 2864, 2253, 1652, 1475, 1406, 1362, 1308, 1222, 1028, 748 cm^−1^. ^1^H NMR (750 MHz, CDCl_3_): δ 5.35 (s, 2H, C*H*_2_), 6.34 (dd, *J* = 3.9, 2.6 Hz, 1H, H-4′), 7.00 (dd, *J* = 3.9, 1.5 Hz, 1H, H-3′), 7.08 (br s, 1H, H-5′), 9.57 (d, *J* = 0.8 Hz, 1H, C*H*O). ^13^C NMR (187.5 MHz, CDCl_3_): δ 36.2 (*C*H_2_), 111.5 (C-4′), 114.5 (*C*N), 125.2 (C-3′), 130.7 (C-5′), 131.0 (C-2′), 179.9 (*C*HO). HRMS (EI): *m*/*z* [M^+^] calcd. for C_7_H_6_N_2_O: 134.0480; found: 134.0477.
*Ethyl (Z)-3-(dimethylamino)-2-(2-formyl-1H-pyrrol-1-yl)acrylate* (**1b**). In a threaded ACE glass pressure tube equipped with a magnetic stirring bar and sealed with a Teflon screw cap, a mixture of **8b** (0.100 g, 0.55 mmol) and DMFDMA (0.329 g, 2.75 mmol) in anhydrous DMF (2.0 mL) was heated at 100 °C for 20 h. A mixture of CH_2_Cl_2_/toluene (10.0 mL/1.0 mL) was added, and the solvent was removed under vacuum. The residue was purified by column chromatography over silica gel (hexane/EtOAc, 7:3), resulting in **1b** (0.057 g, 44%) as a yellow oil. R*_f_* 0.16 (hexane/EtOAc, 1:1). IR (film): ῡ 2934, 1663, 1618, 1303, 1209, 1078, 749 cm^−1^. ^1^H NMR (750 MHz, CDCl_3_): δ 1.16 (t, *J* = 6.8 Hz, 3H, CO_2_CH_2_C*H*_3_), 2.65 (br, 6H, N(C*H*_3_)_2_), 4.04–4.16 (m, 2H, CO_2_C*H*_2_CH_3_), 6.29 (dd, *J* = 3.8, 2.3 Hz, H-4′), 6.82 (br t, *J* = 2.3 Hz, 1H, H-5′), 7.01 (dd, *J* = 3.8, 1.5 Hz, H-3′), 7.51 (s, 1H, H-3), 9.56 (s, 1H, *C*HO). ^13^C NMR (187.5 MHz, CDCl_3_): δ 14.5 (CO_2_CH_2_*C*H_3_), 34.9 (N(*C*H_3_)_2_), 60.1 (CO_2_*C*H_2_CH_3_), 97.9 (C-2), 110.2 (C-4′), 120.9 (C-3′), 134.2 (C-5′), 134.9 (C-2′), 146.0 (C-3), 166.9 (*C*O_2_Et), 179.8 (*C*HO). HRMS (EI): *m*/*z* [M^+^] calcd. for C_12_H_16_N_2_O_3_: 236.1161; found: 236.1159.
*(Z)-1-(1-(Dimethylamino)-3-oxo-3-phenylprop-1-en-2-yl)-1H-pyrrole-2-carbaldehyde* (**2a**). Following the method described for **1b**, a mixture of **8c** (0.030 g, 0.14 mmol) and DMFDMA (0.084 g, 0.70 mmol) in anhydrous DMF (1.0 mL) delivered **2a** (0.020 g, 53%) as a yellow oil. R*_f_* 0.16 (hexane/EtOAc, 1:1). IR (film): ῡ 3097, 2928, 1637, 1543, 1413, 1388, 1323, 1095, 950, 878, 766, 708 cm^−1^. ^1^H NMR (750 MHz, CDCl_3_): δ 2.68 (br, 6H, N(C*H*_3_)_2_), 6.33 (dd, *J* = 3.9, 2.6 Hz, 1H, H-4), 6.89 (br s, 1H, H-5), 7.02 (dd, *J* = 3.9, 1.5 Hz, 1H, H-3), 7.31–7.36 (m, 3H, H-1′, H-3″), 7.37–7.40 (m, 1H, H-4″), 7.48–7.52 (m, 2H, H-2″), 9.60 (d, *J* = 0.9 Hz, 1H, C*H*O). ^13^C NMR (187.5 MHz, CDCl_3_): δ 29.6 (br, N(*C*H_3_)_2_), 110.6 (C-2′, C-4), 122.6 (C-3), 127.90 (C-2″), 127.93 (C-3″), 129.9 (C-4″), 134.39 (C-2), 134.43 (C-5), 139.9 (C-1″), 150.2 (C-1′), 179.4 (*C*HO), 190.9 (*C*O). HRMS (EI): *m*/*z* [M^+^] calcd. for C_16_H_16_N_2_O_2_: 268.1212; found: 268.1213.
*(Z)-1-(1-(Dimethylamino)-3-(3-methoxyphenyl)-3-oxoprop-1-en-2-yl)-1H-pyrrole-2-carbaldehyde* (**2b**). Following the method described for **1b**, a mixture of **8d** (0.050 g, 0.21 mmol) and DMFDMA (0.125 g, 1.05 mmol) in anhydrous DMF (1.0 mL) formed **2b** (0.035 g, 56%) as a pale violet oil. R*_f_* 0.45 (hexane/EtOAc, 1:1). IR (film): ῡ 2925, 1663, 1588, 1424, 1322, 1042, 748 cm^−1^. ^1^H NMR (500 MHz, CDCl_3_): δ 2.70 (br, 6H, N(C*H*_3_)_2_), 3.79 (s, 3H, C*H*_3_O), 6.31–6.34 (m, 1H, H-4), 6.88 (br s, 1H, H-5), 6.92 (dm, *J* = 8.0 Hz, 1H, H-4″), 7.01–7.04 (m, 2H, H-2″, H-3), 7.06–7.10 (m, 1H, H-6″), 7.23 (t, *J* = 8.0 Hz, 1H, H-5″), 7.37 (br s, 1H, H-1′), 9.60 (s, 1H, C*H*O). ^13^C NMR (125 MHz, CDCl_3_): δ 36.5 (br, N(*C*H_3_)_2_), 55.2 (*C*H_3_O), 110.6 (C-4), 111.1 (C-2′), 112.7 (C-2″), 116.3 (C-4″), 120.3 (C-6″), 122.5 (C-3), 128.9 (C-5″), 129.5 (C-2), 134.4 (C-5), 141.1 (C-1″), 150.4 (C-1′), 159.2 (C-3″), 179.4 (*C*HO), 190.6 (*C*O). HRMS (EI): *m*/*z* [M^+^] calcd. for C_17_H_18_N_2_O_3_: 298.1317; found: 298.1311.
*(Z)-1-(1-(Dimethylamino)-3-(4-methoxyphenyl)-3-oxoprop-1-en-2-yl)-1H-pyrrole-2-carbaldehyde* (**2c**). Following the method described for **1b**, a mixture of **8e** (0.100 g, 0.41 mmol) and DMFDMA (0.245 g, 2.05 mmol) in anhydrous DMF (1.0 mL) provided **2c** (0.111 g, 90%) as a yellow oil. R*_f_* 0.16 (hexane/EtOAc, 1:1). IR (film): ῡ 2939, 1663, 1600, 1584, 1560, 1386, 1325, 1243, 1096, 1021, 840, 765, 738 cm^−1^. ^1^H NMR (500 MHz, CDCl_3_): δ 2.67 (br, 6H, N(C*H*_3_)_2_), 3.79 (s, 3H, C*H*_3_O), 6.31 (dd, *J* = 4.2, 2.4 Hz, 1H, H-4), 6.80 (d, *J* = 8.4 Hz, 2H, H-3″), 6.87 (br s, 1H, H-5), 7.01 (dd, *J* = 4.2, 1.8 Hz, 1H, H-3), 7.35 (s, 1H, H-1′), 7.45 (d, *J* = 8.4 Hz, 1H, H-2″), 9.56 (s, 1H, C*H*O). ^13^C NMR (125 MHz, CDCl_3_): δ 34.8 (br, N(*C*H_3_)_2_), 55.2 (*C*H_3_O), 110.1 (C-2′), 110.6 (C-4), 113.2 (C-3″), 122.3 (C-3), 130.0 (C-2″), 132.1 (C-1″), 134.3 (C-2), 134.4 (C-5), 149.7 (C-1′), 161.2 (C-4″), 179.5 (*C*HO), 189.7 (*C*O). HRMS (EI): *m*/*z* [M^+^] calcd. for C_17_H_18_N_2_O_3_: 298.1317; found: 298.1314.
*(Z)-1-(3-(3,4-Dimethoxyphenyl)-1-(dimethylamino)-3-oxoprop-1-en-2-yl)-1H-pyrrole-2-carbaldehyde* (**2d**). Following the method described for **1b**, a mixture of **8f** (0.050 g, 0.18 mmol) and DMFDMA (0.109 g, 0.90 mmol) in anhydrous DMF (1.0 mL) afforded **2d** (0.050 g, 83%) as a brown solid. R*_f_* 0.03 (hexane/EtOAc, 1:1); 138–140 °C. IR (film): ῡ 2929, 1738, 1639, 1564, 1509, 1410, 1311, 1096, 1018, 748 cm^−1^. ^1^H NMR (600 MHz, CDCl_3_): δ 2.69 (br, 6H, N(C*H*_3_)_2_), 3.82 (C*H*_3_O), 3.87 (C*H*_3_O), 6.31 (dd, *J* = 4.0, 2.1 Hz, 1H, H-4), 6.76 (d, *J* = 8.1 Hz, 1H, H-5″), 6.87 (br s, 1H, H-5), 7.02 (dd, *J* = 4.0, 1.8 Hz, 1H, H-3), 7.04 (br s, 1H, H-2″), 7.09 (br d, *J* = 8.1 Hz, 1H, H-6″), 7.43 (s, 1H, H-1′), 9.58 (C*H*O). ^13^C NMR (150 MHz, CDCl_3_): δ 42.0 (br, N(*C*H_3_)_2)_, 55.8 (*C*H_3_O), 55.9 (*C*H_3_O), 110.00 (C-5″), 110.02 (C-2′), 110.6 (C-4), 111.2 (C-2″), 121.7 (C-6″), 122.2 (C-3), 132.2 (C-1″), 134.3 (C-5), 134.5 (C-2), 148.4 (C-3″), 149.6 (C-1′), 150.1 (C-4″), 179.4 (*C*HO), 189.4 (*C*O). HRMS (EI): *m*/*z* [M^+^] calcd. for C_18_H_20_N_2_O_4_: 328.1423; found: 328.1426.
*(Z)-3-(Dimethylamino)-2-(2-formyl-1H-pyrrol-1-yl)acrylonitrile* (**3a**). *(E)-3-(Dimethylamino)-2-(2-formyl-1H-pyrrol-1-yl)acrylonitrile* (**3a’**). Following the method described for **1b**, a mixture of **8g** (0.077 g, 0.57 mmol) and DMFDMA (0.339 g, 2.85 mmol) in anhydrous DMF (2.0 mL) generated an inseparable mixture of **3a**/**3a’** (54:46, 0.030 g, 28%) as a pale violet oil. R*_f_* 0.16 (hexane/EtOAc, 1:1). IR (film): ῡ 3095, 2922, 2803, 2184, 1650, 1365, 1130, 772 cm^−1^. ^1^H NMR (600 MHz, CDCl_3_): δ 2.63 (br, 6H, N(C*H*_3_)_2_), 3.16 (s, 6H, N(C*H*_3_)_2_), 6.25 (dd, *J* = 4.2, 2.4 Hz, 1H, H-4′), 6.31 (dd, *J* = 4.2, 2.4 Hz, 1H, H-4′), 6.68 (s, 1H, H-3), 6.73 (s, 1H, H-3), 6.87–6.89 (m, 1H, H-5′), 6.91–6.93 (m, 1H, H-5′), 6.96 (dd, *J* = 4.2, 1.8 Hz, 1H, H-3′), 6.99 (dd, *J* = 4.2, 1.8 Hz, 1H, H-3′), 9.63 (s, 1H, C*H*O), 9.66 (s, 1H, C*H*O). ^13^C NMR (150 MHz, CDCl_3_): δ 42.3 (br, N(*C*H_3_)_2_), 78.6 (C-2), 78.8 (C-2), 110.7 (C-4′), 111.2 (C-4′), 118.5 (*C*N), 120.24 (*C*N), 122.5 (2C-3′), 133.0 (C-5′), 133.2 (C-2′), 133.8 (C-5′), 134.5 (C-2′), 147.4 (C-3), 151.0 (C-3), 178.9 (*C*HO), 179.2 (*C*HO). HRMS (EI): *m*/*z* [M^+^] calcd. for C_10_H_11_N_3_O: 189.0902; found: 189.0900.
*Methyl pyrrolo[1,2-a]pyrazine-4-carboxylate* (**4a**). **Method A:** In a threaded ACE glass pressure tube equipped with a magnetic stirring bar and sealed with a Teflon screw cap, a mixture of NH_4_OAc (0.052 g, 0.675 mmol) and Li_2_CO_3_ (0.050 g, 0.675 mmol) in anhydrous DMF (1.0 mL) was stirred at rt for 20 min. Then, **1a** (0.050 g, 0.225 mmol) was added, and the solution was heated at 70 °C for 4 h. The mixture was diluted with hexane/EtOAc (1:1, 15 mL) and washed with brine (5.0 mL × 3). The organic layer was dried (Na_2_SO_4_), and the solvent was removed under vacuum. The residue was purified by column chromatography over silica gel (hexane/EtOAc, 8:2) to give **4a** (0.036 g, 90%) as a yellow solid. R*_f_* 0.58 (hexane/EtOAc, 1:1); mp 93–95 °C.
**Method B:** In a threaded ACE glass pressure tube equipped with a magnetic stirring bar and sealed with a Teflon screw cap, a mixture of **8a** (0.030 g, 0.18 mmol), DMFDMA (0.114 g, 0.90 mmol), and NH_4_OAc (0.042 g, 0.54 mmol) was heated at 100 °C for 24 h. The mixture was suspended in CH_2_Cl_2_/PhMe (10:1, 11 mL), and the solvent was removed under vacuum. The residue was purified by column chromatography over silica gel (hexane/EtOAc, 1:1) to furnish **4a** (0.018 g, 57%) as a yellow solid. R*_f_* 0.58 (hexane/EtOAc, 1:1); mp 93–95 °C. IR (film): ῡ 3168, 2954, 1720, 1621, 1441, 1430, 1306, 1217, 1203, 1177, 1112, 764, 744 cm^−1^. ^1^H NMR (500 MHz, CDCl_3_): δ 4.01 (s, 3H, CO_2_C*H*_3_), 7.02 (s, 2H, H-7, H-8), 8.46 (s, 1H, H-3), 8.74 (s, 1H, H-6), 8.92 (s, 1H, H-1). ^13^C NMR (125 MHz, CDCl_3_): δ 52.4 (CO_2_*C*H_3_), 106.7 (C-8), 116.6 (C-7), 118.3 (C-4), 118.9 (C-6), 129.9 (C-8a), 134.8 (C-3), 148.1 (C-1), 163.3 (*C*O_2_CH_3_). HRMS (EI): *m*/*z* [M^+^] calcd. for C_9_H_8_N_2_O_2_: 176.0586; found: 176.0592.
*Ethyl pyrrolo[1,2-a]pyrazine-4-carboxylate* (**4b**). Following method A described for **4a**, a mixture of **1b** (0.030 g, 0.13 mmol), NH_4_OAc (0.030 g, 0.39 mmol), and Li_2_CO_3_ (0.028 g, 0.39 mmol) was heated at 80 °C for 3 h to obtain **4b** (0.016 g, 66%) as a yellow solid. R*_f_* 0.67 (hexane/EtOAc, 1:1); mp 68–70 °C. IR (film): ῡ 3177, 3097, 2848, 1699, 1427, 1287, 1178, 1088, 1033, 734 cm^−1^. ^1^H NMR (750 MHz, CDCl_3_): δ 1.45 (t, *J* = 7.4 Hz, CO_2_CH_2_C*H*_3_), 4.47 (q, *J* = 7.4 Hz, CO_2_C*H*_2_CH_3_), 7.01–7.03 (m, 2H, H-7, H-8), 8.46 (s, 1H, H-3), 8.73–8.74 (m, 1H, H-6), 8.91 (s, 1H, H-1). ^13^C NMR (187.5 MHz, CDCl_3_): δ 14.3 (CO_2_CH_2_*C*H_3_), 61.6 (CO_2_*C*H_2_CH_3_), 106.6 (C-8), 116.5 (C-7), 118.9 (C-6), 129.9 (C-8a), 134.6 (C-3), 134.8 (C-4), 148.0 (C-1), 162.8 (*C*O_2_CH_2_CH_3_). HRMS (EI): *m*/*z* [M^+^] calcd. for C_10_H_10_N_2_O_2_: 190.0742; found: 190.0742.
*Phenyl(pyrrolo[1,2-a]pyrazin-4-yl)methanone* (**4c**). Following method A described for **4a**, a mixture of **2a** (0.050 g, 0.187 mmol), NH_4_OAc (0.043 g, 0.56 mmol), and Li_2_CO_3_ (0.041 g, 0.56 mmol) was heated at 80 °C for 3 h to give **4c** (0.036 g, 87%) as a yellow solid. R*_f_* 0.48 (hexane/EtOAc, 1:1); mp 61–62 °C. Following method B described for **4a**, a mixture of **8c** (0.030 g, 0.141 mmol), DMFDMA (0.084 g, 0.70 mmol), and NH_4_OAc (0.054 g, 0.70 mmol) produced **4c** (0.025 g, 80%) as a yellow solid. R*_f_* 0.48 (hexane/EtOAc, 1:1); mp 61–62 °C. IR (film): ῡ 3101, 3040, 2924, 1633, 1467, 1290, 1203, 1052, 882, 741 cm^−1^. ^1^H NMR (750 MHz, CDCl_3_): δ 7.10 (d, *J* = 1.5 Hz, 2H, H-7′, H-8′), 7.53 (t, *J* = 7.5 Hz, 2H, H-3″), 7.63 (t, *J* = 7.5 Hz, 1H, H-4″), 7.81 (br d, *J* = 7.5 Hz, 2H, H-2″), 8.10 (s, 1H, H-3′), 8.92 (br m, 1H, H-6′), 8.94 (s, 1H, H-1′). ^13^C NMR (187.5 MHz, CDCl_3_): δ 107.4 (C-8′), 117.1 (C-7′), 119.6 (C-6′), 124.5 (C-8a’), 128.5 (C-3″), 129.6 (C-2″), 130.1 (C-4′), 132.5 (C-4″), 137.8 (C-1″), 139.0 (C-3′), 148 (C-1′), 190.9 (*C*O). HRMS (EI): *m*/*z* [M^+^] calcd. for C_14_H_10_N_2_O: 222.0793; found: 222.0794.
*(3-Methoxyphenyl)(pyrrolo[1,2-a]pyrazin-4-yl)methanone* (**4d**). Following method A described for **4a**, a mixture of **2b** (0.030 g, 0.10 mmol), NH_4_OAc (0.023 g, 0.30 mmol), and Li_2_CO_3_ (0.022 g, 0.30 mmol) provided **4d** (0.021 g, 83%) as a yellow solid. R*_f_* 0.39 (hexane/EtOAc, 1:1); mp 88–89 °C. IR (film): ῡ 2970, 1734, 1594, 1424, 1298, 1250, 1171, 1028, 854, 741 cm^−1^. ^1^H NMR (600 MHz, CDCl_3_): δ 3.88 (s, 3H, C*H*_3_O), 7.09 (br s, 2H, H-7′, H-8′), 7.17 (dd, *J* = 8.0, 2.6 Hz, 1H, H-4″), 7.34 (br d, *J* = 1.2 Hz, 1H, H-2″), 7.36 (br d, *J* = 8.0 Hz, 1H, H-6″), 7.43 (t, *J* = 8.0 Hz, 1H, H-5″), 8.13 (s, 1H, H-3′), 8.91 (s, 1H, H-6′), 8.94 (s, 1H, H-1′). ^13^C NMR (150 MHz, CDCl_3_): δ 55.4 (*C*H_3_O), 107.5 (C-8′), 114.2 (C-2″), 117.2 (C-7′), 118.9 (C-4″), 119.6 (C-6′), 122.2 (C-6″), 124.6 (C-8a’), 129.5 (C-5″), 130.1 (C-4′), 138.9 (C-3′), 139.0 (C-1″), 147.9 (C-1′), 159.7 (C-3″), 190.6 (*C*O). HRMS (EI): *m*/*z* [M^+^] calcd. for C_15_H_12_N_2_O_2_: 252.0899; found: 252.0900.
*(4-Methoxyphenyl)(pyrrolo[1,2-a]pyrazin-4-yl)methanone* (**4e**). Following method A described for **4a**, a mixture of **2c** (0.050 g, 0.168 mmol), NH_4_OAc (0.039 g, 0.50 mmol), and Li_2_CO_3_ (0.037 g, 0.50 mmol) formed **4e** (0.036 g, 84%) as a yellow solid. R*_f_* 0.29 (hexane/EtOAc, 1:1); mp 138–139 °C. IR (KBr): ῡ 2928, 1739, 1590, 1507, 1301, 1265, 1167, 1109, 1030, 845, 759, 719 cm^−1^. ^1^H NMR (600 MHz, CDCl_3_): δ 3.91 (s, 3H, C*H*_3_O), 7.00–7.03 (m, 2H, H-3″), 7.05 (br s, 1H, H-8′), 7.06 (br s, 1H, H-7′), 7.84–7.87 (m, 2H, H-2″), 8.05 (br s, 1H, H-3′), 8.74 (s, 1H, H-6′), 8.92 (s, 1H, H-1′). ^13^C NMR (150 MHz, CDCl_3_): δ 55.6 (*C*H_3_O), 106.9 (C-8′), 113.9 (C-3″), 116.8 (C-7′), 119.0 (C-6′), 124.8 (C-8a’), 130.0 (C-4′), 130.1 (C-1″), 132.2 (C-2″), 137.4 (C-3′), 147.7 (C-1′), 163.6 (C-4″), 189.3 (*C*O). HRMS (EI): *m*/*z* [M^+^] calcd. for C_15_H_12_N_2_O_2_: 252.0899; found: 252.0899.
*(3,4-Dimethoxyphenyl)(pyrrolo[1,2-a]pyrazin-4-yl)methanone* (**4f**). Following method A described for **4a**, a mixture of **2d** (0.079 g, 0.24 mmol), NH_4_OAc (0.056 g, 0.72 mmol), and Li_2_CO_3_ (0.053 g, 0.72 mmol) afforded **4f** (0.041 g, 60%) as a yellow solid. R*_f_* 0.16 (hexane/EtOAc, 1:1); mp 148–149 °C. IR (KBr): ῡ 3158, 2943, 1632, 1594, 1509, 1414, 1260, 1171, 1014, 772 cm^−1^. ^1^H NMR (750 MHz, CDCl_3_): δ 3.95 (s, 3H, C*H*_3_O), 3.97 (s, 3H, C*H*_3_O), 6.94 (d, *J* = 8.8 Hz, 1H, H-5″), 7.04–7.06 (m, 2H, H-7′, H-8′), 7.44–7.47 (m, H-2″, H-6″), 8.06 (s, 1H, H-3′), 8.69 (br s, 1H, H-6′), 8.91 (s, 1H, H-1′). ^13^C NMR (187.5 MHz, CDCl_3_): δ 56.0 (*C*H_3_O), 56.1 (*C*H_3_O), 106.9 (C-8′), 110.0 (C-5″), 111.8 (C-2″), 116.8 (C-7′), 118.9 (C-6′), 124.7 (C-8a’), 124.8 (C-6″), 129.9 (C-4′), 130.1 (C-1″), 137.2 (C-3′), 147.6 (C-1′), 149.2 (C-3″), 153.4 (C-4″), 189.2 (*C*O). HRMS (EI): *m*/*z* [M^+^] calcd. for C_16_H_14_N_2_O_3_: 282.1005; found: 282.1004.
*Pyrrolo[1,2-a]pyrazine-4-carbonitrile* (**4g**). Following method A described for **4a**, a mixture of **3a** (0.032 g, 0.169 mmol), NH_4_OAc (0.039 g, 0.51 mmol), and Li_2_CO_3_ (0.038 g, 0.51 mmol) furnished **4g** (0.013 g, 53%) as a brown solid. R*_f_* 0.55 (hexane/EtOAc, 1:1); mp 128–130 °C. IR (film): ῡ 3010, 2600, 1741, 1366, 1212 cm^−1^. ^1^H NMR (600 MHz, CDCl_3_): δ 7.13 (s, 2H, H-7, H-8), 7.84 (s, 1H, H-6), 8.08 (br s, 1H, H-3), 9.00 (br s, 1H, H-1). ^13^C NMR (100 MHz, CDCl_3_): δ 104.5 (*C*N), 107.9 (C-8), 112.8 (C-4), 116.6 (C-6), 117.0 (C-7), 127.9 (C-8a), 136.3 (C-3), 148.1 (C-1). HRMS (EI): *m*/*z* [M^+^] calcd. for C_8_H_5_N_3_: 143.0483; found: 143.0485.
*Phenyl(pyrrolo[1,2-a]pyrazin-4-yl)methanol* (**4h**). In a round-bottom flask equipped with a magnetic stirring bar, NaBH_4_ (0.051 g, 1.33 mmol) was slowly added to a solution of **4c** (0.030 g, 0.13 mmol) in anhydrous THF (5.0 mL). After heating the reaction mixture at 60 °C for 2 h, MeOH (8.0 mL) was added dropwise and stirred at rt for 5.0 min. Then, a saturated aqueous solution of NH_4_Cl (8.0 mL) was added and stirred at rt for 20 min. The mixture was extracted with hexane/EtOAc (1:1, 15 mL) and brine (3 × 5.0 mL). The organic layer was dried (Na_2_SO_4_), and the solvent was removed under vacuum. The residue was purified by column chromatography over silica gel (hexane/EtOAc, 7:3), resulting in **4h** (0.014 g, 48%) as a yellow solid. R*_f_* 0.10 (hexane/EtOAc, 1:1); mp 150–152 °C. IR (film): ῡ 3042, 2820, 1618, 1451, 1318, 1048, 727, 704 cm^−1^. ^1^H NMR (600 MHz, CDCl_3_): δ 3.10 (br, 1H, OH), 5.98 (s, 1H, C*H*OH), 6.80 (s, 2H, H-7′, H-8′), 7.30–7.39 (m, 4H, H-6′, H-3″, H-4″), 7.43 (br s, 1H, H-3′), 7.44–7.46 (m, 2H, H-2″), 8.58 (s, 1H, H-1′). ^13^C NMR (187.5 MHz, CDCl_3_): δ 71.9 (*C*HOH), 104.5 (C-8′), 114.6 (C-6′), 115.3 (C-7′), 125.5 (C-3′), 126.6 (C-2″), 128.5 (C-4″), 128.83 (C-3″), 128.88 (C-8a’), 130.2 (C-4′), 138.6 (C-1″), 144.3 (C-1′). HRMS (EI): *m*/*z* [M^+^] calcd. for C_14_H_12_N_2_O: 224.0950; found: 224.0948.
*Methyl 2-(4-bromo-2-formyl-1H-pyrrol-1-yl)acetate* (**8h**). In a round-bottom flask at 0 °C with a magnetic stirring bar, a solution of NBS (0.080 g, 0.45 mmol) in anhydrous CH_2_Cl_2_ (5.0 mL) was added dropwise to a solution of **8a** (0.05 g, 0.30 mmol) in anhydrous DMF (5.0 mL). The mixture was stirred at rt for 2.0 h before adding a 10% aqueous solution of NaHSO_3_ (1.0 mL). It was then washed with brine (3 × 5.0 mL), the organic phase was dried (Na_2_SO_4_), and the solvent was removed under vacuum. The residue was purified by column chromatography over silica gel (hexane/EtOAc, 8:2) to afford **8h** (0.066 g, 90%) as a violet oil. R*_f_* 0.74 (hexane/EtOAc, 1:1). IR (film): ῡ 2922, 1692, 1661, 1393, 1215, 998, 768 cm^−1^. ^1^H NMR (500 MHz, CDCl_3_): δ 3.76 (s, 3H, CO_2_C*H*_3_), 5.01 (s, 2H, C*H*_2_), 6.90 (br s, 1H, H-5′), 6.95 (d, *J* = 2.0 Hz, 1H, H-3′), 9.45 (d, *J* = 0.5 Hz, 1H, C*H*O). ^13^C NMR (125 MHz, CDCl_3_): δ 50.1 (*C*H_2_), 52.6 (CO_2_*C*H_3_), 97.5 (C-4′), 125.2 (C-3′) 131.2 (C-5′), 131.6 (C-2′), 168.2 (*C*O_2_CH_3_), 179.3 (*C*HO). HRMS (EI): *m*/*z* [M^+^] calcd. for C_8_H_8_BrNO_3_: 244.9688; found: 244.9680.
*Methyl (Z)-2-(4-bromo-2-formyl-1H-pyrrol-1-yl)-3-(dimethylamino)acrylate* (**1c**). Following the method described for **1b**, a mixture of **8h** (0.050 g, 0.20 mmol) and DMFDMA (0.121 g, 1.02 mmol) in anhydrous DMF (1.0 mL) gave **1c** (0.041 g, 66%) as a pale violet oil. R*_f_* 0.12 (hexane/EtOAc, 7:3). IR (film): ῡ 2921, 1666, 1621, 1212, 1084, 922, 755 cm^−1^. ^1^H NMR (600 MHz, CDCl_3_): δ 2.67 (br, 6H, N(C*H_3_*)_2_), 3.62 (s, 3H, CO_2_C*H*_3_), 6.81 (br s, 1H, H-5′), 6.99 (br s, 1H, H-3′), 7.50 (s, 1H, H-3), 9.49 (s, 1H, C*H*O). ^13^C NMR (125 MHz, CDCl_3_): δ 34.7 (N(*C*H_3_)_2_), 51.6 (CO_2_*C*H_3_), 96.6 (C-2), 98.2 (C-4′), 121.6 (C-3′), 132.9 (C-5′), 134.8 (C-2′), 146.4 (C-3), 166.9 (*C*O_2_CH_3_), 179.1 (*C*HO). HRMS (EI): *m*/*z* [M^+^] calcd. for C_11_H_13_BrN_2_O_3_: 300.0110; found: 300.0108.
*Methyl 7-bromopyrrolo[1,2-a]pyrazine-4-carboxylate* (**4i**). Following method A described for **4a**, a mixture of **1c** (0.030 g, 0.10 mmol), NH_4_OAc (0.031 g, 0.40 mmol), and Li_2_CO_3_ (0.029 g, 0.40 mmol) provided **4i** (0.017 g, 74%) as a yellow solid. R*_f_* 0.38 (hexane/EtOAc, 1:1); mp 93–95 °C. IR (film): ῡ 3173, 3126, 2957, 1720, 1442, 1308, 1200, 1175 cm^−1^. ^1^H NMR (500 MHz, CDCl_3_): δ 4.01 (s, 3H, CO_2_C*H*_3_), 7.10 (br s, 1H, H-8), 8.45 (br s, 1H, H-3), 8.78 (s, 1H, H-6), 8.87 (br s, 1H, H-1). ^13^C NMR (125 MHz, CDCl_3_): δ 52.8 (CO_2_*C*H_3_), 107.2 (C-7), 109.2 (C-8), 117.7 (C-4), 119.5 (C-6), 129.6 (C-8a), 133.7 (C-3), 145.9 (C-1), 162.5 (*C*O_2_CH_3_). HRMS (EI): *m*/*z* [M^+^] calcd. for C_9_H_7_BrN_2_O_2_: 253.9691; found: 253.9681.
*Methyl 6-bromopyrrolo[1,2-a]pyrazine-4-carboxylate* (**4j**). In a round-bottom flask at 0 °C with a magnetic stirring bar, a solution of NBS (0.040 g, 0.23 mmol) in anhydrous CH_2_Cl_2_ (5.0 mL) was added dropwise to a solution of **4a** (0.040 g, 0.23 mmol) in anhydrous CH_2_Cl_2_ (5.0 mL). The mixture was stirred at rt for 2.0 h before adding a 10% aqueous solution of NaHSO_3_ (1.0 mL). It was then washed with brine (3 × 5.0 mL), the organic phase was dried (Na_2_SO_4_), and the solvent was removed under vacuum. The residue was purified by column chromatography over silica gel (hexane/EtOAc, 1:1) to obtain **4j** (0.032 g, 55%) as a pale orange solid. R*_f_* 0.32 (hexane/EtOAc, 1:1); mp 115–117 °C. IR (film): ῡ 3144, 2953, 1724, 1417, 1294, 1175, 957, 857, 773 cm^−1^. ^1^H NMR (600 MHz, CDCl_3_): δ 4.00 (s, 3H, CO_2_C*H*_3_), 7.03 (d, *J* = 2.4 Hz, 1H, H-8), 8.46 (s, 1H, H-3), 8.67 (dd, 1H, *J* = 2.4, 0.6 Hz, H-7), 8.92 (br s, 1H, H-1). ^13^C NMR (150 MHz, CDCl_3_): δ 52.6 (CO_2_*C*H_3_), 94.3 (C-6), 118.5 (C-8), 118.8 (C-7), 127.1 (C-4), 129.5 (C-8a), 135.2 (C-3), 147.2 (C-1), 162.8 (*C*O_2_CH_3_). HRMS (EI): *m*/*z* [M^+^] calcd. for C_9_H_7_BrN_2_O_2_: 253.9691; found: 253.9701.
*Methyl 6,7-dibromopyrrolo[1,2-a]pyrazine-4-carboxylate* (**4k**). Following the method described for **4j**, a mixture of **4a** (0.030 g, 0.17 mmol) and NBS (0.063 g, 0.36 mmol) produced **4k** (0.023 g, 40%) as an orange solid. R*_f_* 0.61 (hexane/EtOAc, 1:1); mp 106–108 °C. IR (film): ῡ 3123, 2952, 2918, 2850, 1733, 1601, 1464, 1420, 1312, 1294, 1200, 1172, 1095, 862, 753 cm ^−1^. ^1^H NMR (500 MHz, CDCl_3_): δ 4.02 (s, 3H, CO_2_C*H*_3_), 7.04 (s, 1H, H-8), 8.00 (s, 1H, H-3), 8.80 (s, 1H, H-1). ^13^C NMR (125 MHz, CDCl_3_): δ 53.0 (CO_2_*C*H_3_), 95.0 (C-6), 99.6 (C-7), 121.1 (C-4), 122.0 (C-8), 128.7 (C-8a), 133.1 (C-3), 145.8 (C-1), 161.6 (*C*O_2_CH_3_). HRMS (EI): *m*/*z* [M^+^] calcd. for C_9_H_6_N_2_O_2_Br_2_: 331.8796; found: 331.8792.
*Methyl (E)-3-(1-(2-methoxy-2-oxoethyl)-1H-pyrrol-2-yl)acrylate* (**10a**). At 0 °C and under N_2_ atmosphere, NaH (60%, 0.032 g, 0.80 mmol) was added to a solution of **9a** (0.100 g, 0.66 mmol) in anhydrous DMF (2.0 mL), and the mixture was stirred for 15 min. Then, **7a** (0.124 g, 0.80 mmol) was added, and the solution was stirred at rt for 3 h. The reaction mixture was extracted with a hexane/EtOAc (1:1, 15.0 mL), the organic layer was washed with brine (5.0 mL × 3) and dried (Na_2_SO_4_), and the solvent was removed under vacuum. The residue was purified by column chromatography over silica gel (hexane/EtOAc, 9:1), resulting in **10a** (0.088 g, 60%) as a white solid. R*_f_* 0.32 (hexane/EtOAc, 7:3); mp 116–118 °C. IR (film): ῡ 2960, 1753, 1688, 1623, 1467, 1294, 1171, 1080, 987, 745 cm^−1^. ^1^H NMR (600 MHz, CDCl_3_): δ 3.76 (s, 3H, =CHCO_2_C*H*_3_), 3.77 (s, 3H, CH_2_CO_2_C*H*_3_), 4.75 (s, 2H, C*H*_2_), 6.15 (d, *J* = 15.6 Hz, 1H, H-2), 6.24–6.26 (m, 1H, H-4′), 6.72 (dd, *J* = 3.8, 1.4 Hz, 1H, H-3′), 6.78 (br t, *J* = 1.4 Hz, 1H, H-5′), 7.43 (d, *J* = 15.6 Hz, 1H, H-3). ^13^C NMR (150 MHz, CDCl_3_): δ 48.2 (*C*H_2_), 51.5 (CH_2_CO_2_*C*H_3_), 52.7 (=CHCO_2_*C*H_3_), 110.4 (C-4′), 112.3 (C-3′), 113.5 (C-2), 126.8 (C-5′), 129.4 (C-2′), 131.5 (C-3), 167.9 (=CH*C*O_2_CH_3_), 168.4 (CH_2_*C*O_2_CH_3_). HRMS (EI): *m*/*z* [M^+^] calcd. for C_11_H_13_NO_4_: 223.0845; found: 223.0845.
*Methyl (E)-3-(5-formyl-1-(2-methoxy-2-oxoethyl)-1H-pyrrol-2-yl)acrylate* (**8i**). At 0 °C and under N_2_ atmosphere, POCl_3_ (0.220 g, 1.44 mmol) was added to DMF (0.105 g, 1.44 mmol), and the mixture was stirred for 10 min. Subsequently, **10a** (0.200 g, 0.90 mmol) in anhydrous CH_2_Cl_2_ (6.0 mL) was added dropwise, and the solution was stirred at 0 °C for 3 h. The reaction mixture was quenched with a 2N aqueous solution of NaOH until pH 8.0, and then CH_2_Cl_2_ (21.0 mL) was added. The mixture was washed with brine (7.0 mL × 3), the organic layer was dried (Na_2_SO_4_), and the solvent was removed under vacuum. The residue was purified by column chromatography over silica gel (hexane/EtOAc, 9:1) to furnish **8i** (0.142 g, 63%) as a pale brown solid. R*_f_* 0.67 (hexane/EtOAc, 1:1); mp 108–110 °C. IR (film): ῡ 2960, 1741, 1704, 1663, 1198, 1168, 980, 778 cm^−1^. ^1^H NMR (500 MHz, CDCl_3_): δ 3.75 (s, 3H, CH_2_CO_2_C*H*_3_), 3.77 (s, 3H, =CHCO_2_C*H*_3_), 5.24 (s, 2H, C*H*_2_), 6.41 (d, *J* = 15.8 Hz, 1H, H-2), 6.70 (d, *J* = 4.0 Hz, 1H, H-3′), 6.97 (d, *J* = 4.0 Hz, 1H, H-4′), 7.41 (d, *J* = 15.8 Hz, 1H, H-3), 9.53 (C*H*O). ^13^C NMR (125 MHz, CDCl_3_) δ 46.3 (*C*H_2_), 52.0 (CO_2_*C*H_3_), 52.8 (CO_2_*C*H_3_), 111.4 (C-3′), 121.1 (C-2), 124.6 (C-4′), 129.7 (C-3), 133.7 (C-5′), 137.7 (C-2′), 166.7 (=CH*C*O_2_CH_3_), 168.3 (CH_2_*C*O_2_CH_3_), 180.2 (*C*HO). HRMS (EI): *m*/*z* [M^+^] calcd. for C_12_H_13_NO_5_: 251.0794; found: 251.0785.
*Methyl (Z)-3-(dimethylamino)-2-(2-formyl-5-((E)-3-methoxy-3-oxoprop-1-en-1-yl)-1H-pyrrol-1-yl)acrylate* (**11**). Following the method described for **1b**, a mixture of **8i** (0.090 g, 0.36 mmol) and DMFDMA (0.213 g, 1.79 mmol) in anhydrous DMF (1.0 mL) afforded **11** (0.031 g, 28%) as a pale violet oil. R*_f_* 0.16 (hexane/EtOAc, 1:1). IR (film): ῡ 2957, 1619, 1435, 1164, 1103, 1037, 762 cm^−1^. ^1^H NMR (750 MHz, CDCl_3_): δ 2.46 (br, 6H, N(CH_3_)_2_), 3.63 (s, 3H, CO_2_C*H*_3_-1), 3.78 (s, 3H, =CHCO_2_C*H*_3_), 6.41 (d, *J =* 16.1 Hz, 1H, H-2″), 6.74 (d, *J* = 3.8 Hz, 1H, H-4′), 7.05 (d, *J* = 3.8 Hz, 1H, H-3′) 7.41 (d, *J* = 16.1 Hz, 1H, H-1″), 7.67 (s, 1H, H-3), 9.61 (s, 1H, C*H*O). ^13^C NMR (187.5 MHz, CDCl_3_): δ 36.4 (br, N(*C*H_3_)_2_), 51.6 (CO_2_*C*H_3_-1), 51.8 (=CHCO_2_*C*H_3_), 93.4 (C-2), 111.2 (C-4′), 119.5 (C-2″), 120.6 (C-3′), 131.6 (C-1″), 136.8 (C-2′), 139.0 (C-5′), 147.4 (C-3), 166.8 (*C*O_2_CH_3_-1), 167.0 (=CH*C*O_2_CH_3_), 180.0 (*C*HO). HRMS (EI): *m*/*z* [M^+^] calcd. for C_15_H_18_N_2_O_5_: 306.1216; found: 306.1214.
*Methyl (E)-6-(3-methoxy-3-oxoprop-1-en-1-yl)pyrrolo[1,2-a]pyrazine-4-carboxylate* (**4l**). Following method A described for **4a**, a mixture of **11** (0.030 g, 0.10 mmol), NH_4_OAc (0.023 g, 0.30 mmol), and Li_2_CO_3_ (0.022 g, 0.30 mmol) was heated at 70 °C for 7 h to give **4l** (0.015 g, 61%) as a yellow solid. *R_f_* 0.25 (hexane/EtOAc, 1:1); mp 100–102 °C. IR (film): ῡ 2917, 2852, 1717, 1623, 1717, 1623, 1427, 1330, 1262, 1171, 1099, 1066 cm^−1^. ^1^H NMR (600 MHz, CDCl_3_): δ 3.82 (s, 3H, =CHCO_2_C*H*_3_), 4.04 (s, 3H, CO_2_C*H*_3_-4), 6.39 (d, *J* = 15.6 Hz, 1H, H-2′), 7.18 (d, *J* = 4.2 Hz, 1H, H-8), 7.35 (d, *J* = 4.2 Hz, 1H, H-7), 7.70 (d, *J* = 15.6 Hz, 1H, H-1′), 8.24 (s, 1H, H-3), 8.93 (br s, 1H, H-1). ^13^C NMR (187.5 MHz, CDCl_3_): δ 51.8 (=CHCO_2_*C*H_3_), 53.0 (CO_2_*C*H_3_-4), 108.5 (C-8), 115.9 (C-2′), 118.1 (C-7), 120.9 (C-4), 126.9 (C-6), 132.01 (C-8a), 133.3 (C-1′), 135.2 (C-3), 147.9 (C-1), 163.3 (*C*O_2_CH_3_-4), 167.1 (=CH*C*O_2_CH_3_). HRMS (EI): *m*/*z* [M^+^] calcd. for C_13_H_12_N_2_O_4_: 260.0797; found: 260.0795.
*Ethyl (E)-3-(1-(2-methoxy-2-oxoethyl)-1H-pyrrol-2-yl)acrylate* (**10c**). Following the method described for **10a**, a mixture of **9b** (0.100 g, 0.60 mmol), NaH (60%, 0.029 g, 0.72 mmol), and **7a** (0.110 g, 0.72 mmol) formed **10c** (0.10 g, 70%) as a pale brown solid. R*_f_* 0.67 (hexane/EtOAc, 1:1); mp 56–57 °C. IR (film): ῡ 2997, 2949, 1742, 1692, 1626, 1474, 1290, 1171, 1080, 983, 745 cm^−1^. ^1^H NMR (400 MHz, CDCl_3_): δ 1.31 (t, *J* = 7.2 Hz, 3H, CO_2_CH_2_C*H*_3_), 3.77 (s, 3H, CO_2_C*H*_3_), 4.22 (q, *J* = 7.2 Hz, 2H, CO_2_C*H*_2_CH_3_), 4.75 (s, 2H, C*H*_2_), 6.16 (d, *J* = 15.6 Hz, 1H, H-2), 6.26–6.26 (ddd, *J* = 4.0, 3.0, 0.8 Hz, 1H, H-4′), 6.71 (dd, *J* = 4.0, 1.4 Hz, 1H, H-3′), 6.78 (dd, *J* = 3.0, 1.4 Hz, 1H, H-5′), 7.44 (d, *J* = 15.6 Hz, 1H, H-3). ^13^C NMR (100 MHz, CDCl_3_): δ 14.3 (CO_2_CH_2_*C*H_3_), 48.2 (*C*H_2_), 52.7 (CO_2_*C*H_3_), 60.2 (CO_2_*C*H_2_CH_3_), 110.4 (C-4′), 112.1 (C-3′), 114.0 (C-2), 126.7 (C-5′), 129.4 (C-2′), 131.3 (C-3), 167.5 (*C*O_2_CH_2_CH_3_), 168.4 (*C*O_2_CH_3_). HRMS (EI): *m*/*z* [M^+^] calcd. for C_12_H_15_NO_4_: 237.1001; found: 237.1001.
*Methyl (Z)-3-(dimethylamino)-2-(2-((E)-3-methoxy-3-oxoprop-1-en-1-yl)-1H-pyrrol-1-yl)acrylate* (**12a**). Following the method described for **1b**, a mixture of **10a** (0.119 g, 0.53 mmol) and DMFDMA (0.318 g, 2.67 mmol) in anhydrous DMF (1.0 mL) provided **12a** (0.134 g, 90%) as a pale violet oil. R*_f_* 0.33 (hexane/EtOAc, 7:3). IR (film): ῡ 2949, 1690, 1615, 1308, 1208, 1164, 1103, 1079, 1034 cm^−1^. ^1^H NMR (400 MHz, CDCl_3_): δ 2.70 (br, 6H, N(C*H*_3_)_2_), 3.62 (s, 3H, =CHCO_2_C*H*_3_), 3.73 (s, 3H, CO_2_C*H*_3_-1), 6.15 (d, *J* = 15.8 Hz, 1H, H-2″), 6.23 (ddd, *J* = 3.7, 2.4, 0.4 Hz, 1H, H-4′), 6.78 (dd, *J* = 3.7, 1.5 Hz, 1H, H-3′), 6.71 (dd, *J* = 2.4, 1.5 Hz, H-5′), 7.37 (d, *J* = 15.8 Hz, 1H, H-1″), 7.57 (s, 1H, H-3). ^13^C NMR (100 MHz, CDCl_3_): δ 35.2 (N(*C*H_3_)_2_), 46.7 (N(*C*H_3_)_2_), 51.3 (CO_2_*C*H_3_-1), 51.5 (=CHCO_2_*C*H_3_), 96.3 (C-2), 110.1 (C-4′), 111.7 (C-3′), 112.5 (C-2″), 130.1 (C-5′), 132.3 (C-2′), 133.6 (C-1″), 147.1 (C-3), 167.6 (*C*O_2_CH_3_-1), 168.2 (=CH*C*O_2_CH_3_). HRMS (EI): *m*/*z* [M^+^] calcd. for C_14_H_18_N_2_O_4_: 278.1267; found: 278.1263.
*Ethyl (Z)-3-(dimethylamino)-2-(2-((E)-3-ethoxy-3-oxoprop-1-en-1-yl)-1H-pyrrol-1-yl)acrylate* (**12b**). In a MW glass vial equipped with a magnetic stirring bar and sealed with a cap, a mixture of **10b** (0.300 g, 1.20 mmol) and *tert*-butoxy bis(dimethylamino)methane (0.625 g, 3.60 mmol) was heated at 125 °C for 2.0 h under MW irradiation (200 W) and a N_2_ atmosphere. The crude mixture was suspended and stirred in CH_2_Cl_2_/toluene (10:1, 11 mL), and the solvent was removed under vacuum. The residue was purified by column chromatography over silica gel (hexane/EtOAc, 7:3), resulting in **12b** (0.150 g, 41%) as a pale brown oil. R*_f_* 0.52 (hexane/EtOAc, 1:1). IR (film): ῡ 2977, 1690, 1615, 1301, 1212, 1161, 1079, 1034 cm^−1^. ^1^H NMR (500 MHz, CDCl_3_): δ 1.16 (t, *J* = 7.0 Hz, 3H, CO_2_CH_2_C*H*_3_), 1.29 (t, *J* = 7.0 Hz, 3H, CO_2_CH_2_C*H*_3_), 2.29 (br, 3H, N(C*H*_3_)_2_), 3.01 (br, 3H, N(C*H*_3_)_2_), 4.05–4.17 (m, 2H, CO_2_C*H*_2_CH_3_), 4.17–4.23 (m, 2H, CO_2_C*H*_2_CH_3_), 6.15 (d, *J* = 15.5 Hz, 1H, H-2″), 6.23 (dd, *J* = 4.0, 2.5 Hz, 1H, H-4′), 6.67 (dd, *J* = 4.0, 1.5 Hz, 1H, H-3′), 6.71 (dd, *J* = 2.5, 1.5 Hz, 1H, H-5′), 7.37 (d, *J* = 15.5 Hz, 1H, H-1″), 7.56 (s, 1H, H-3). ^13^C NMR (125 MHz, CDCl_3_): δ 14.4 (CO_2_CH_2_*C*H_3_), 14.5 (CO_2_CH_2_*C*H_3_), 38.6 (N(*C*H_3_)_2_), 59.98 (CO_2_*C*H_2_CH_3_), 60.03 (CO_2_*C*H_2_CH_3_), 96.3 (C-2), 110.0 (C-4′), 111.6 (C-3′), 112.7 (C-2″), 130.1 (C-5′), 132.4 (C-2′), 133.4 (C-1″), 146.9 (C-3), 167.2 (*C*O_2_CH_2_CH_3_-1), 168.9 (=CH*C*O_2_CH_3_). HRMS (EI): *m*/*z* [M^+^] calcd. for C_16_H_22_N_2_O_4_: 306.1580; found: 306.1573.
*Methyl (Z)-3-(dimethylamino)-2-(2-((E)-3-ethoxy-3-oxoprop-1-en-1-yl)-1H-pyrrol-1-yl)acrylate* (**12c**). Following the method described for **1b**, a mixture of **10c** (0.060 g, 0.25 mmol) and DMFDMA (0.151 g, 1.25 mmol) in anhydrous DMF (2.0 mL) afforded **12c** (0.068 g, 92%) as a pale violet oil. R*_f_* 0.48 (hexane/EtOAc, 1:1). IR (film): ῡ 2923, 1697, 1618, 1308, 1212, 1158, 1079, 1308, 1034 cm^−1^. ^1^H NMR (500 MHz, CDCl_3_): δ 1.29 (t, *J* = 7.2 Hz, 3H, CO_2_CH_2_C*H*_3_), 2.26 (br, 3H, N(C*H*_3_)_2_), 3.05 (br, 3H, N(C*H*_3_)_2_), 3.63 (s, 3H, CO_2_C*H*_3_), 4.20 (q, *J* = 7.2 Hz, 2H, CO_2_C*H*_2_CH_3_), 6.15 (d, *J* = 16.0 Hz, 1H, H-2″), 6.24 (br d, *J* = 3.3 Hz, 1H, H-4′), 6.68 (br d, *J* = 3.3 Hz, 1H, H-3′), 6.71 (br t, *J* = 1.7 Hz, 1H, H-5′), 7.38 (d, *J* = 16.0 Hz, 1H, H-1″), 7.57 (s, 1H, H-3). ^13^C NMR (125 MHz, CDCl_3_): δ 14.4 (CO_2_CH_2_*C*H_3_), 35.0 (N(*C*H_3_)_2_), 47.4 (N(*C*H_3_)_2_), 51.5 (CO_2_*C*H_3_), 60.1 (CO_2_*C*H_2_CH_3_), 96.3 (C-2), 110.1 (C-4′), 111.6 (C-3′), 113.0 (C-2″), 130.1 (C-5′), 132.4 (C-2′), 133.3 (C-1″), 147.1 (C-3), 167.7 (*C*O_2_CH_3_), 167.8 (*C*O_2_CH_2_*C*H_3_). HRMS (EI): *m*/*z* [M^+^] calcd. for C_15_H_20_N_2_O_4_: 292.1423; found: 292.1414.
*Dimethyl indolizine-5,7-dicarboxylate* (**5a**). At rt and under N_2_ atmosphere, a solution of AlCl_3_ (0.043 g, 0.321 mmol) in nitrobenzene (1.0 M) was added to a solution of **12a** (0.030 g, 0.17 mmol) in anhydrous CH_2_Cl_2_ (5.0 mL). The mixture was stirred at rt for 2 h before adding CH_2_Cl_2_ (15 mL). It was then washed with brine (5.0 mL × 3) and dried (Na_2_SO_4_), and the solvent was removed under vacuum. The residue was purified by column chromatography over silica gel (hexane/EtOAc, 9:1) to give **5a** (0.024 g, 66%) as a yellow solid. R*_f_* 0.80 (hexane/EtOAc, 1:1); mp 110–112 °C. IR (film): ῡ 2919, 1707, 1625, 1434, 1232, 1195, 1161, 1082, 758, 730 cm^−1^. ^1^H NMR (750 MHz, CDCl_3_): δ 3.94 (s, 3H, CO_2_C*H*_3_-5), 4.00 (s, 3H, CO_2_C*H*_3_-7), 6.95 (dd, *J* = 3.8, 0.9 Hz, 1H, H-1), 7.02 (dd, *J* = 3.8, 1.9 Hz, 1H, H-2), 8.17 (br d, *J* = 1.5 Hz, 1H, H-6), 8.41 (br d, *J* = 1.5 Hz, 1H, H-8), 8.84 (br s, 1H, H-3). ^13^C NMR (187.5 MHz, CDCl_3_): δ 52.2 (CO_2_*C*H_3_-7), 52.4 (CO_2_*C*H_3_-5), 107.6 (C-1), 116.0 (C-5), 116.7 (C-2), 118.0 (C-6), 119.4 (C-3), 123.0 (C-7), 127.2 (C-8), 133.5 (C-8a), 163.1 (*C*O_2_CH_3_-5), 165.9 (*C*O_2_CH_3_-7). HRMS (EI): *m*/*z* [M^+^] calcd. for C_12_H_11_NO_4_: 233.0688; found: 233.0687.
*Diethyl indolizine-5,7-dicarboxylate* (**5b**). Following the method described for **5a**, a mixture of **12b** (0.060 g, 0.20 mmol) and AlCl_3_ (0.078 g, 0.39 mmol) in anhydrous CH_2_Cl_2_ (2.0 mL) was stirred at rt for 2h. After the further addition of AlCl_3_ (0.078 g, 0.39 mmol), the mixture was stirred for 2 h to obtain **5b** (0.016 g, 31%) as a yellow solid. R*_f_* 0.81 (hexane/EtOAc, 7:3); mp 55–57 °C. IR (film): ῡ 2923, 2851, 1707, 1226, 1198, 1182, 1024 cm^−1^. ^1^H NMR (500 MHz, CDCl_3_): δ 1.42 (t, *J* = 7.0 Hz, 3H, CO_2_CH_2_C*H*_3_), 1.45 (t, *J* = 7.0 Hz, 3H, CO_2_CH_2_C*H*_3_), 4.39 (q, *J* = 7.0 Hz, 2H, CO_2_C*H*_2_CH_3_), 4.46 (q, *J* = 7.0 Hz, 2H, CO_2_C*H*_2_CH_3_), 6.94 (br d, *J* = 4.0 Hz, 1H, H-1), 7.02 (dd, *J* = 4.0, 3.0 Hz, 1H, H-2), 8.17 (d, *J* = 1.5 Hz, 1H, H-6), 8.41 (d, *J* = 1.5 Hz, 1H, H-8), 8.85 (br s, 1H, H-3). ^13^C NMR (125 MHz, CDCl_3_): δ 14.33 (CO_2_CH_2_*C*H_3_), 14.41 (CO_2_CH_2_*C*H_3_), 61.1 (CO_2_*C*H_2_CH_3_), 61.6 (CO_2_*C*H_2_CH_3_), 107.4 (C-1), 116.4 (C-5), 116.6 (C-2), 117.8 (C-6), 119.4 (C-3), 123.2 (C-7), 127.0 (C-8), 133.5 (C-8a), 162.7 (*C*O_2_Et), 165.6 (*C*O_2_Et). HRMS (EI): *m*/*z* [M^+^] calcd. for C_12_H_11_NO_4_: 233.0688; found: 233.0687.
*7-Ethyl 5-methyl indolizine-5,7-dicarboxylate* (**5c**). Following the method described for **5a**, a mixture of **12c** (0.030 g, 0.10 mmol) and AlCl_3_ (0.028 g, 0.20 mmol) in anhydrous CH_2_Cl_2_ (2.0 mL) furnished **5c** (0.013 g, 52%) as a yellow solid. R*_f_* 0.87 (hexane/EtOAc, 1:1); mp 133–135 °C. IR (film): ῡ 2987, 1704, 1256, 1232, 1198, 1168, 1018, 751 cm^−1^. ^1^H NMR (600 MHz, CDCl_3_): δ 1.42 (t, *J* = 7.2 Hz, 3H, CO_2_CH_2_C*H*_3_), 4.00 (s, 3H, CO_2_C*H*_3_), 4.40 (q, *J* = 7.2 Hz, 2H, CO_2_C*H*_2_CH_3_), 6.94 (br d, *J* = 4.2 Hz, 1H, H-1), 7.02 (dd, *J* = 4.2, 2.0 Hz, 1H, H-2), 8.16 (br d, *J* = 1.8 Hz, H-6), 8.41 (s, 1H, H-8), 8.84 (br s, 1H, H-3). ^13^C NMR (150 MHz, CDCl_3_): δ 14.4 (CO_2_CH_2_*C*H_3_), 52.4 (CO_2_*C*H_3_), 61.1 (CO_2_*C*H_2_CH_3_), 107.5 (C-1), 116.4 (C-5), 116.6 (C-2), 118.1 (C-6), 119.4 (C-3), 123.0 (C-7), 127.1 (C-8), 133.5 (C-8a), 163.1 (*C*O_2_CH_3_), 165.5 (CO_2_CH_2_*C*H_3_). HRMS (EI): *m*/*z* [M^+^] calcd. for C_13_H_13_NO_4_: 247.0845; found: 247.0840.

### 3.3. Evaluation of Antifungal Activity

The pyrrole derivatives herein synthesized were submitted to the CLSI M27-A3 microdilution method, and an evaluation of the antifungal sensitivity of the various concentrations was carried out against *Candida* spp. (*C. albicans* ATCC 10231, *C. glabrata* CBS138, *C. dubliniensis* CD36, *C. krusei* ATCC 14423, *C. auris* Monterrey, and *C. haemulonii* ENCB87) [67]. The compounds were used at 187–0.01 μg/mL. The references were fluconazole, simvastatin, and atorvastatin. The inoculum of *Candida* spp. was adjusted in a spectrophotometer to 620 nm. Subsequently, a 1:1000 dilution was made with RPMI medium. The 96-well microplates were inoculated with 75 µL of yeast suspension and 75 µL of the concentration of the compound to be tested. RPMI served as the sterility control, and DMSO in the absence of an antifungal agent as the growth control. The microplates were incubated at 37 °C for 24 h, and growth was quantified in a microplate spectrophotometer at 620 nm. The values of yeast growth are expressed as the average of three independent assays.

### 3.4. Docking Studies

The sequences of the HMGR enzyme from each *Candida* spp., including *Candida albicans* (CaHMGR), *C. glabrata* (CgHMGR), *C. dubliniensis* (CdHMGR), *C. krusei* (CkHMGR), *C. auris* (CauHMGR), and *C. haemulonii* (ChaHMGR), were downloaded from the NCBI database [68]. The HMGR sequences were processed by the homology modeling technique to generate 3D models on the Modeller program version 10.2 [69]. The crystallized structure of the catalytic portion of human HMG-CoA reductase with simvastatin (PDB: 1HW9), deposited in the protein data bank (PDB) [70], served as a template. These 3D models were validated with the Procheck program [71] before being utilized in the docking studies, which were run on Autodock4 with the series of enaminones, *N*-alkyl pyrroles, pyrrolo[1,2-*a*]pyrazines, 2-methyl acrylates, and indolizines. The 2D structure of each ligand was sketched in the chemical editor ChemDraw [72] and converted to 3D in mol2 format with the Open Babel GUI program [73]. The structures of the reference compounds (simvastatin and atorvastatin) were downloaded from ZINC 20 [74] and optimized with Gaussian 16W software [75]. The instruction files were prepared in AutoDock Tools [76], setting a grid box of 80 × 62 × 58 Å, centered at: X = 23.123, Y = 9.113, and Z = 1.802, with a grid spacing of 0.375 Å^3^. In Autodock, the Lamarckian Genetic Algorithm was chosen, considering 100 docking trials, a population size of 150, a maximum number of energy evaluations of 25,000, a maximum number of generations of 27,000, and a mutation rate of 0.02. After docking, the coupling with the lowest binding energy (expressed in kcal/mol) was selected and analyzed with Biovia Discovery Studio 2017 R2 software [77].

## 4. Conclusions

New synthetic strategies are described for the preparation of pyrrolo[1,2-*a*]pyrazines and indolizines. The design of these approaches is centered on the cyclization of pyrrole-based enaminone precursors through treatment with ammonium acetate to afford pyrrolo[1,2-*a*]pyrazines, and on the application of a Lewis acid to enaminone precursors to furnish indolizines. All the intermediates and precursors were synthesized starting from 2-formylpyrrole (**6**), following short and efficient routes. The stereoselective introduction of the (*Z*)-enaminone moiety was achieved by reacting the *N*-alkyl pyrrole with DMFDMA under conventional heating or with Bredereck’s reagent under MW irradiation. The following series of the intermediates and cyclization products were: enaminone-containing pyrroles **1a**, **1c**, and **2a**–**d**, pyrrolo[1,2-*a*]pyrazines **4a**–**l**, indolizines **5a**–**c**, *N*-alkyl 2-formylpyrroles **8a**–**h**, and pyrrole-based alkyl acrylates **8i**, **10a**–**c**, **11**, and **12a**–**c**, which were evaluated for their in vitro growth inhibition of six *Candida* spp. (*C. albicans*, *C. glabrata*, *C. dubliniensis*, *C. krusei, C. auris*, and *C. haemulonii*). The majority of these compounds demonstrated good inhibition of yeast growth. Docking simulations were carried out between the twelve most active compounds and the three most prevalent *Candida* spp. (*C. albicans*, *C. glabrata*, and *C. auris*). Insights were provided into the possible mechanism of action of the compounds evaluated. Polar and non-polar interactions between the functional groups of the derivatives and the active site of the HMGR enzyme of the three selected yeasts are involved, which have also been established for simvastatin and atorvastatin (the reference drugs). The lead compounds bear plausible pharmacophore groups responsible for the antifungal activity, which was found against all six *Candida* spp. analyzed, including the two multidrug-resistant species. Hence, the structural requirement is presently established in order that the most active analogues can be used in future studies on drug design and development.

## Figures and Tables

**Table 1:** Reaction conditions and yields for the preparation of 4-substituted pyrrolo[1,2-*a*]pyrazines **4a**–**g** ^a^.

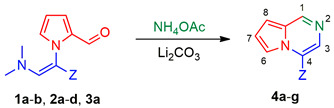					
Entry	1–3	Z	T (°C)	t (h)	4 (%) ^b^
1	**1a**	CO_2_Me	70	4	**4a** (90)
2	**1b**	CO_2_Et	80	3	**4b** (66)
3	**2a**	COPh	80	3	**4c** (87)
4	**2b**	COC_6_H_4_-3-OMe	70	4	**4d** (83)
5	**2c**	COC_6_H_4_-4-OMe	70	4	**4e** (84)
6	**2d**	COC_6_H_3_-3,4-(OMe)_2_	70	4	**4f** (60)
7	**3a**	CN	70	4	**4g** (53)

^a^ The reactions were carried out with the enaminones **1**–**3** (1.0 mol eq.), NH_4_OAc (3.0 mol eq.), and Li_2_CO_3_ (3.0 mol eq.) in anh. DMF. ^b^ After purification by column chromatography.

**Table 2:** MIC_50_ and MIC_70_ values for the inhibition of *Candida* spp. by the following compounds: enaminone-containing pyrroles **1a**, **1c**, and **2a**–**d**, pyrrolo[1,2-*a*]pyrazines **4a**–**l**, indolizines **5a**–**c**, *N*-alkyl 2-formylpyrroles **8a–h**, and pyrrole-based alkyl acrylates **8i**, **10a**–**c**, **11**, and **12a**–**c** ^a^.

Compound	*C. albicans*		*C. glabrata*		*C. dubliniensis*		*C. krusei*		*C. auris*		*C. haemulonii*	
	MIC_50_	MIC_70_	MIC_50_	MIC_70_	MIC_50_	MIC_70_	MIC_50_	MIC_70_	MIC_50_	MIC_70_	MIC_50_	MIC_70_
	µg/mL		µg/mL		µg/mL		µg/mL		µg/mL		µg/mL	
fluconazole	1.40	1.80	5.60	7.20	1.40	1.80	5.60	7.20	>44.8	>57.6	>44.8	>57.6
simvastatin	1.25	1.75	15.00	21.00	1.25	1.75	40.00	56.00	10.00	14.00	20.00	28.00
atorvastatin	3.77	5.27	1.71	2.39	2.77	3.87	4.80	6.72	15.3	21.42	8.00	11.20
**1a**	0.11	0.15	0.09	0.12	0.07	0.09	0.05	0.07	4.17	5.83	4.45	6.23
**1c**	4.20	5.88	0.65	0.91	4.00	5.60	0.36	0.50	2.59	3.62	2.00	2.80
**2a**	0.13	0.18	0.09	0.12	0.06	0.08	0.03	0.04	5.57	7.79	3.74	5.23
**2b**	0.13	0.18	0.11	0.15	0.07	0.09	0.05	0.07	5.31	7.43	5.20	7.28
**2c**	0.10	0.14	0.07	0.09	0.06	0.08	0.06	0.08	4.50	6.30	4.45	6.23
**2d**	0.12	0.16	0.12	0.16	0.09	0.12	0.04	0.06	4.68	6.55	12.48	17.47
**4a**	0.18	0.25	1.56	2.18	0.73	1.02	0.20	0.28	5.85	8.19	11.70	16.30
**4b**	0.15	0.21	0.11	0.15	0.07	0.09	0.09	0.12	3.07	4.29	3.90	5.46
**4c**	0.33	0.46	0.15	0.21	0.09	0.12	0.01	0.02	1.46	2.04	18.72	26.20
**4d**	0.45	0.63	0.12	0.16	0.16	0.22	0.18	0.36	1.62	2.25	11.70	16.38
**4e**	0.33	0.46	0.13	0.18	0.36	0.50	0.18	0.36	1.46	2.04	7.80	10.92
**4f**	0.18	0.25	0.16	0.22	0.09	0.12	0.36	0.50	10.63	14.88	11.70	16.38
**4g**	0.07	0.09	0.18	0.25	0.09	0.12	0.14	0.19	6.15	8.61	6.88	9.63
**4h**	0.23	0.32	0.12	0.16	0.09	0.12	0.12	0.16	2.20	3.08	4.92	6.88
**4i**	0.08	0.11	0.15	0.21	0.01	0.02	0.28	0.39	11.68	16.35	11.68	16.35
**4j**	0.22	0.30	0.11	0.15	0.22	0.04	0.05	0.07	11.68	16.35	11.68	16.35
**4k**	0.10	0.14	0.08	0.11	0.07	0.09	0.18	0.36	3.90	5.46	18.72	26.20
**4l**	0.04	0.05	0.11	0.15	0.06	0.08	0.04	0.06	5.57	7.79	4.92	6.88
**5a**	0.25	0.17	0.12	0.16	0.12	0.16	0.12	0.16	4.00	5.60	3.12	4.36
**5b**	0.50	0.70	1.82	2.54	1.58	2.21	4.00	5.60	3.10	4.34	2.55	3.57
**5c**	0.50	0.70	2.00	2.80	2.45	3.43	0.45	0.63	2.60	3.64	3.24	4.53
**8a**	0.10	0.14	0.24	0.33	0.03	0.04	0.06	0.08	1.46	2.04	7.20	10.08
**8b**	0.19	0.26	0.12	0.16	0.14	0.19	0.06	0.08	2.96	4.14	18.72	26.20
**8c**	0.07	0.09	0.10	0.14	0.07	0.09	0.04	0.06	8.35	11.69	11.70	16.38
**8d**	0.20	0.28	0.03	0.04	0.05	0.07	0.05	0.07	6.15	8.61	11.70	16.38
**8e**	0.28	0.39	0.09	0.12	0.06	0.08	0.04	0.06	9.00	12.60	10.63	14.88
**8f**	0.20	0.28	0.05	0.07	0.05	0.07	0.05	0.07	4.68	6.55	10.63	14.88
**8g**	0.12	0.16	0.08	0.11	0.16	0.22	0.04	0.06	5.57	7.79	16.71	23.39
**8h**	4.00	5.60	0.82	1.14	2.43	3.40	1.00	1.40	2.38	3.33	2.27	3.17
**8i**	0.14	0.19	0.45	0.63	0.32	0.44	0.20	0.28	6.88	9.63	10.63	14.88
**10a**	0.50	0.70	0.50	0.70	0.55	0.77	0.88	1.23	3.57	4.99	4.00	5.60
**10b**	0.58	0.81	0.26	0.36	4.00	5.60	4.66	6.52	2.82	3.94	2.38	3.33
**10c**	3.74	5.23	1.42	1.98	1.08	1.51	1.02	1.42	2.56	3.58	2.30	3.22
**11**	0.18	0.25	0.84	1.17	0.73	1.02	0.16	0.22	4.87	6.81	5.85	8.19
**12a**	0.10	0.14	0.13	0.18	0.10	0.70	0.14	0.19	4.00	5.60	2.00	2.80
**12b**	0.12	0.17	0.91	1.27	2.14	2.99	0.88	1.20	2.80	3.92	2.27	3.17
**12c**	1.77	2.47	0.85	1.19	2.38	3.33	1.30	1.82	2.85	3.99	2.70	3.78

^a^ MIC_50_ is the 50% inhibition of yeast growth. MIC_70_ is the 70% inhibition of yeast growth.

**Table 3:** Binding energy values (DG, kcal/mol) at the active site of the HMGR enzymes of six *Candida* species for simvastatin and atorvastatin and for the test compounds: enaminone-containing pyrroles **1a**, **2a**, and **2c**, pyrrolo[1,2-*a*]pyrazines **4b**, **4g**, and **4l**, indolizine **5a**, *N*-alkyl 2-formylpyrroles **8a**, **8c**, and **8g**, and pyrrole-based alkyl acrylates **10a** and **12a** ^a^.

Compound	HMGR Enzymes of *Candida* spp.					
	*C. albicans*	*C. glabrata*	*C. dubliniensis*	*C. krusei*	*C. auris*	*C. haemulonii*
simvastatin	−6.12	−6.30	−6.57	−6.06	−6.51	−6.18
atorvastatin	−3.82	−4.63	−4.64	−4.97	−2.14	−4.66
**1a**	−6.21	−5.26	−5.42	−6.55	−5.47	−5.34
**2a**	−7.19	−7.48	−8.00	−8.73	−8.34	−6.89
**2c**	−7.14	−6.34	−8.05	−6.88	−7.98	−7.35
**4b**	−6.41	−5.53	−5.74	−7.02	−5.69	−7.01
**4g**	−5.74	−5.18	−5.18	−6.07	−5.18	−6.22
**4l**	−7.70	−6.44	−6.48	−7.58	−7.31	−6.43
**5a**	−7.22	−6.65	−6.73	−7.04	−6.71	−5.57
**8a**	−5.33	−4.74	−4.49	−5.55	−5.55	−5.92
**8c**	−8.24	−6.77	−6.81	−8.32	−7.06	−7.49
**8g**	−5.21	−5.00	−4.56	−5.28	−4.93	−5.58
**10a**	−6.42	−5.88	−5.92	−6.52	−6.11	−4.93
**12a**	−7.09	−7.20	−7.15	−5.42	−6.70	−5.50

^a^ *C. albicans* (CaHMGR), *C. glabrata* (CgHMGR), *C. dubliniensis* (CdHMGR), *C. krusei* (CkHMGR), *C. auris* (CauHMGR), and *C. haemulonii* (ChaHMGR).

**Table 4:** Data on the interactions of enaminones **1a**, **2a**, and **2c**, pyrrolo[1,2-*a*]pyrazines **4b**, **4g**, and **4l**, indolizine **5a**, *N*-alkyl 2-formylpyrroles **8a**, **8c**, and **8g**, and pyrrole-based alkyl acrylates **10a** and **12a** at the active site of the HMGR enzyme of *C. albicans* (CaHMGR).

Compound	Residues of the Enzyme Interacting with the Ligand	Polar Interactions	Hydrophobic Interactions
simvastatin	Ala62, Thr95, Glu96, Gly97, Cys98, Arg127, Met192, Met194, Asn195, Asp227, Lys228, Gly302, Gln303, Asp304, Gln307, Gly343, Gly344	Asp227, Lys228, Asp304	-
atorvastatin	Thr95, Glu96, Gly97, Met192, Met194, Asn195, Asp227, Lys228, Asn292, His289, Gly302, Gln303, Asp304, Gln307, Gly342, Gly343, Gly344, Leu389	Asn195, Asp304	Glu96, Met192, Met194, Lys228, Asp304
**1a**	Ala62, Cys63, Thr95, Glu96, Gly97, Met192, Gly302, Gln303, Asp304, Pro305, Gly343, Gly344	Asp304	Cys63, Glu96
**2a**	Glu96, Leu99, Arg127, Met194, Asn195, Ser221, Asp227, Lys228, Lys272, Ala288, His289, Ans292, Leu389	Asp227, Lys228, Asn292	Glu96, Met194, His289, Leu389
**2c**	Thr95, Glu96, Met192, Met194, Asn195, Asp227, Lys228, Gln303, Asp304, Pro305, Gly344	Lys228	Met192, Met194
**4b**	Thr95, Glu96, Met192, Gly302, Gln303, Asp304, Gln307, Glu337, Val338, Gly339, Ile341, Gly342, Gly343, Gly344, Thr345	Gly339, Ile341, Gly344	Met192, Asp304, Gly342, Gly343
**4g**	Leu73, Ala93, Thr94, Thr95, Glu96, Thr295, Ala 296, Leu299, Gln303, Asp304, Pro305	Thr95	Ala296, Pro305
**4l**	Leu73, Ala93, Thr94, Thr95, Glu96, Leu99, Asn292, Thr295, Ala296, Leu299, Gly302, Gln303, Asp304, pro305, Gly344	Ala93, Thr94	Leu73, Thr94, Ala296, Leu299, Pro305
**5a**	Thr95, Glu96, Met192, Met194, Asn195, Gly302, Gln303, Asp304, Gly339, Ile341, Gly342, Gly343, Gly344, Thr345	Thr95, Asn195, Gly302, Gly343, Gly344	Met192, Gly339, Gly342, Thr345
**8a**	Thr95, Glu96, Met192, Gly302, Gln303, Asp304, Gln307, Glu337, Val338, Gly339, Ile341 Gly342, Gly343, Gly344, Thr345	Asp304, Thr95, Val338, Gly344	Met192
**8c**	Thr95, Glu96, Met192, Met194, Asn195, Lys228, Gly302, Gln303, Asp304, Gly339, Ile341 Gly342, Gly343, Gly344, Thr345	Thr95, Gly344	Glu96, Met192
**8g**	Thr94, Thr95, Ala93, Glu96, Leu99, Asn292, Thr295, Ala296, Gln303, Pro305	Thr94, Thr95, Thr295	Ala296, Pro305
**10a**	Leu73, Ala93, Thr94, Thr95, Glu96, Leu99, Asn292, Thr295, Ala296, Leu299, Gln303, Asp304, Pro305, Ala306	Asn292, Thr295, Asp304	Leu73, Thr94, Ala296, Leu299, Pro305
**12a**	Ala62, Thr95, Glu96, Gly97, Met192, Gly193, Met194, Asn195, Gly302, Gln303, Asp304, Ile341, Gly342, Gly343, Gly344	Ala62, Gly97, Ile341	Met192, Met194

## Data Availability

The data presented in this study are available in the Supplementary Materials.

## Supplementary Materials

The following supporting information can be downloaded at: https://www.mdpi.com/article/10.3390/molecules28207223/s1. Figures S1–S76: ^1^H and ^13^C NMR spectra of all synthesized compounds; Figure S77: Acquisition and evaluation of the models of the HMGRs of the *Candida* spp; Figures S78–S83: Ramachandran plots of HMGRs; Tables S1 and S2: Data of the interactions between the simvastatin, atorvastatin, and compounds **1a**, **2a**, **2c**, **4b**, **4g**, **4l**, **5a**, **8a**, **8c**, **8g**, **10a**, and **12a** at the active site of the enzyme HMGR of *C. glabrata* (CgHMGR) and *C. auris* (CauHMGR); Figures S84 and 85: Representation of the interactions between simvastatin, atorvastatin, **1a**, **2a**, **2c**, **4b**, **4g**, **4l**, **5a**, **8a**, **8c**, **8g**, **10a**, and **12a** at the active site of CgHMGR and CauHMGR, respectively.

## Sample Availability

Not available.
