# Supplementary material for: Pyrrole-Based Enaminones as Building Blocks for the Synthesis of Indolizines and Pyrrolo[1,2-a]pyrazines Showing Potent Antifungal Activity
Source: Molecules. 2023 Oct 23;28(20):7223. doi: 10.3390/molecules28207223 (PMC10608852; doi:10.3390/molecules28207223)
Supplement: Supplementary file 1 [file molecules-28-07223-s001.zip › molecules-2627547-supplementary.pdf]

SUPPLEMENTARY MATERIALS
CONTENT

1. ^{1}H and ^{13}C NMR spectra of the prepared compounds (Figures S1-S76) S2-S39

2. Acquisition and evaluation of the models of the HMGRs of *Candida* spp. (Figure S77) S40

3. Ramachandran plots of HMGRs (Figures S78-S83) S41-S46

4. Interactions between simvastatin, atorvastatin, and compounds **1a**, **2a**, **2c**, **4b**, **4g**, **4l**, **5a**, **8a**, **8c**, **8g**, **10a**, and **12a** at the active site of the enzyme HMGR of *C. glabrata* (CgHMGR) (Table S1) and *C. auris* (CauHMGR) (Table S2) S47-S48

5. The predicted binding mode of simvastatin, atorvastatin, **1a**, **2a**, **2c**, **4b**, **4g**, **4l**, **5a**, **8a**, **8c**, **8g**, **10a**, and **12a** at the active site of CgHMGR (Figure S84) S49-S53

6. The predicted binding mode of simvastatin, atorvastatin **1a**, **2a**, **2c**, **4b**, **4g**, **4l**, **5a**, **8a**, **8c**, **8g**, **10a**, and **12a** at the active site of CauHMGR (Figure S85) S54-S58

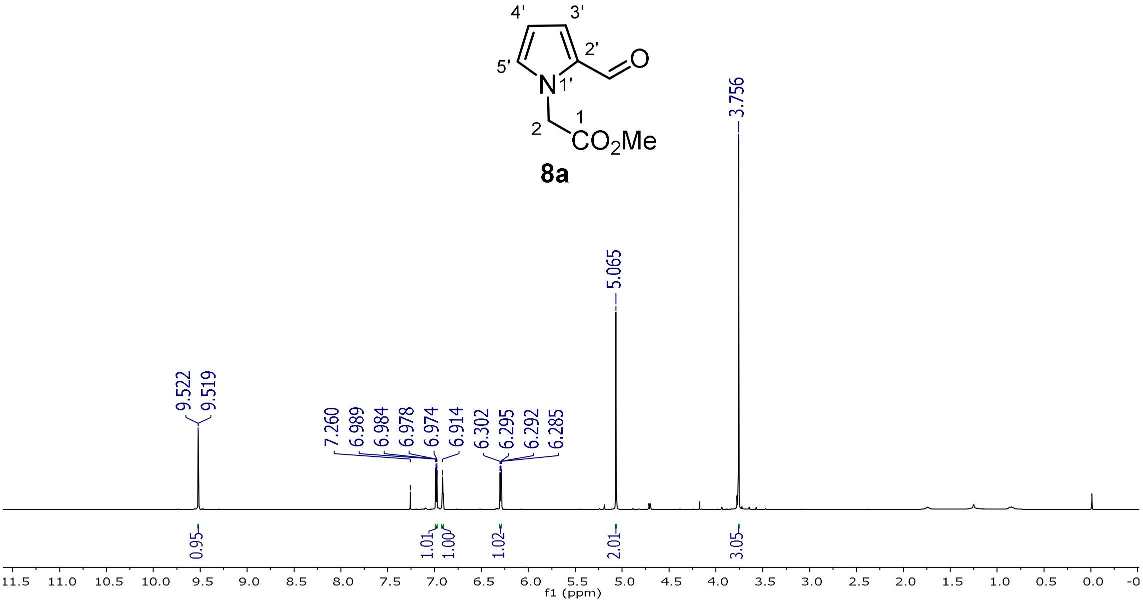

Figure S1. ^{1}H NMR (400 MHz, $CDCl_3$) spectrum of compound **8a**

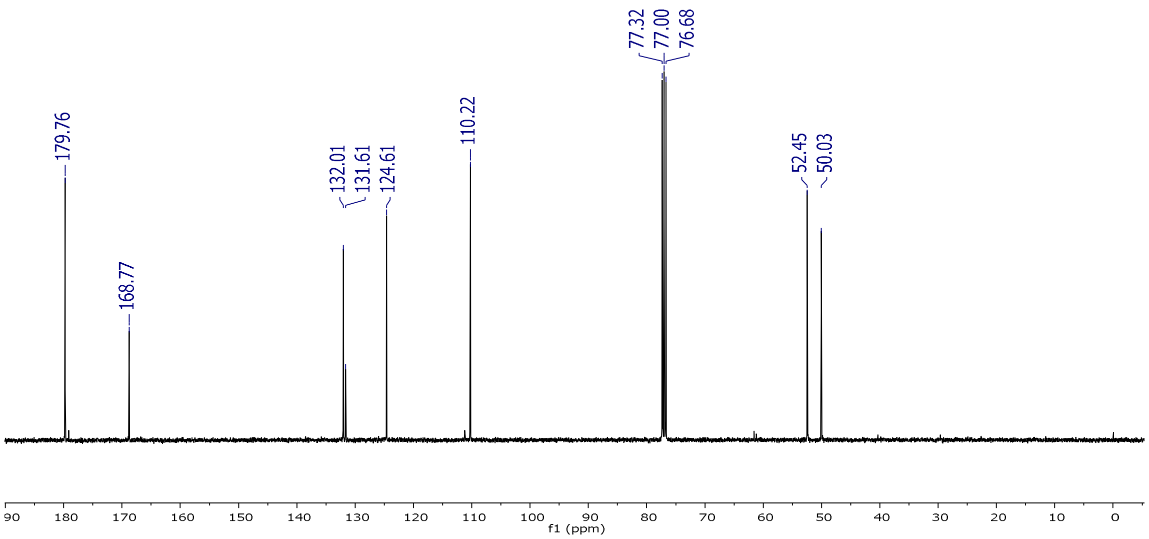

Figure S2. ^{13}C NMR (100 MHz, $CDCl_3$) spectrum of compound **8a**

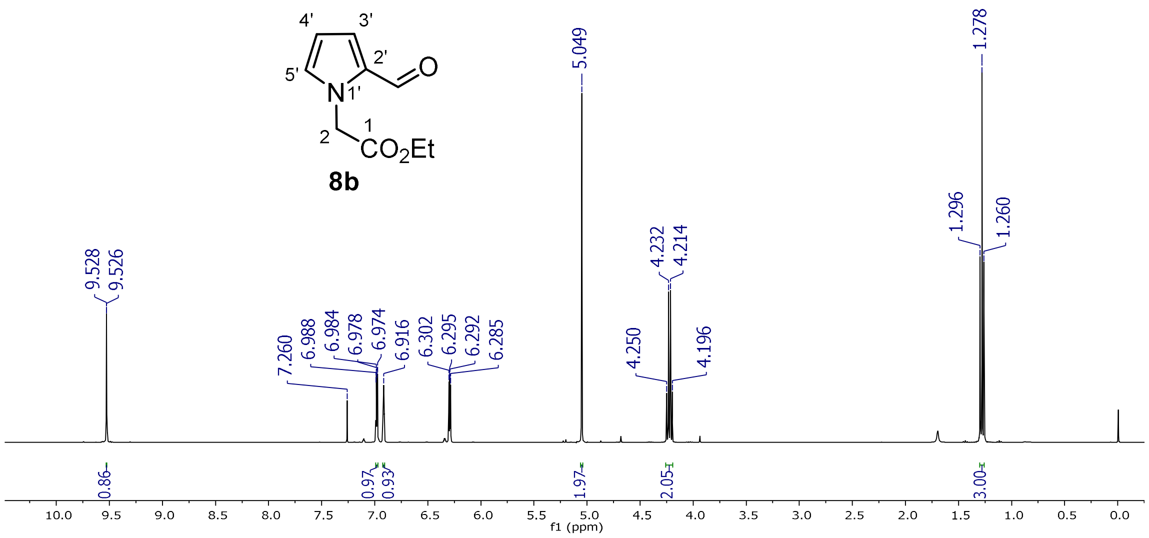

Figure S3. ^{1}H NMR (400 MHz, $CDCl_3$) spectrum of compound **8b**

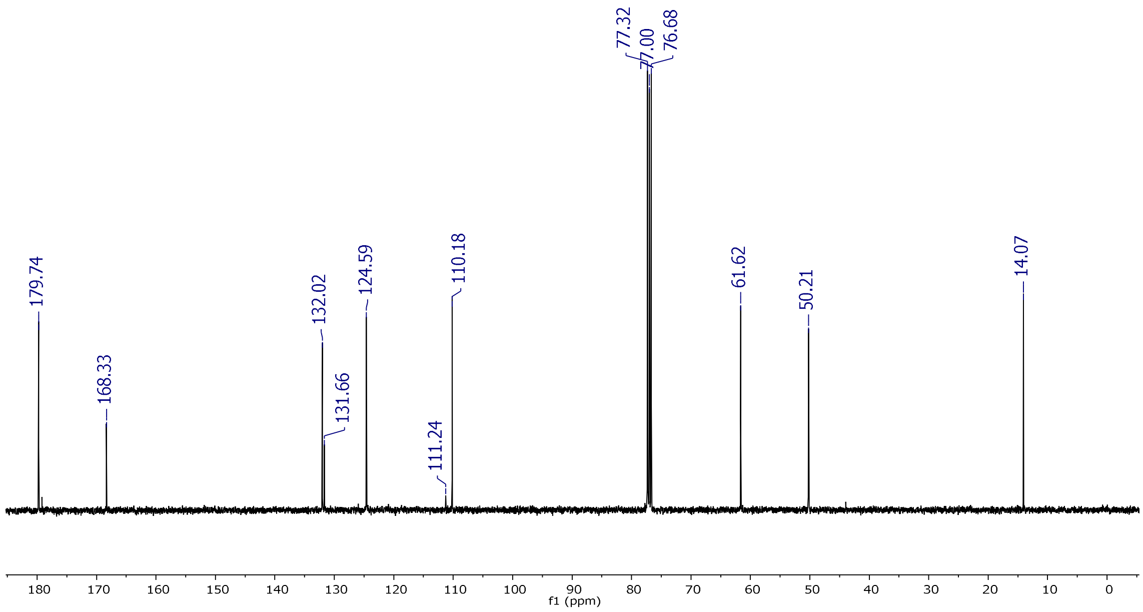

Figure S4. ^{13}C NMR (100 MHz, $CDCl_3$) spectrum of compound **8b**

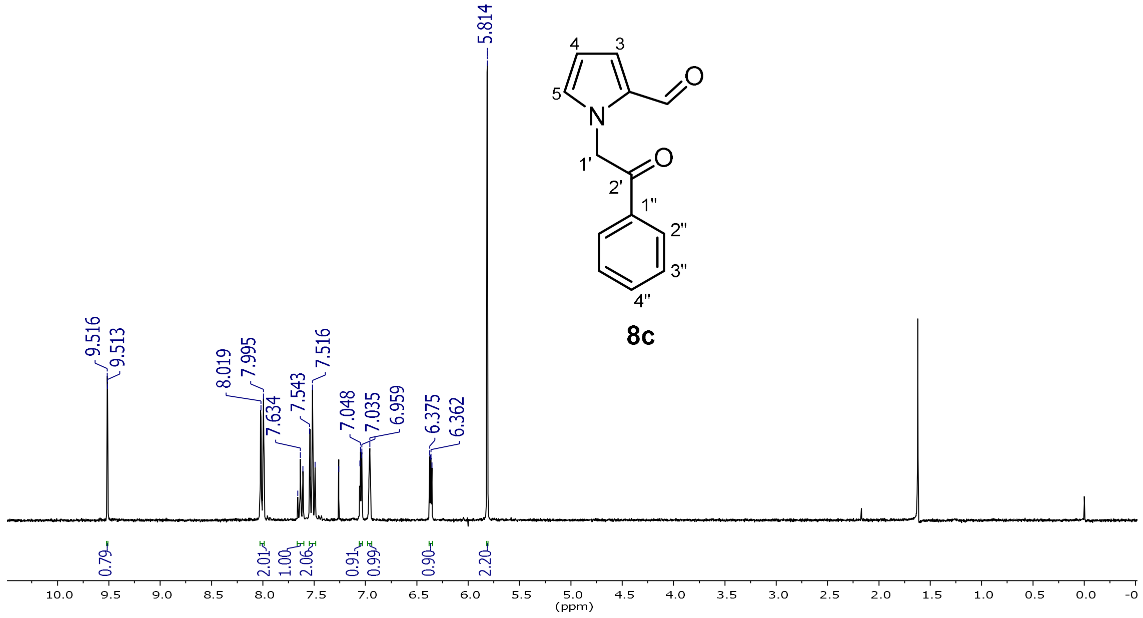

Figure S5. ^{1}H NMR (300 MHz, $CDCl_3$) spectrum of compound **8c**

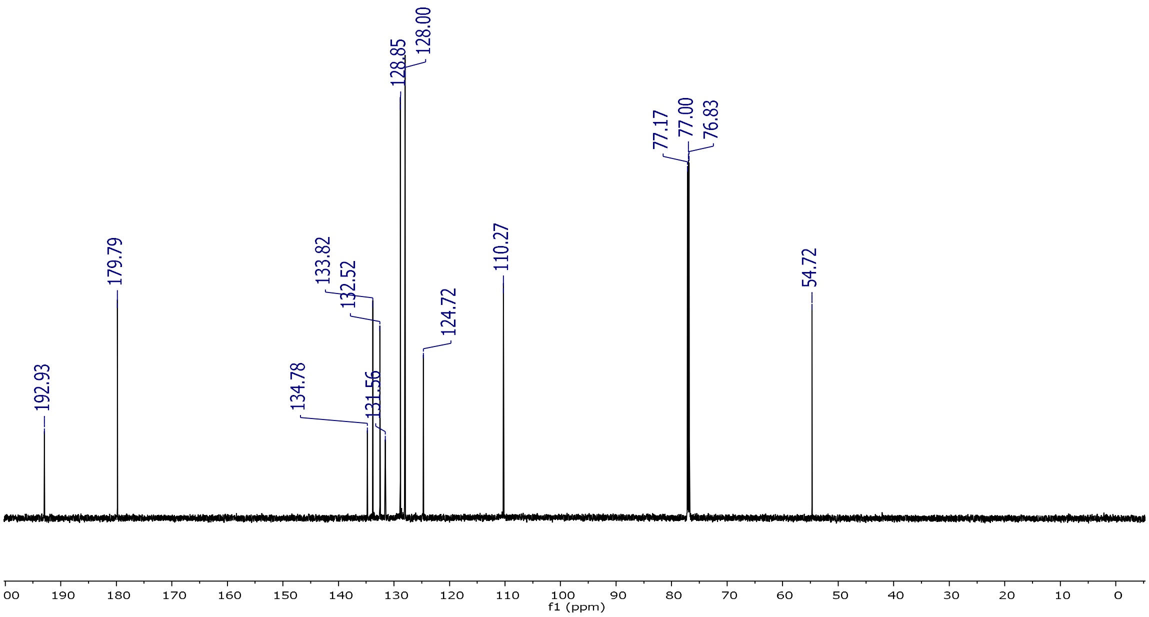

Figure S6. ^{13}C NMR (187.5 MHz, $CDCl_3$) spectrum of compound **8c**

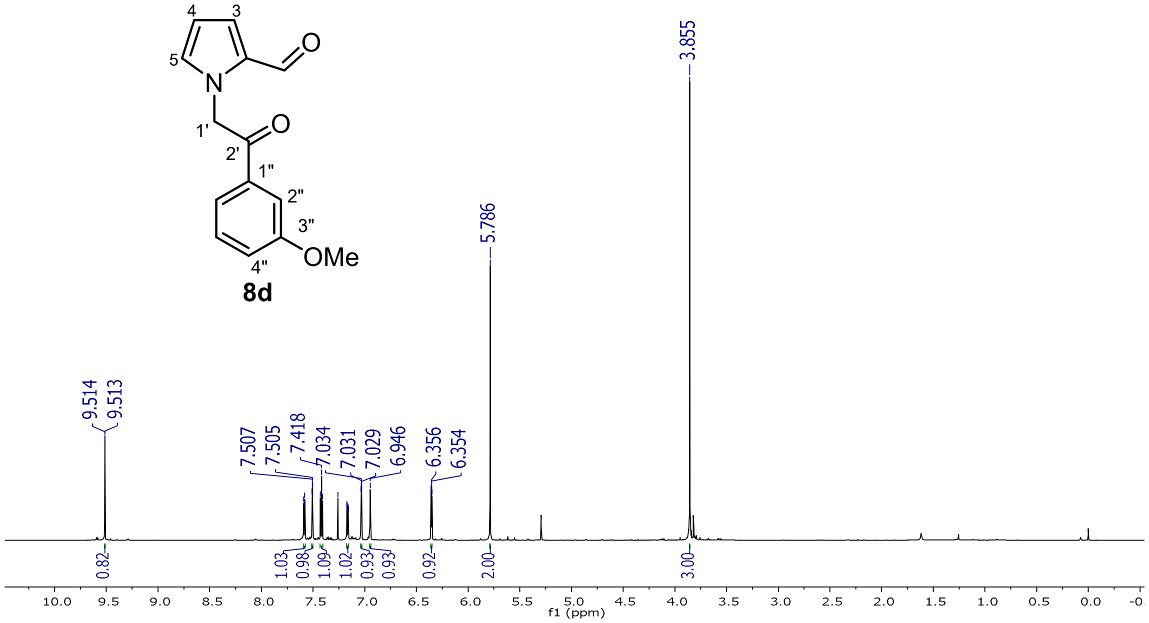

Figure S7. ^{1}H NMR (750 MHz, $CDCl_3$) spectrum of compound **8d**

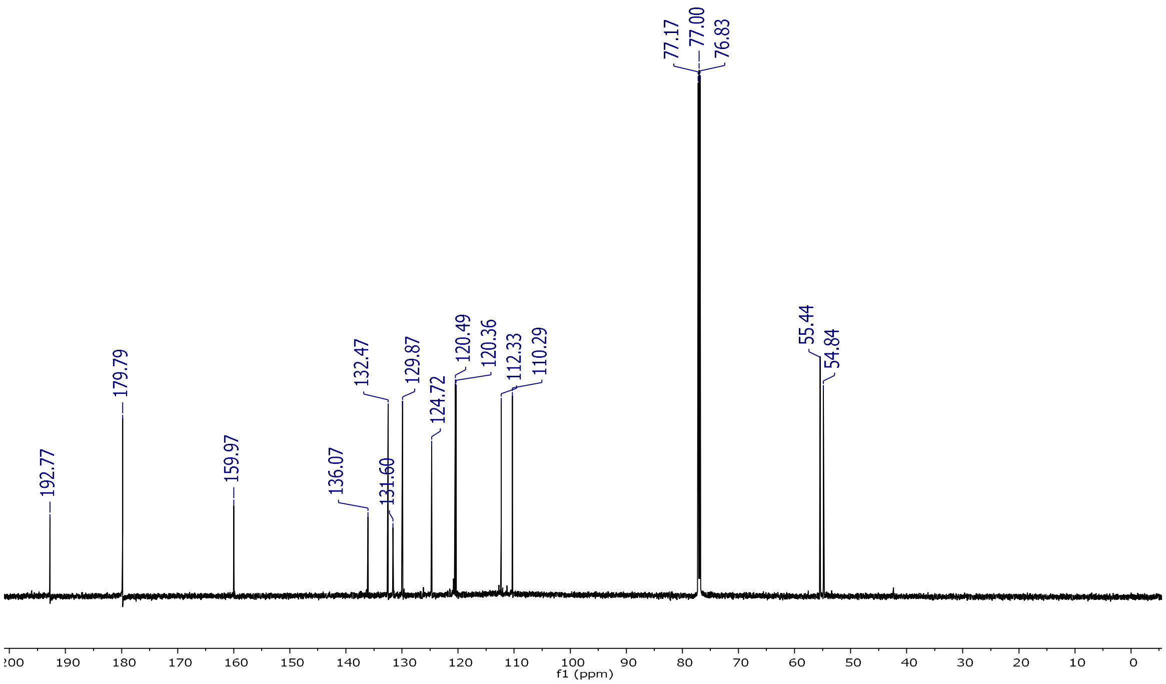

Figure S8. ^{13}C NMR (187.5 MHz, $CDCl_3$) spectrum of compound **8d**

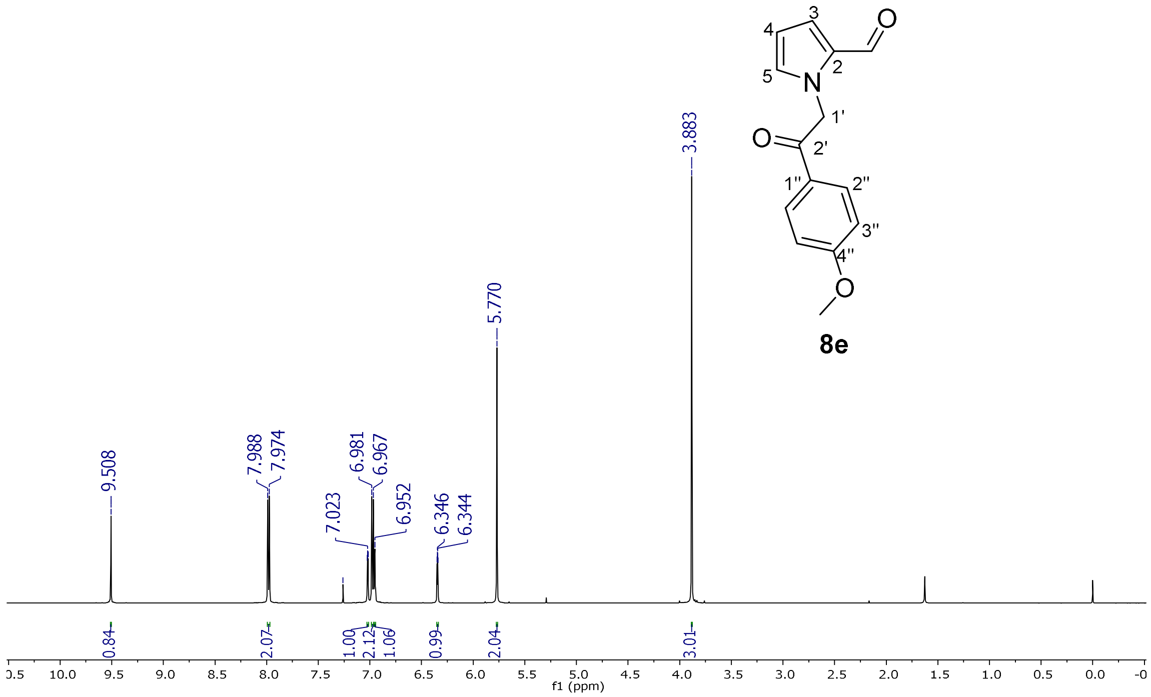

Figure S9. ^{1}H NMR (600 MHz, $CDCl_3$) spectrum of compound **8e**

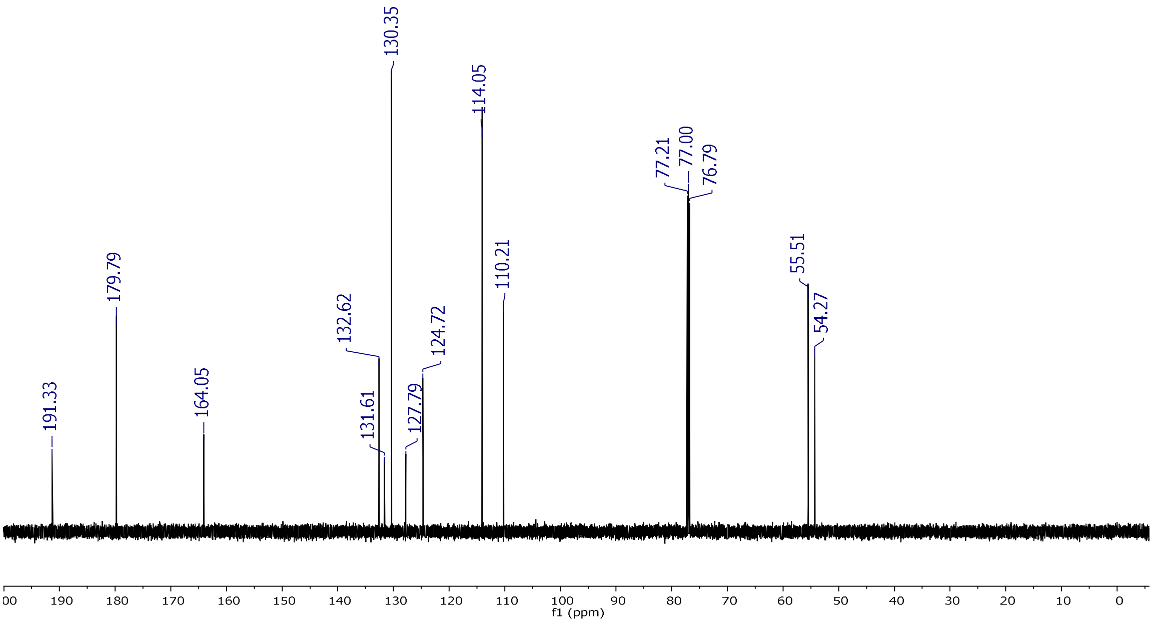

Figure S10. ^{13}C NMR (150 MHz, $CDCl_3$) spectrum of compound **8e**

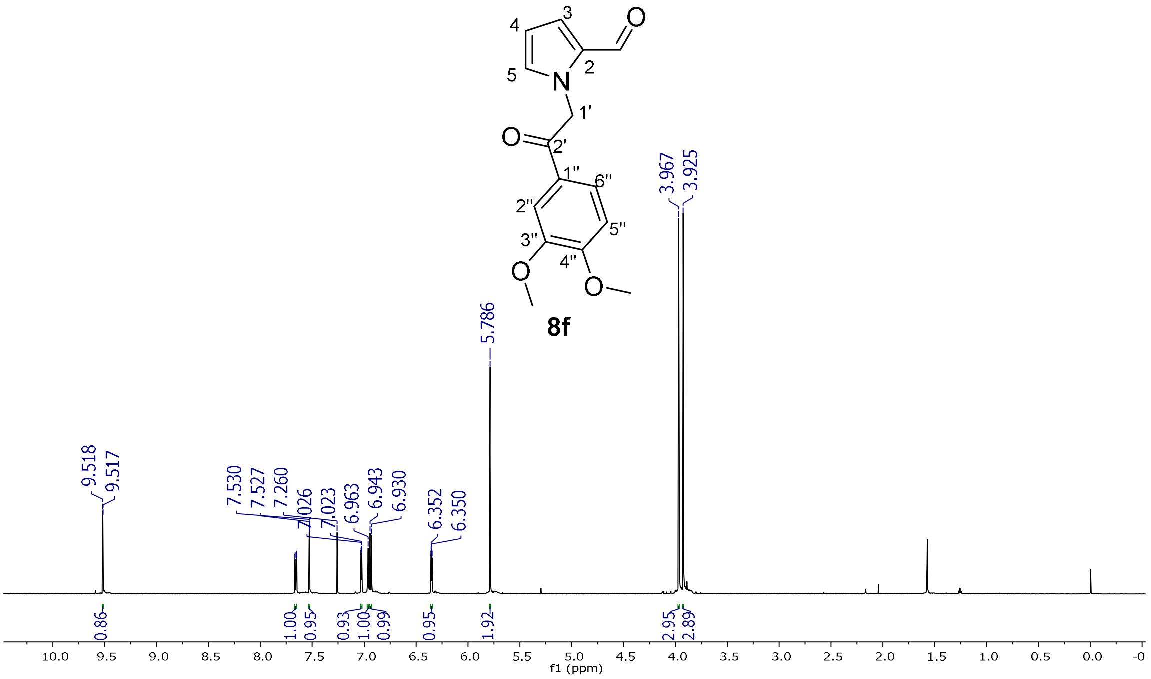

Figure S11. ^{1}H NMR (600 MHz, $CDCl_3$) spectrum of compound **8f**

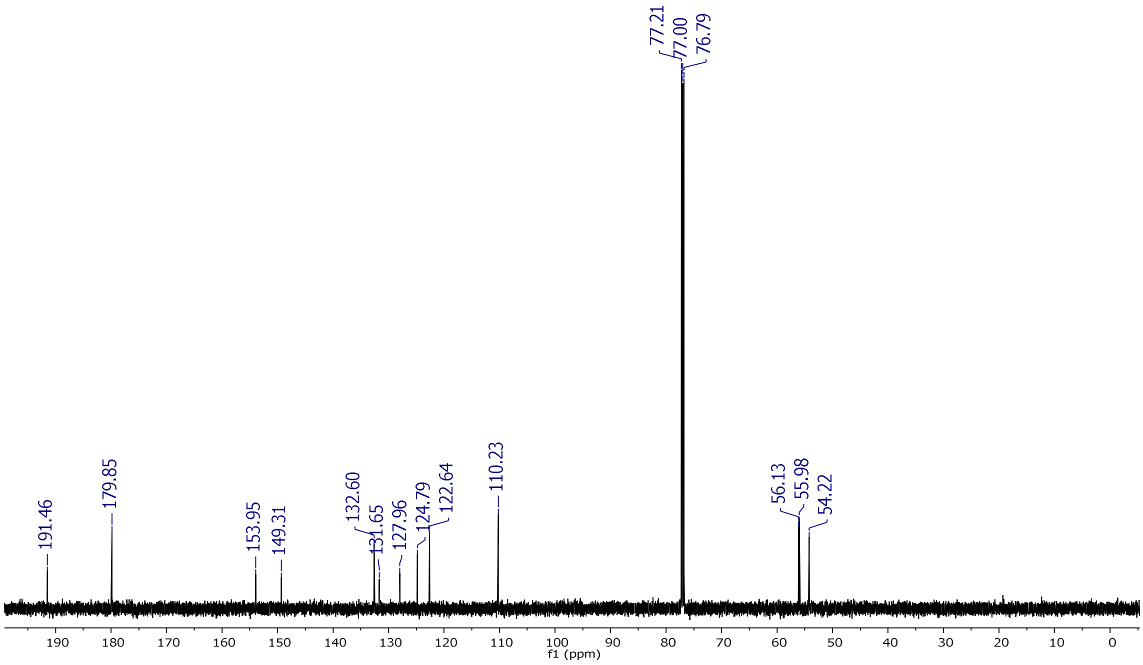

Figure S12. ^{13}C NMR (150 MHz, $CDCl_3$) spectrum of compound **8f**

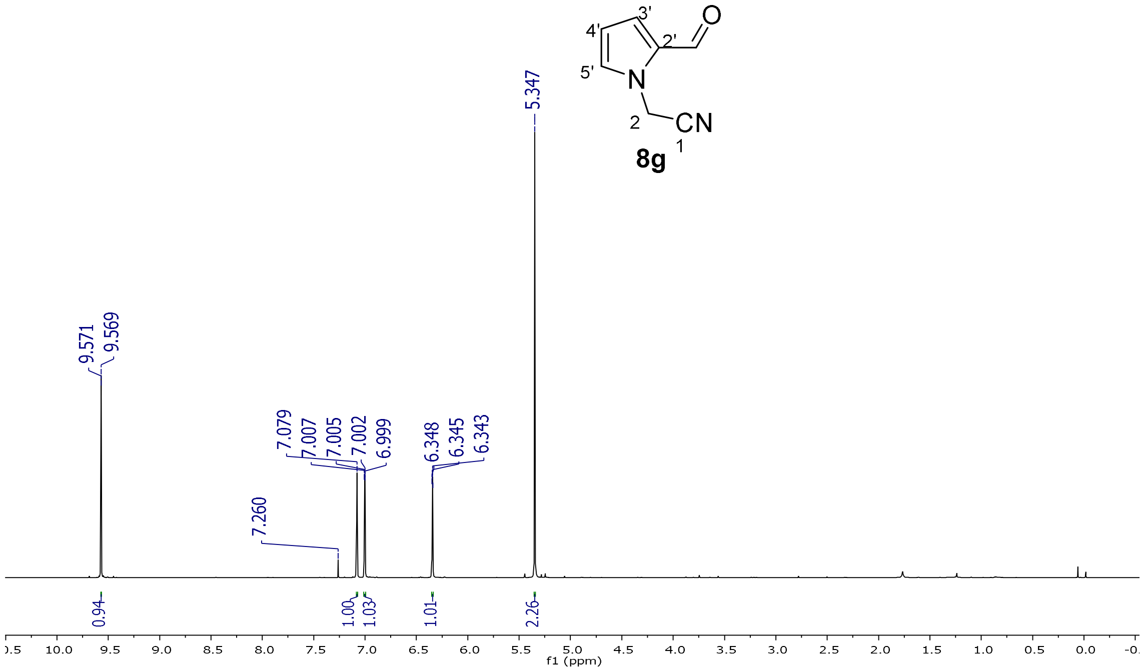

Figure S13. ^{1}H NMR (750 MHz, $CDCl_3$) spectrum of compound **8g**

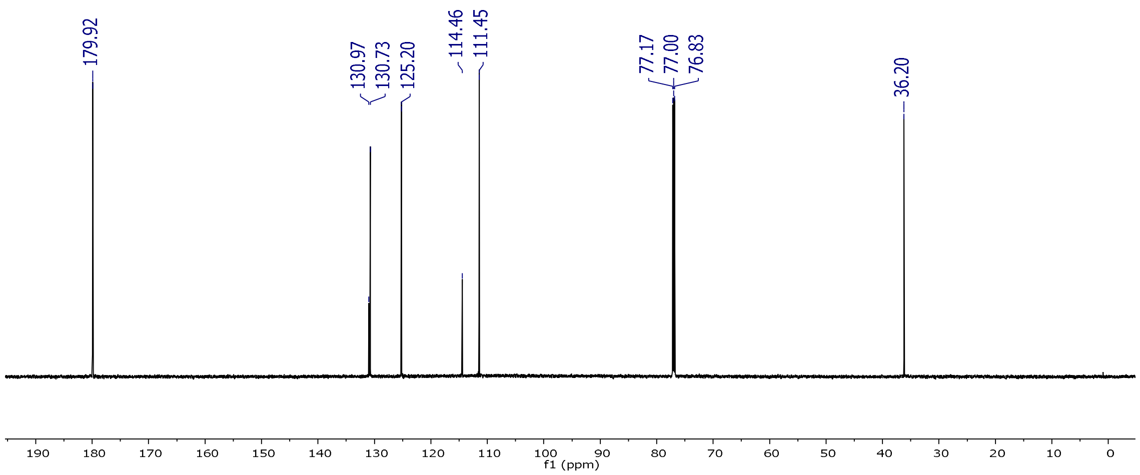

Figure S14. ^{13}C NMR (187.5 MHz, $CDCl_3$) spectrum of compound **8g**

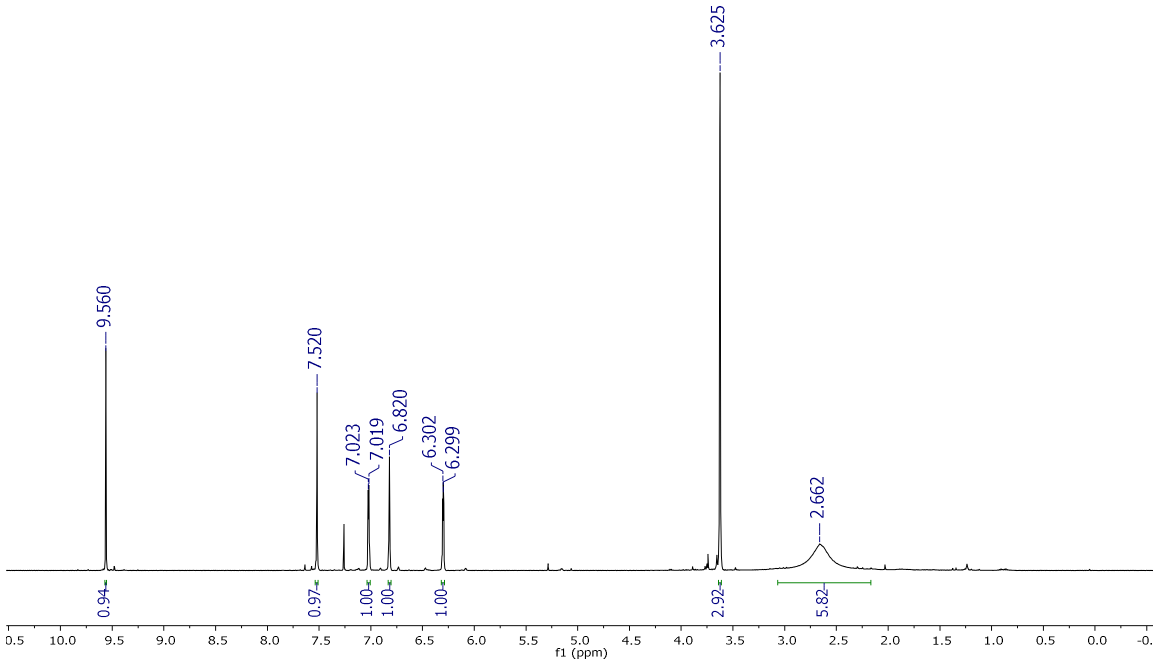

Figure S15. ^{1}H NMR (500 MHz, $CDCl_3$) spectrum of compound **1a**

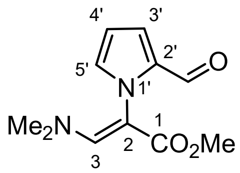

1a

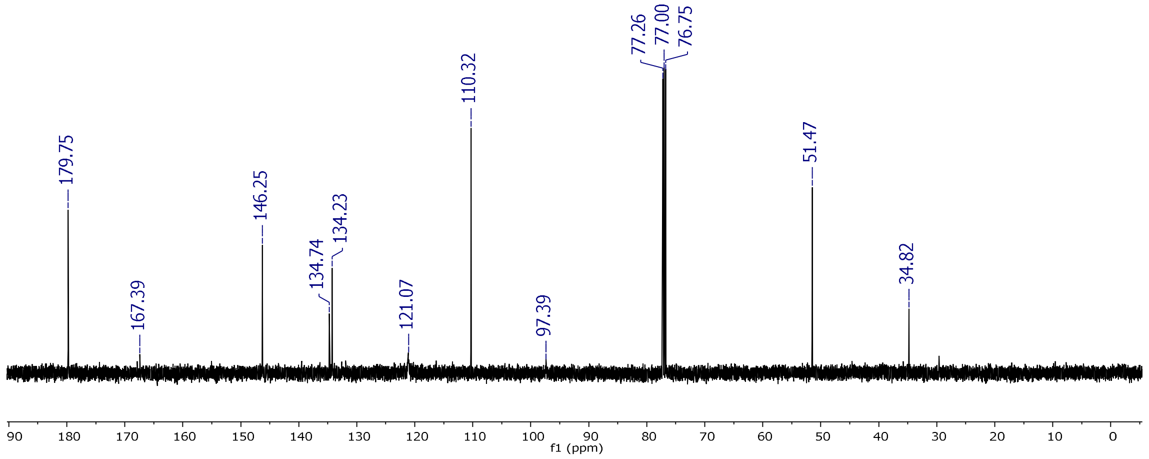

Figure S16. ^{13}C NMR (125 MHz, $CDCl_3$) spectrum of compound **1a**

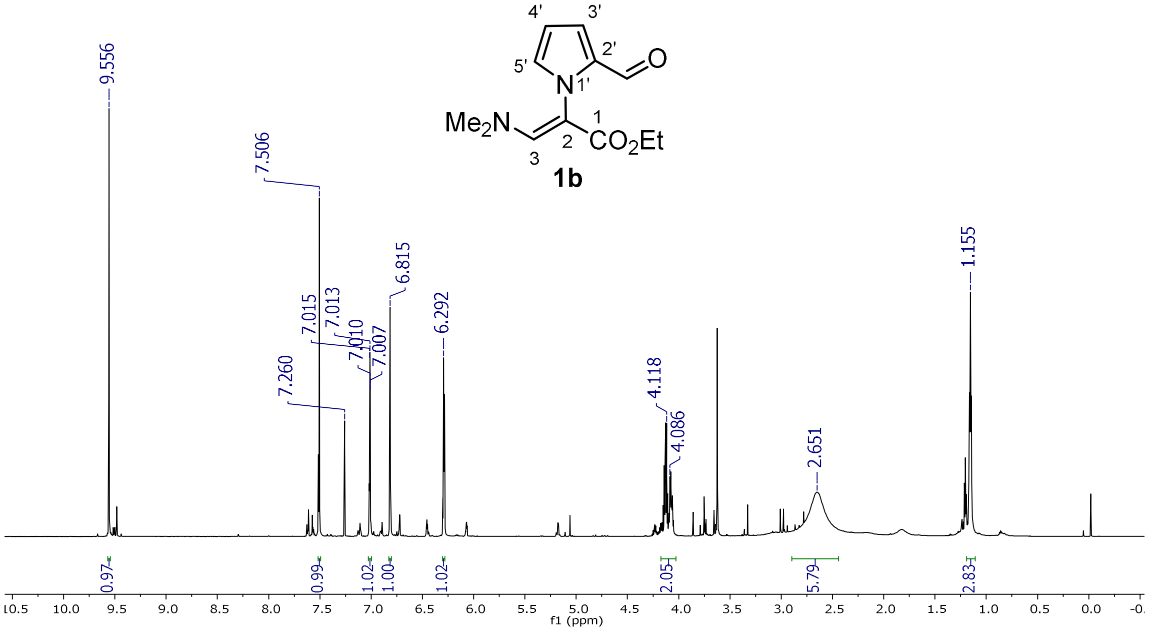

Figure S17. ^{1}H NMR (750 MHz, $CDCl_3$) spectrum of compound **1b**

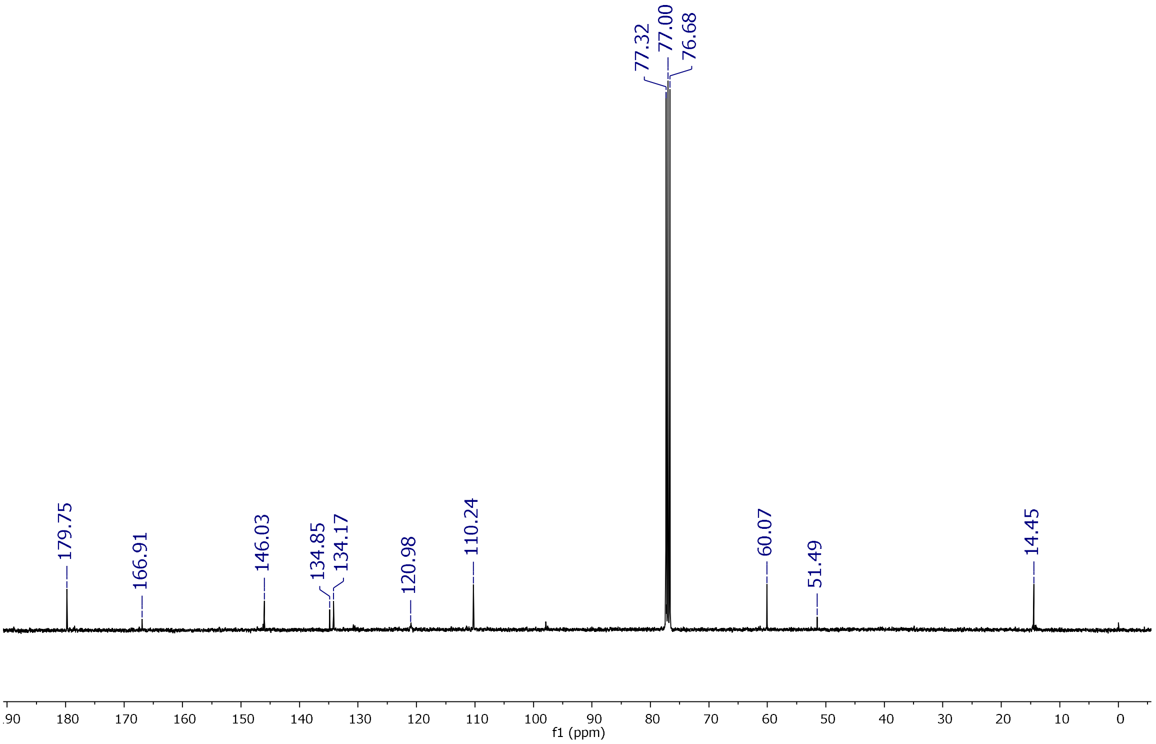

Figure S18. ^{13}C NMR (187.5 MHz, $CDCl_3$) spectrum of compound **1b**

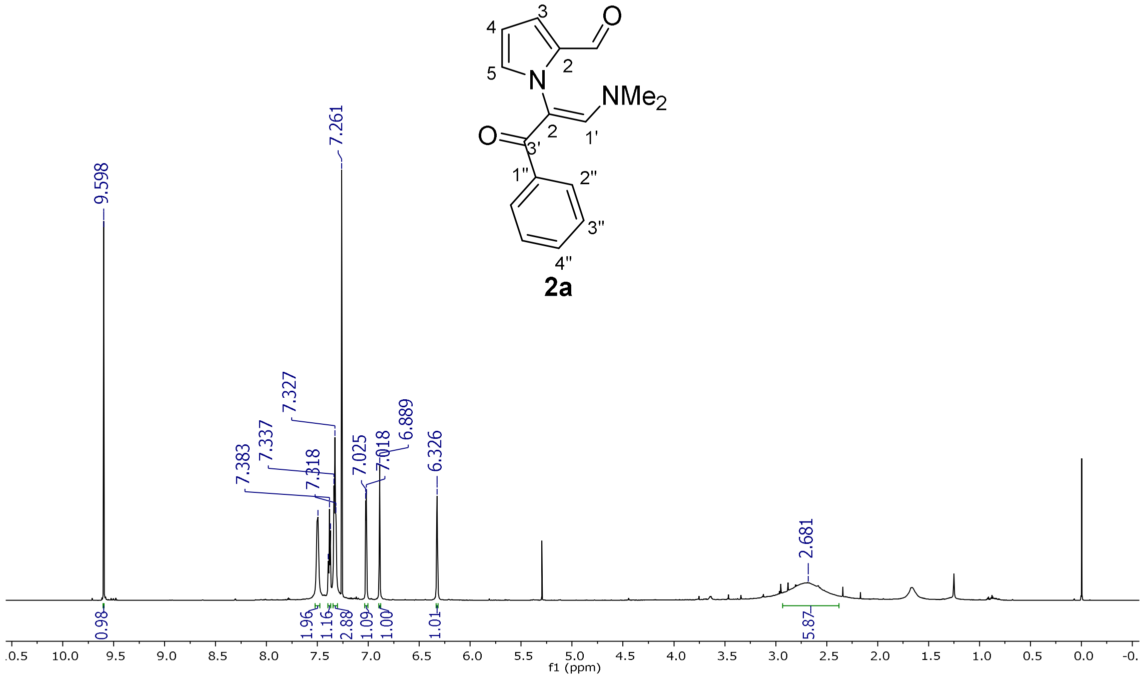

Figure S19. ^{1}H NMR (750 MHz, $CDCl_3$) spectrum of compound **2a**

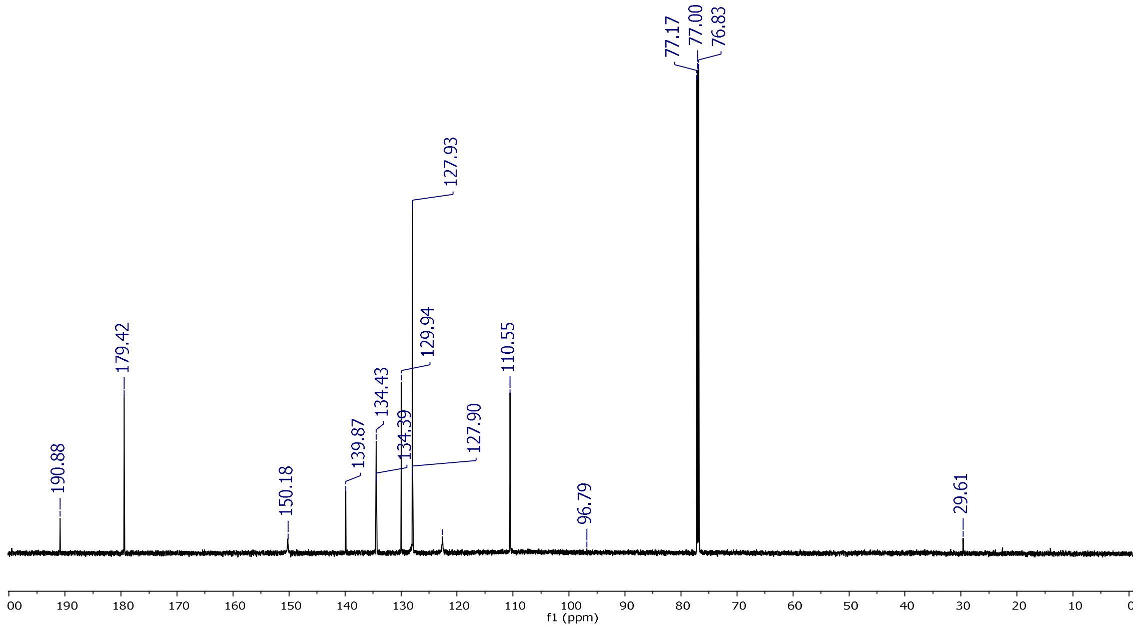

Figure S20. ^{13}C NMR (187.5 MHz, $CDCl_3$) spectrum of compound **2a**

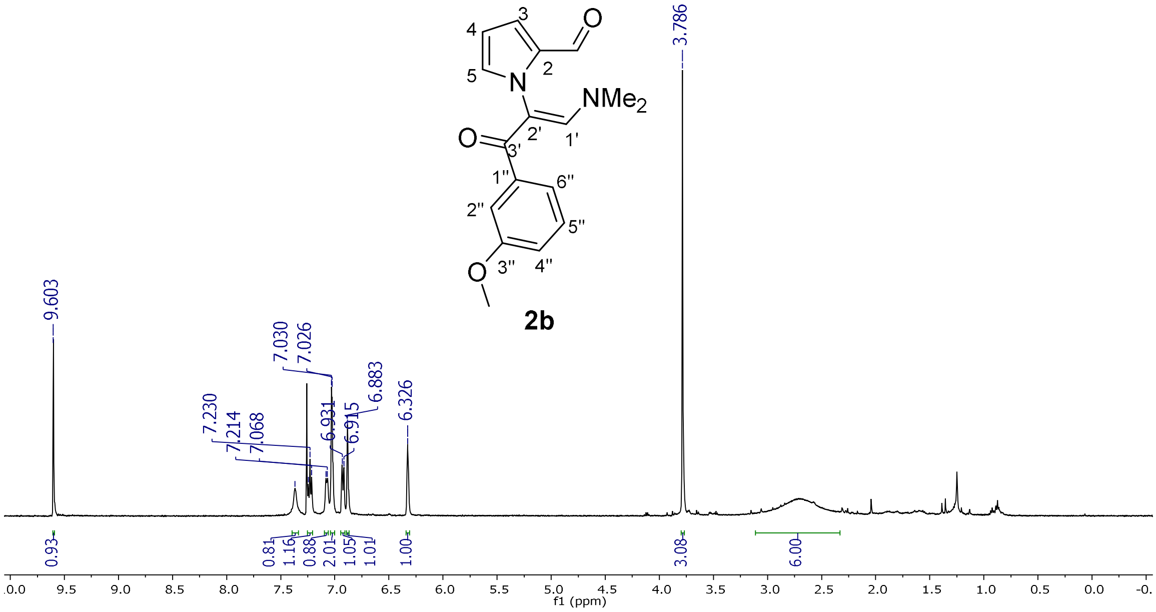

Figure S21. ^{1}H NMR (500 MHz, $CDCl_3$) spectrum of compound **2b**

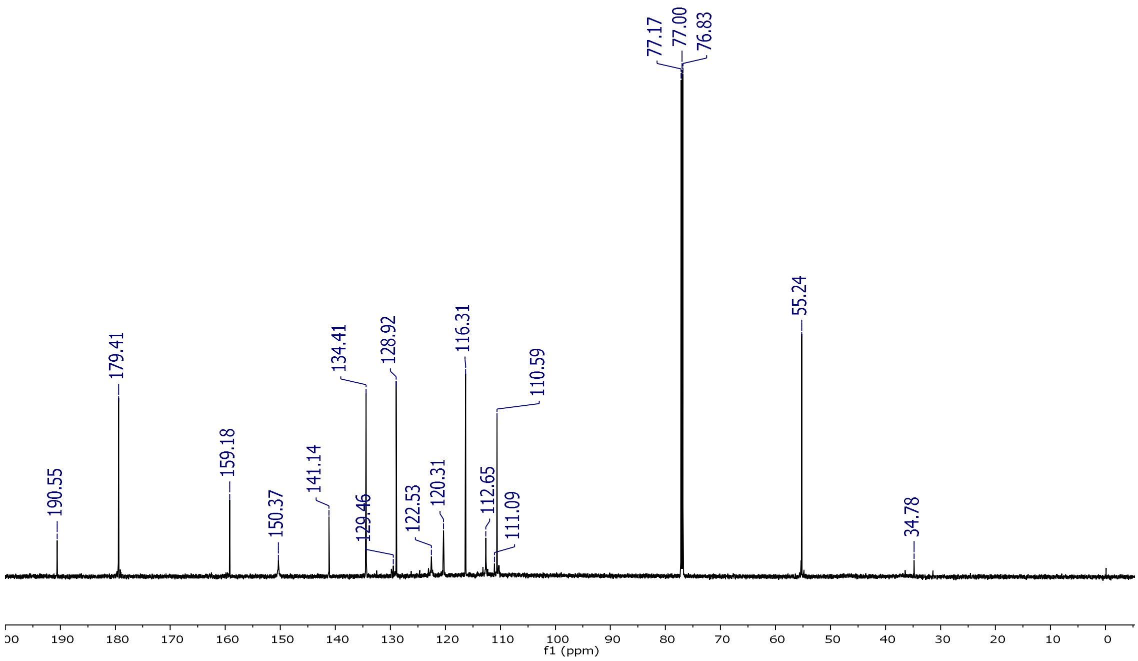

Figure S22. ^{13}C NMR (125 MHz, $CDCl_3$) spectrum of compound **2b**

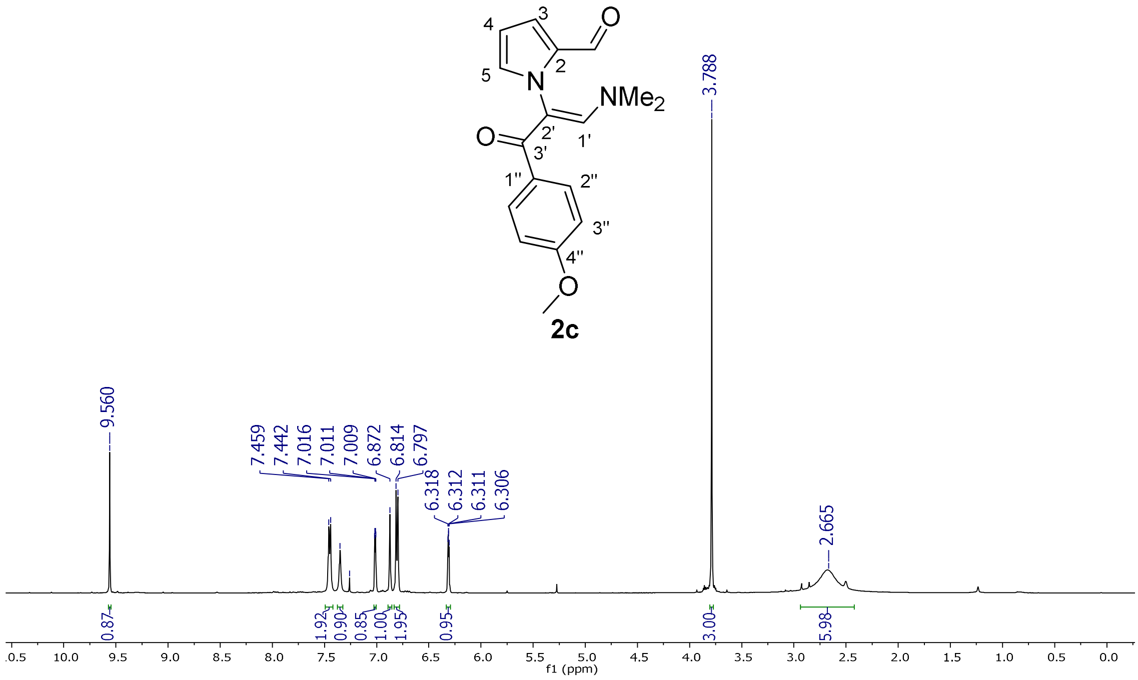

Figure S23. ^{1}H NMR (500 MHz, $CDCl_3$) spectrum of compound **2c**

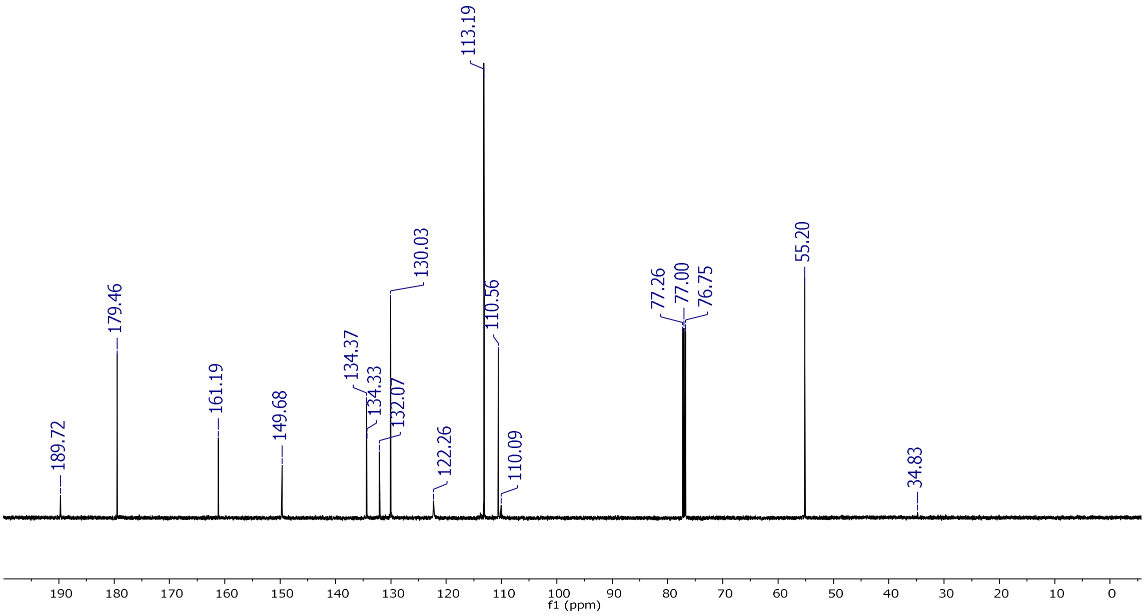

Figure S24. ^{13}C NMR (125 MHz, $CDCl_3$) spectrum of compound **2c**

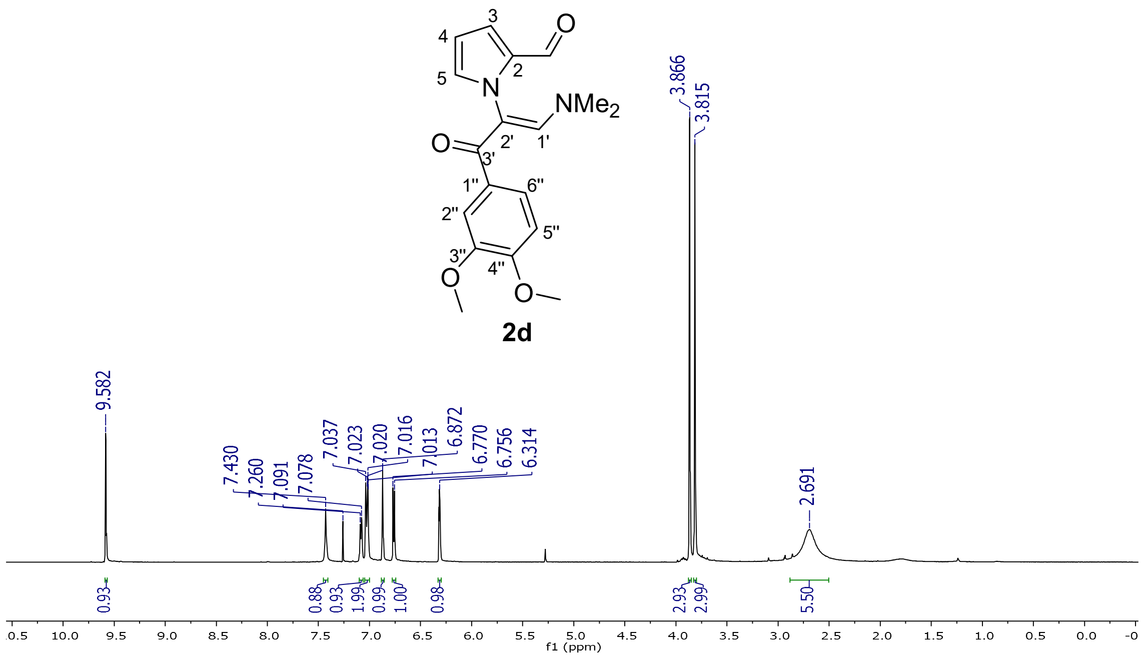

Figure S25. ^{1}H NMR (600 MHz, $CDCl_3$) spectrum of compound **2d**

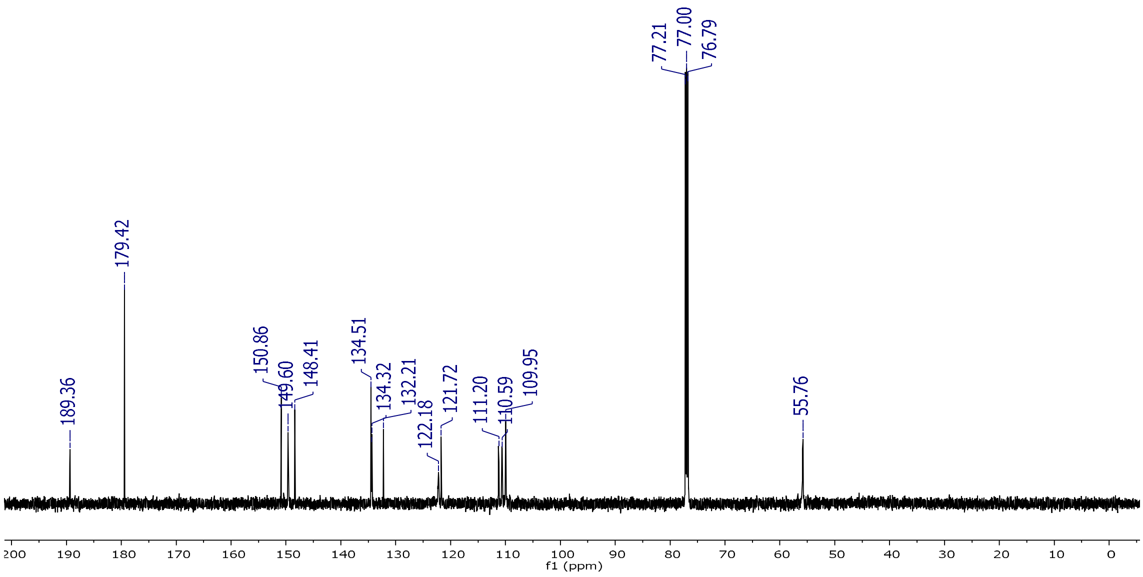

Figure S26. ^{13}C NMR (150 MHz, $CDCl_3$) spectrum of compound **2d**

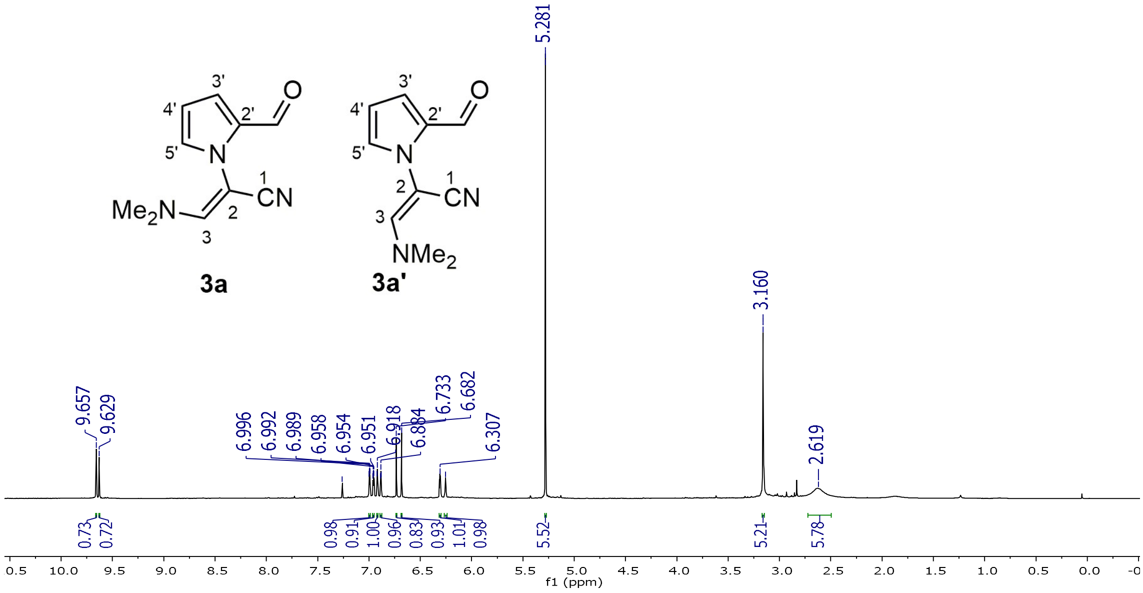

Figure S27. ^{1}H NMR (600 MHz, $CDCl_3$) spectrum of compounds **3a** and **3a′**

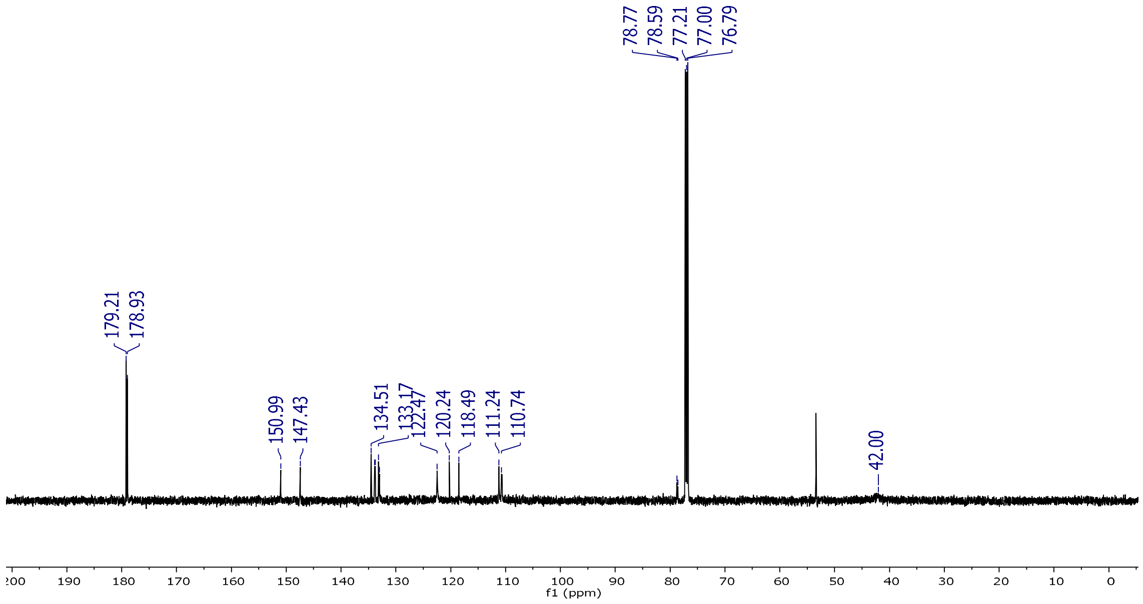

Figure S28. ^{13}C NMR (150 MHz, $CDCl_3$) spectrum of compounds **3a** and **3a'**

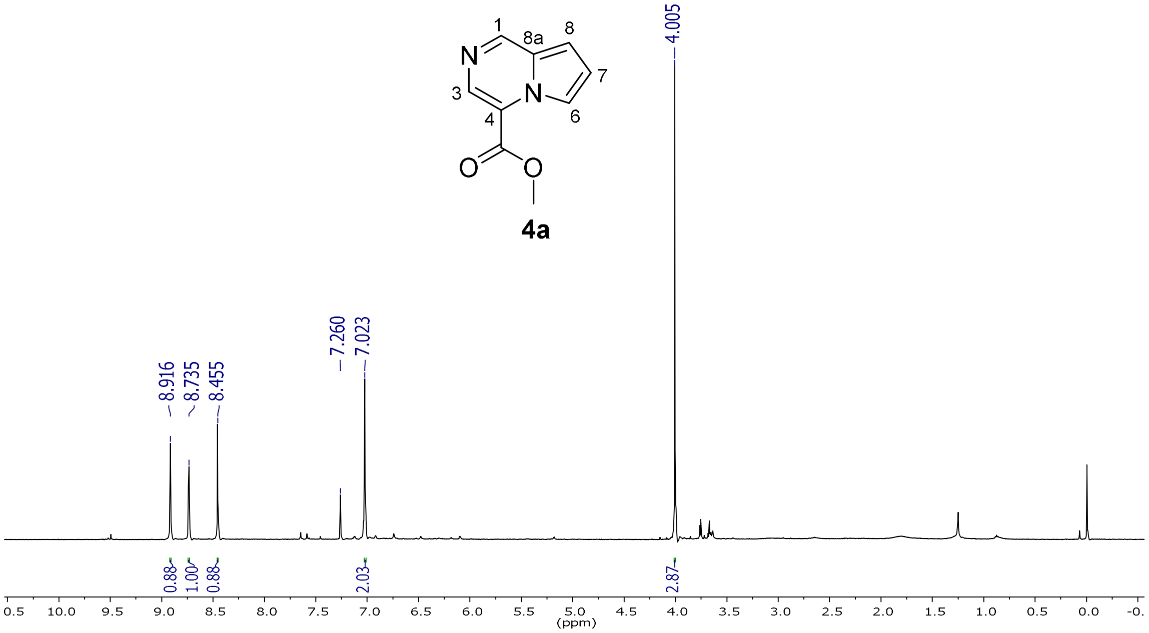

Figure S29. ^{1}H NMR (500 MHz, $CDCl_3$) spectrum of compound **4a**

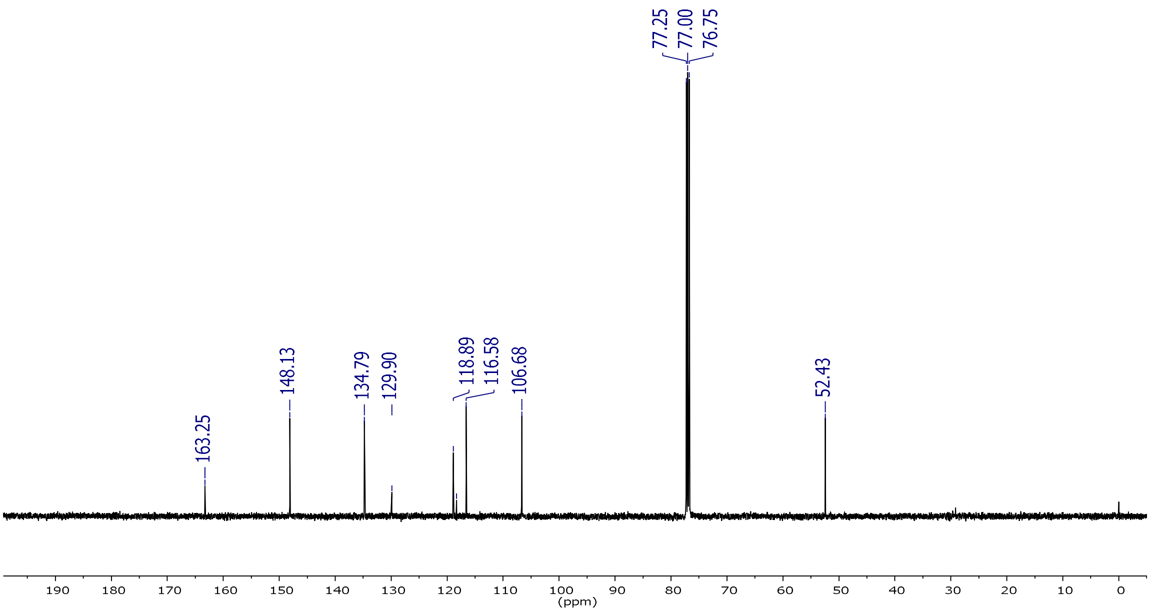

Figure S30. ^{13}C NMR (125 MHz, $CDCl_3$) spectrum of compound **4a**

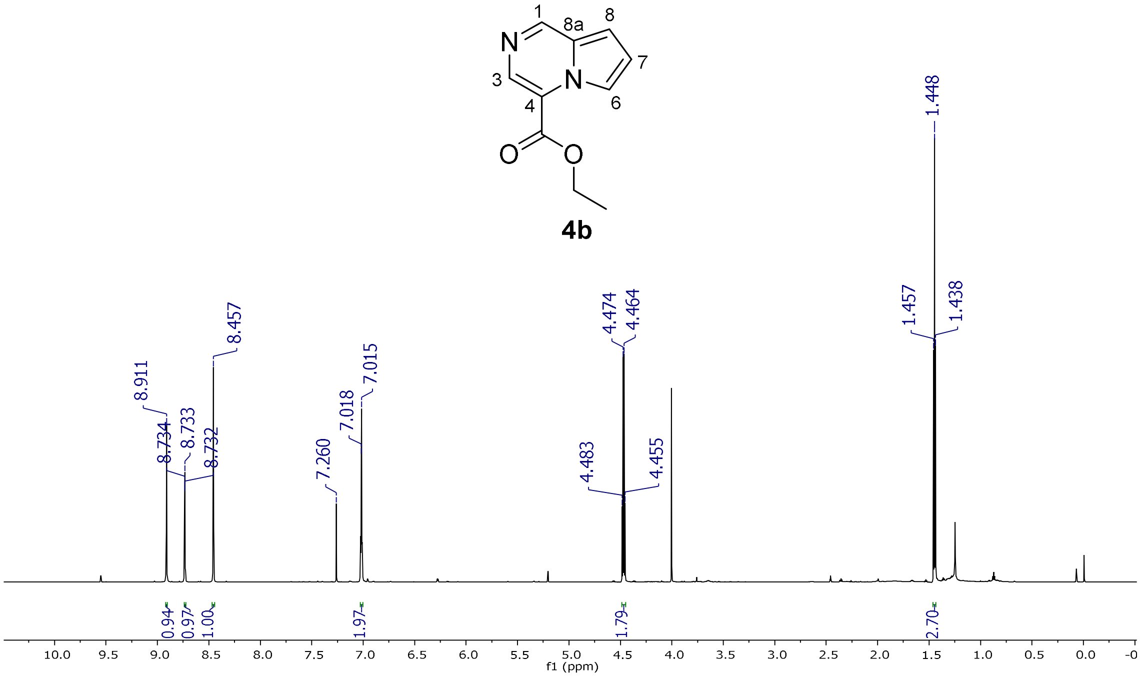

Figure S31. ^{1}H NMR (750 MHz, $CDCl_3$) spectrum of compound **4b**

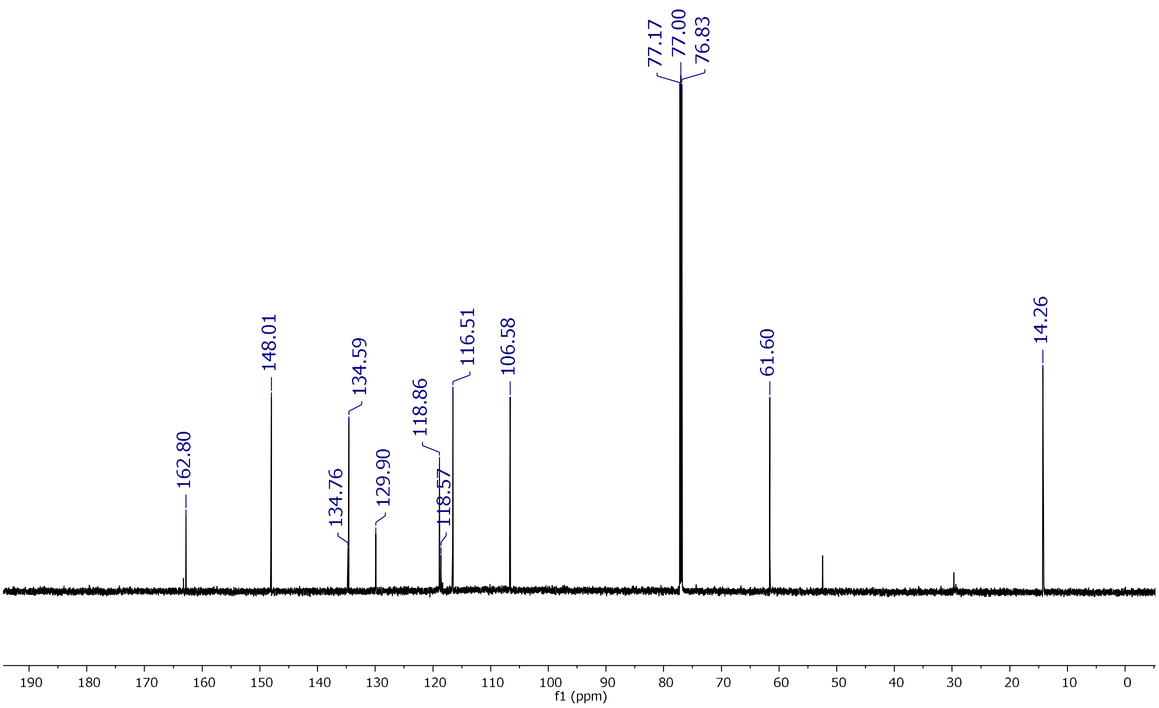

Figure S32. ^{13}C NMR (187.5 MHz, $CDCl_3$) spectrum of compound **4b**

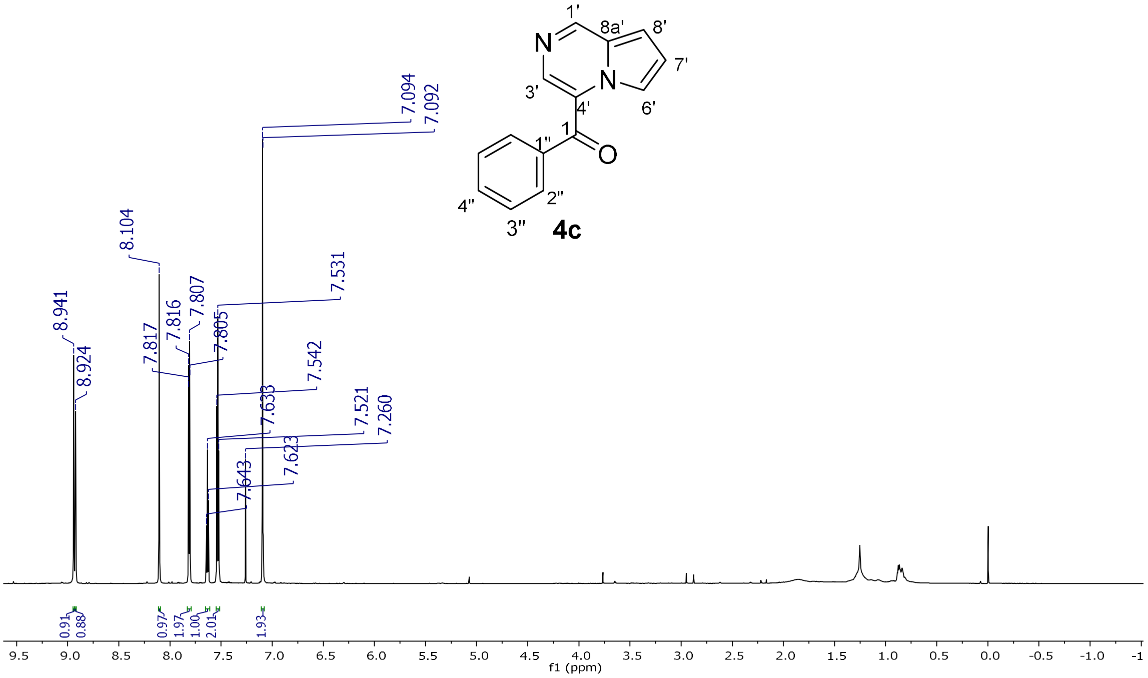

Figure S33. ^{1}H NMR (750 MHz, $CDCl_3$) spectrum of compound **4c**

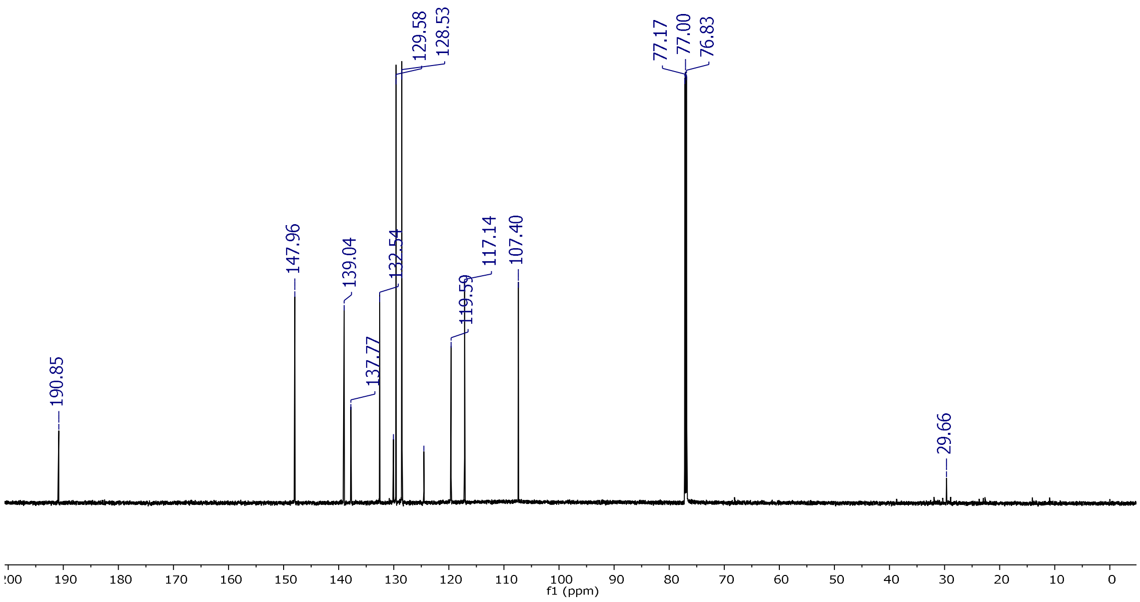

Figure S34. ^{13}C NMR (187.5 MHz, $CDCl_3$) spectrum of compound **4c**

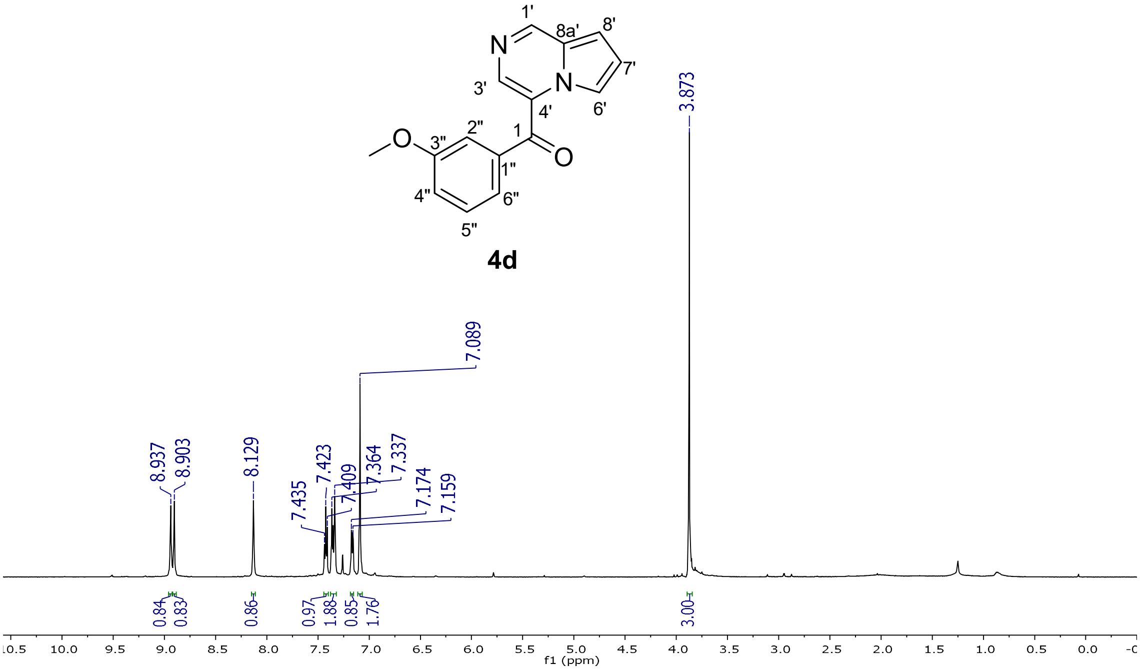

Figure S35. ^{1}H NMR (600 MHz, $CDCl_3$) spectrum of compound **4d**

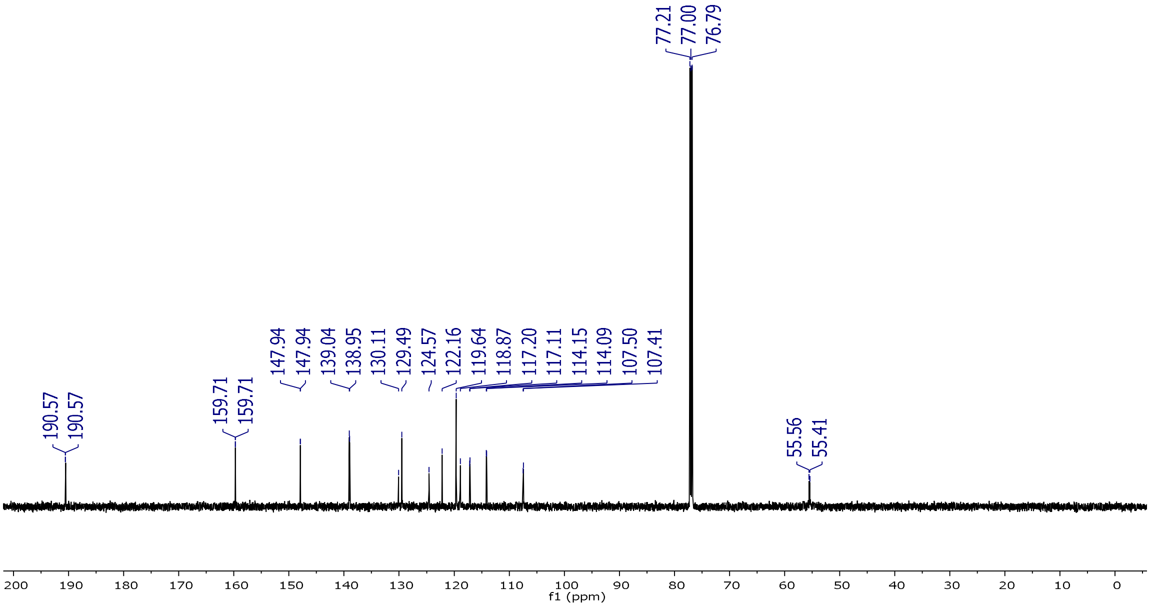

Figure S36. ^{13}C NMR (150 MHz, $CDCl_3$) spectrum of compound **4d**

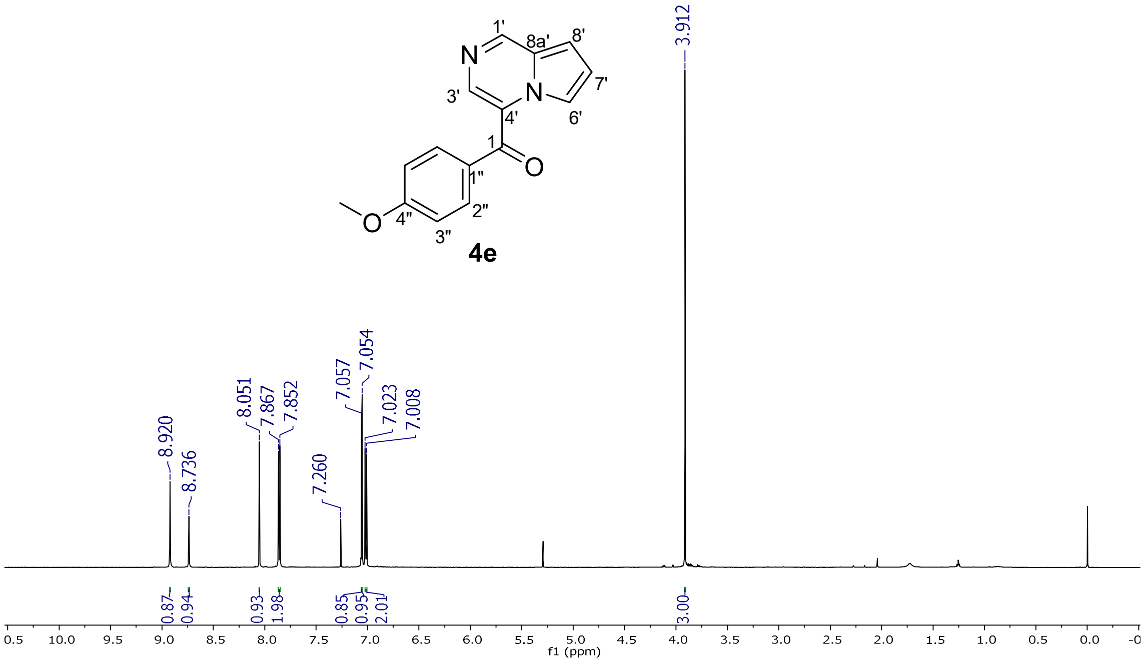

Figure S37. ^{1}H NMR (600 MHz, $CDCl_3$) spectrum of compound **4e**

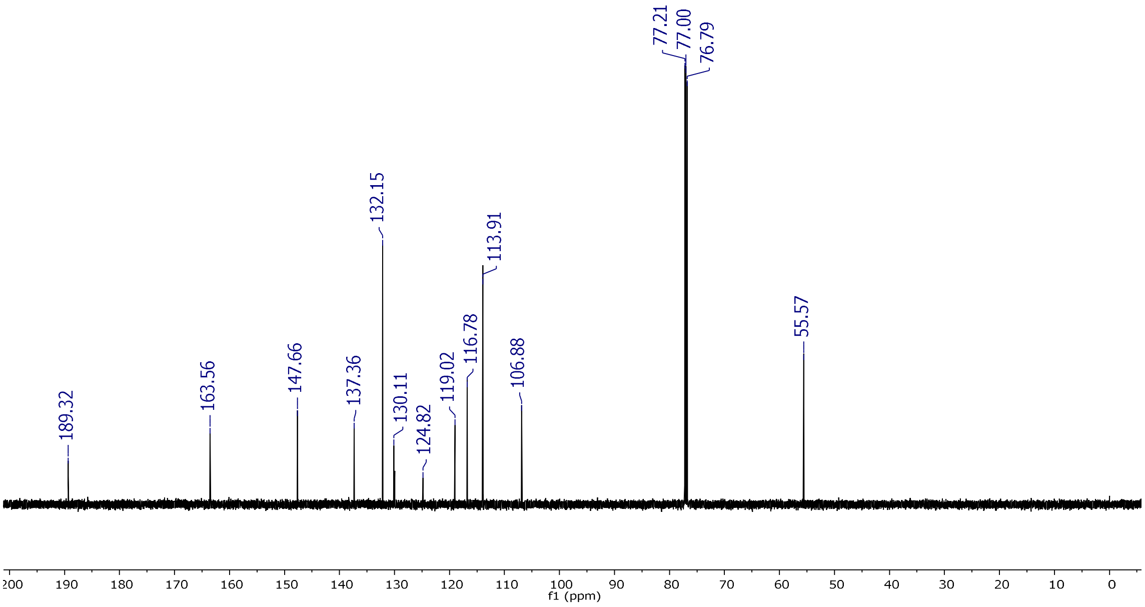

Figure S38. ^{13}C NMR (150 MHz, $CDCl_3$) spectrum of compound **4e**

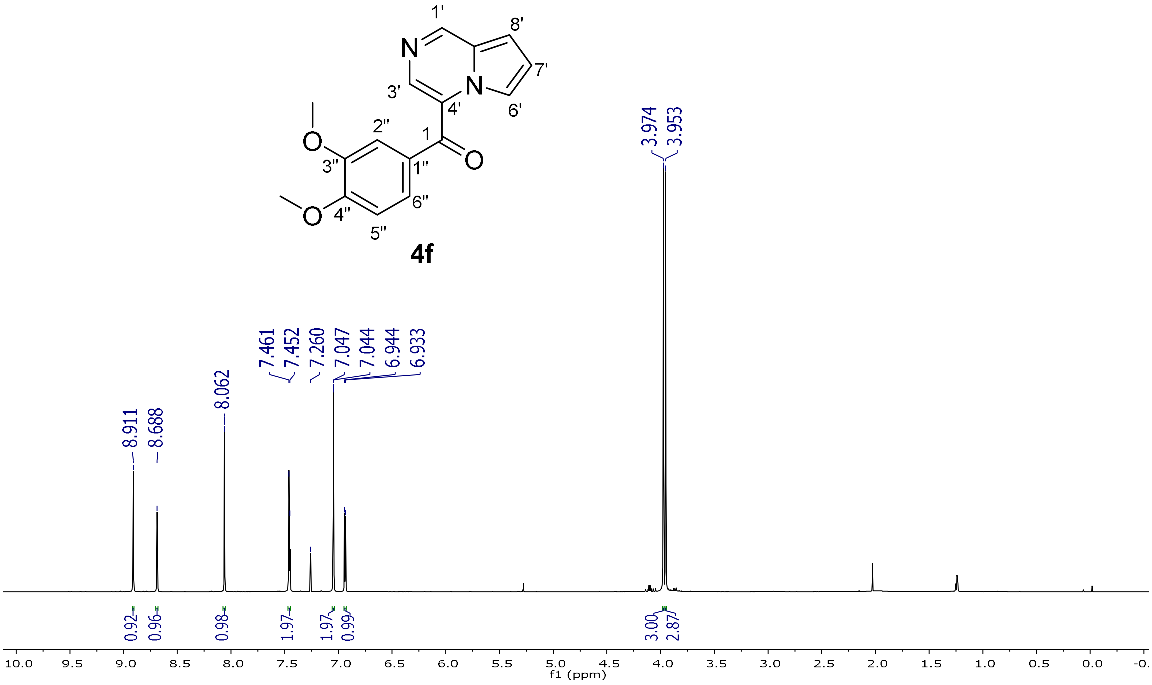

Figure S39. ^{1}H NMR (750 MHz, $CDCl_3$) spectrum of compound **4f**

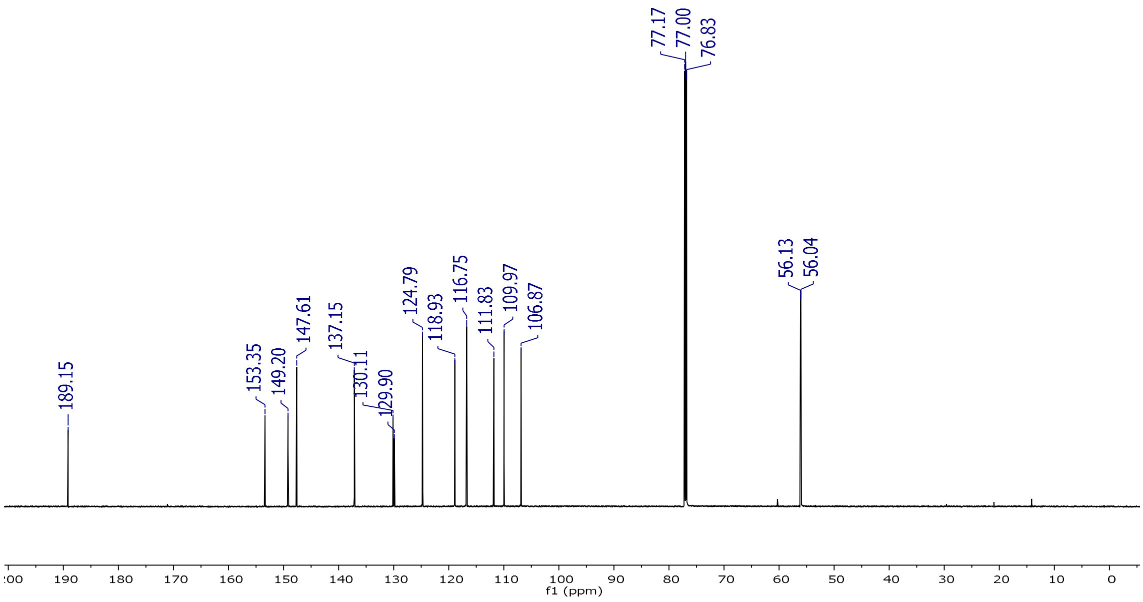

Figure S40. ^{13}C NMR (187.5 MHz, $CDCl_3$) spectrum of compound **4f**

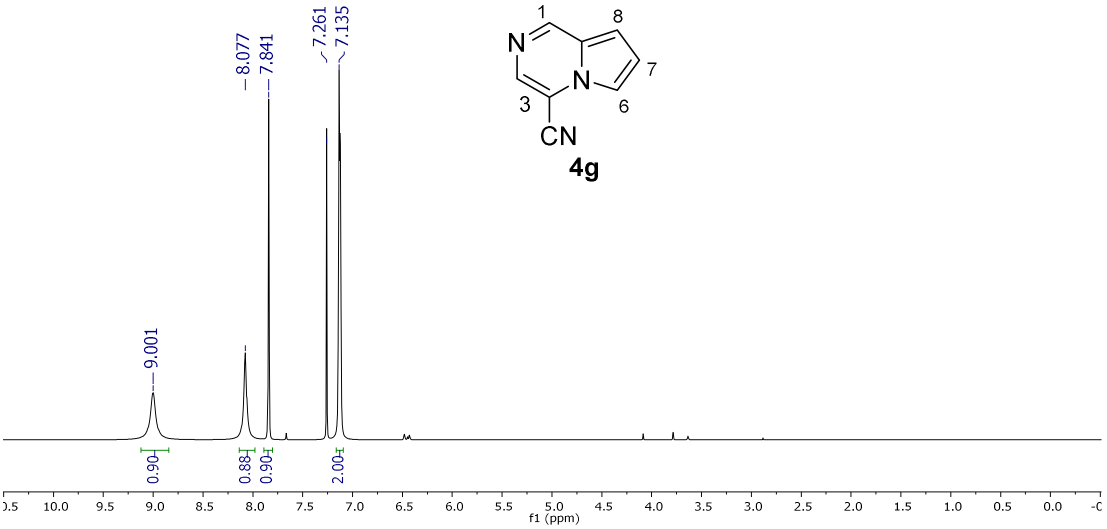

Figure S41. ^{1}H NMR (600 MHz, $CDCl_3$) spectrum of compound **4g**

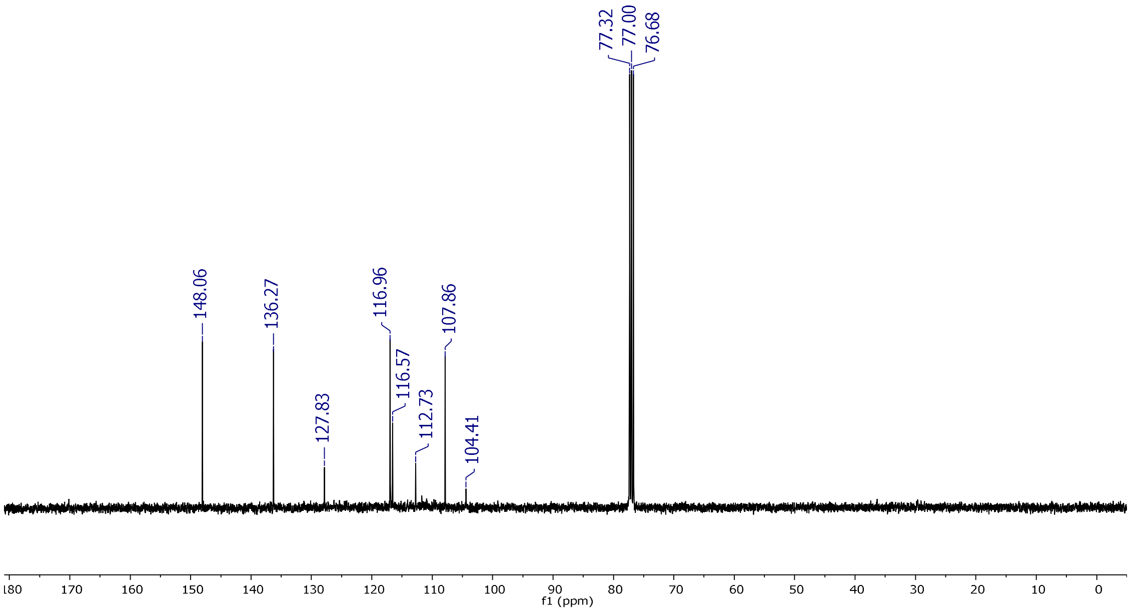

Figure S42. ^{13}C NMR (100 MHz, $CDCl_3$) spectrum of compound **4g**

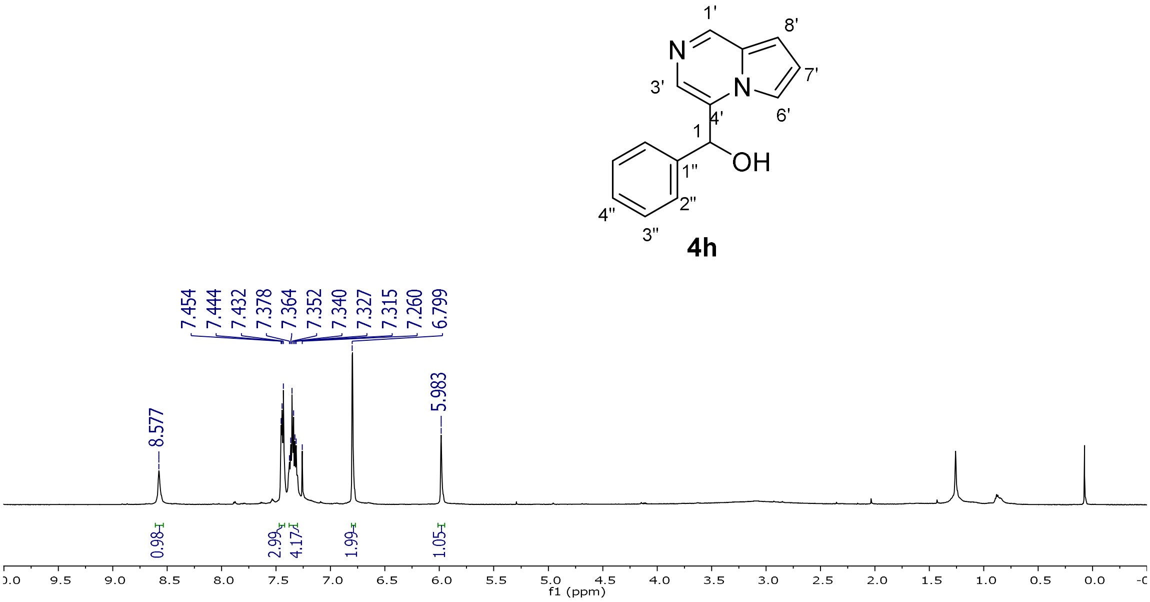

Figure S43. ^{1}H NMR (600 MHz, $CDCl_3$) spectrum of compound **4h**

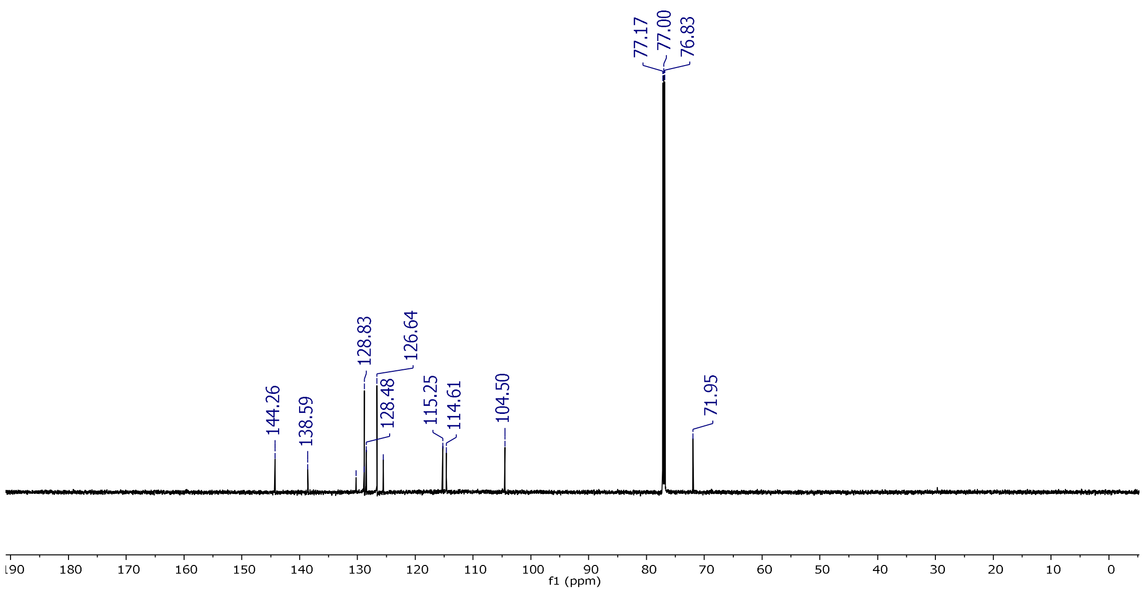

Figure S44. ^{13}C NMR (187.5 MHz, $CDCl_3$) spectrum of compound **4h**

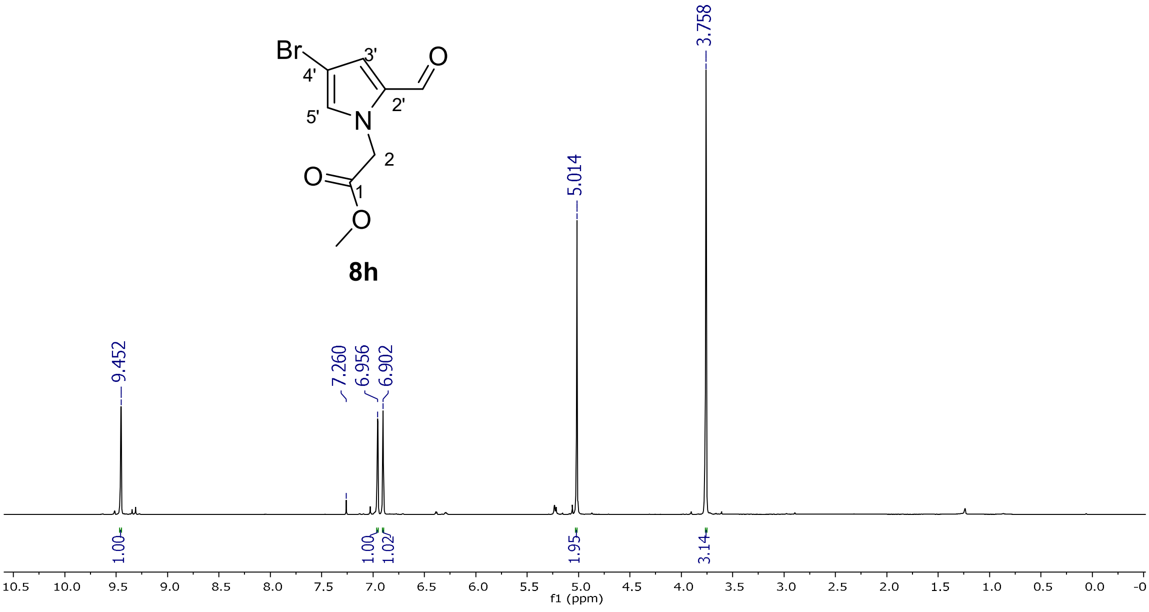

Figure S45. ^{1}H NMR (500 MHz, $CDCl_3$) spectrum of compound **8h**

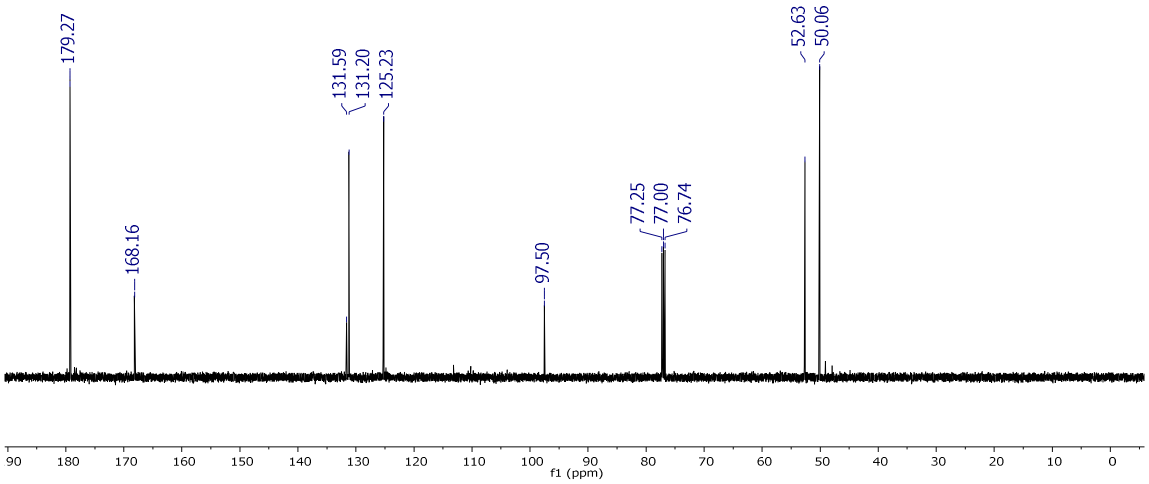

Figure S46. ^{13}C NMR (125 MHz, $CDCl_3$) spectrum of compound **8h**

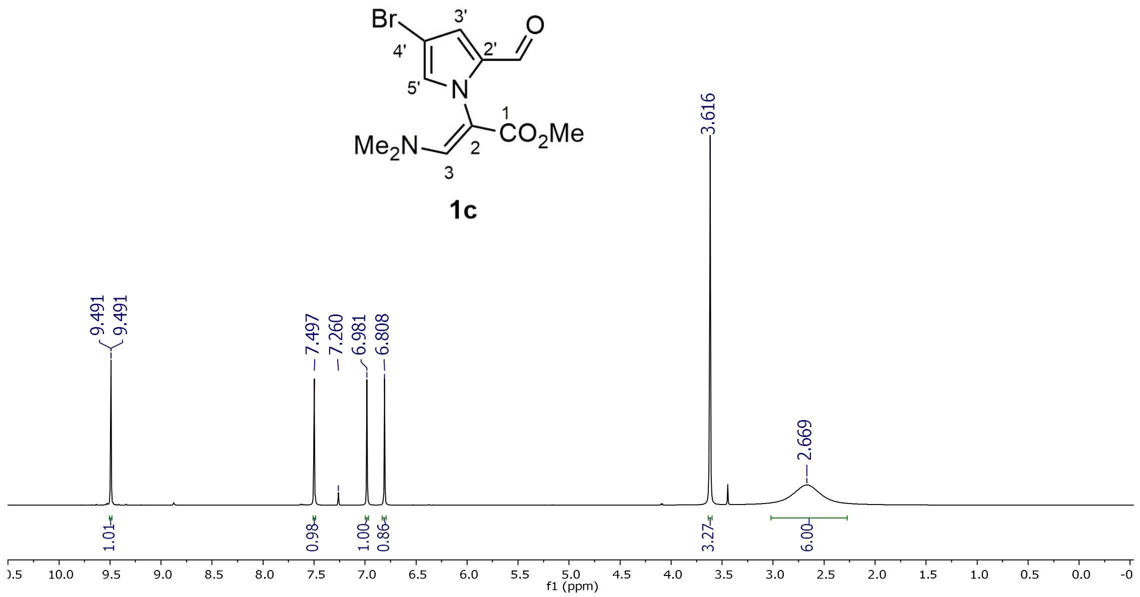

Figure S47. ^{1}H NMR (600 MHz, $CDCl_3$) spectrum of compound **1c**

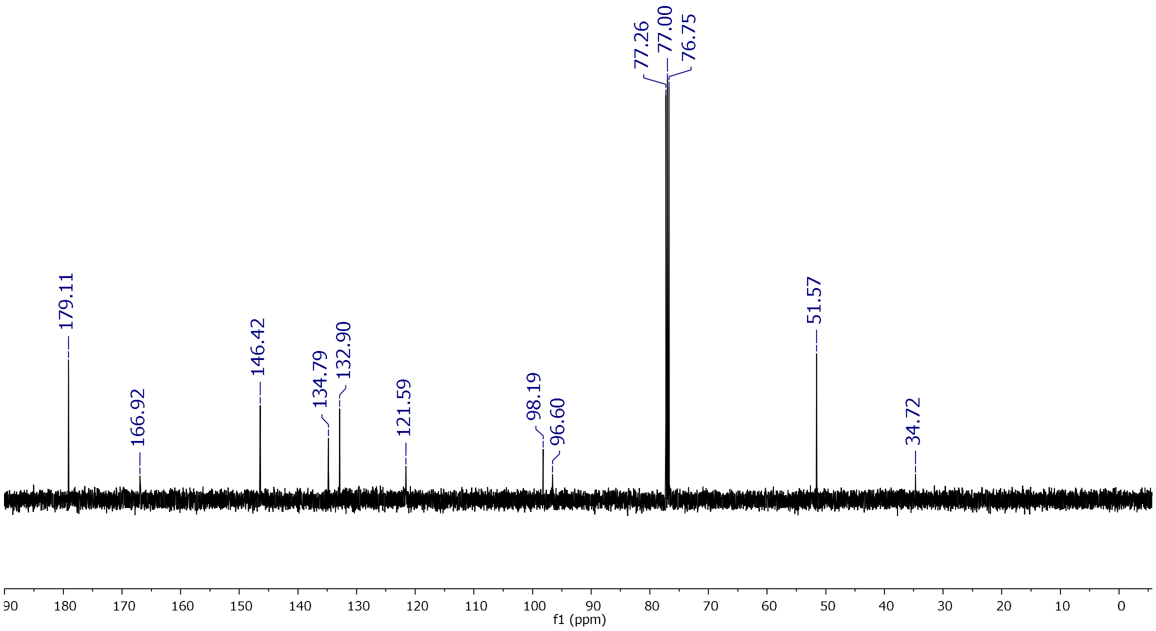

Figure S48. ^{13}C NMR (125 MHz, $CDCl_3$) spectrum of compound **1c**

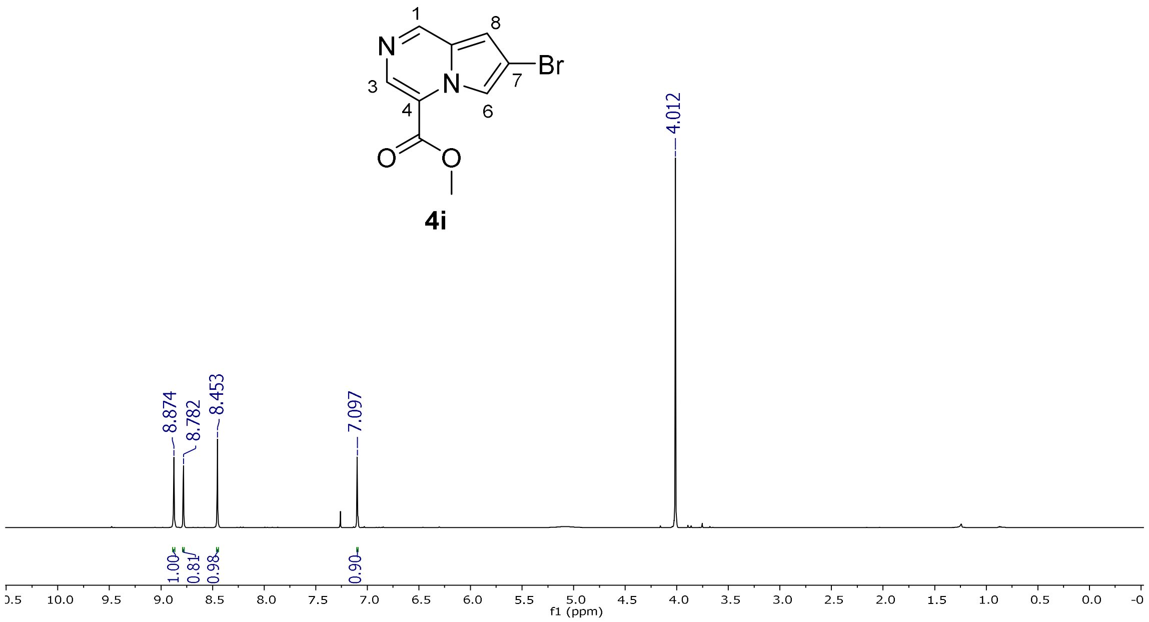

Figure S49. ^{1}H NMR (500 MHz, $CDCl_3$) spectrum of compound **4i**

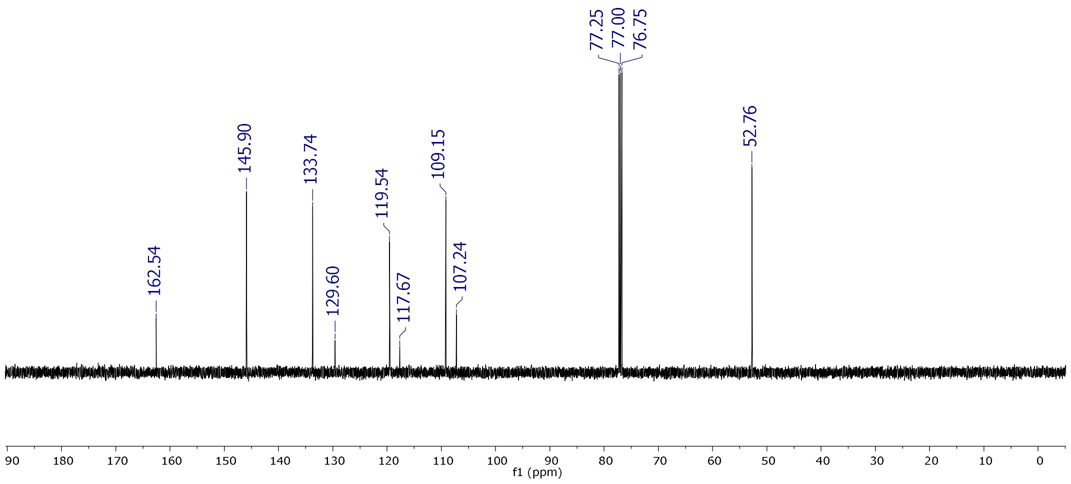

Figure S50. ^{13}C NMR (150 MHz, $CDCl_3$) spectrum of compound **4i**

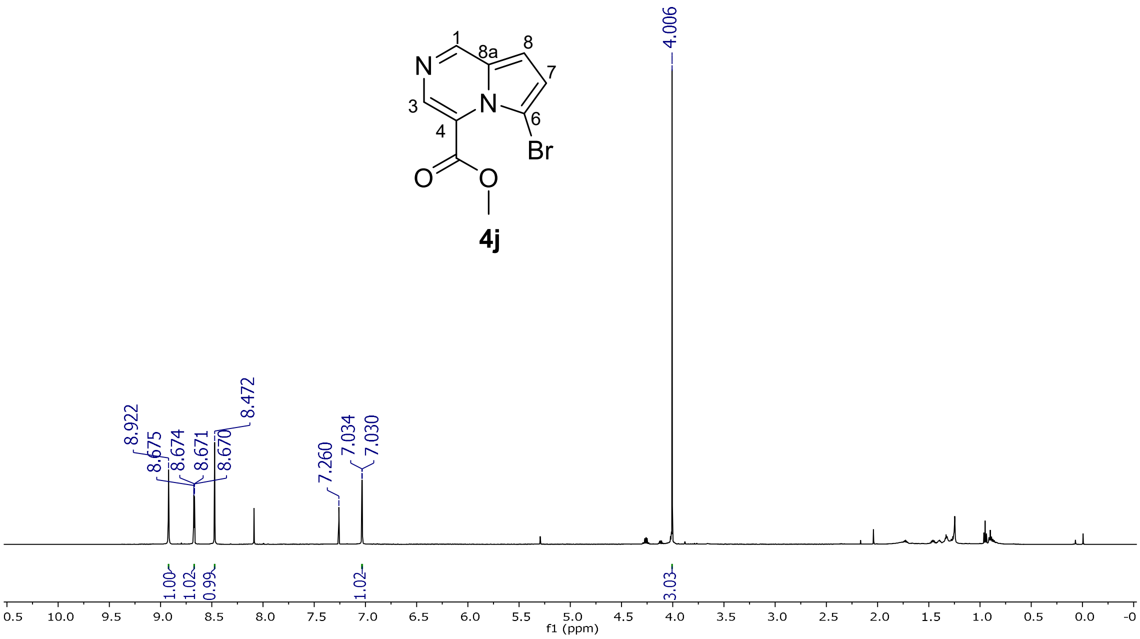

Figure S51. ^{1}H NMR (600 MHz, $CDCl_3$) spectrum of compound **4j**

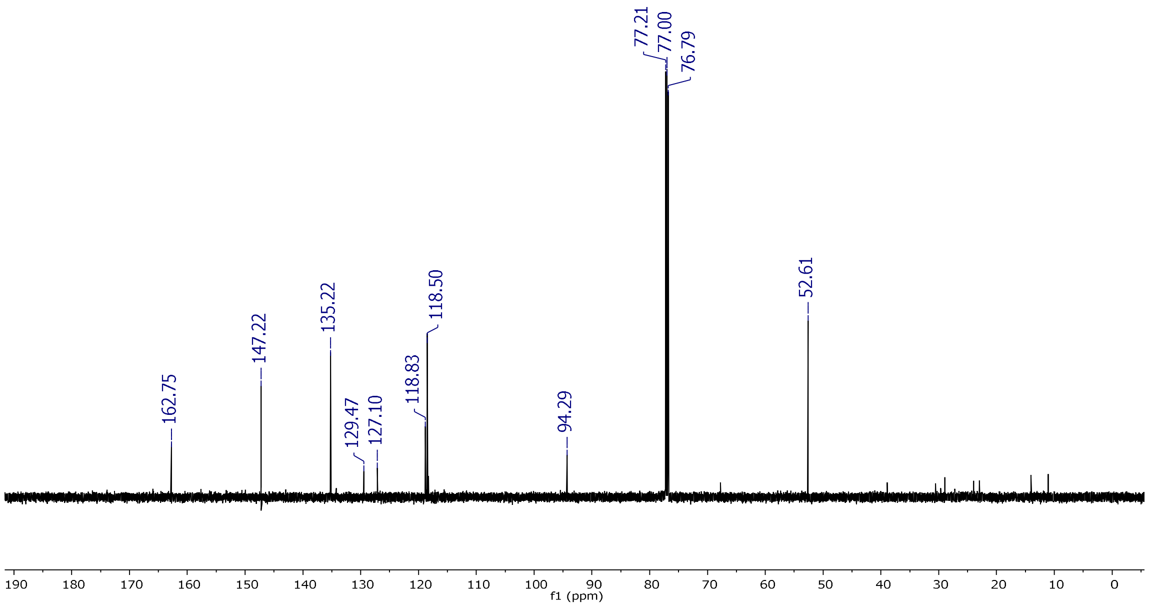

Figure S52. ^{13}C NMR (150 MHz, $CDCl_3$) spectrum of compound **4j**

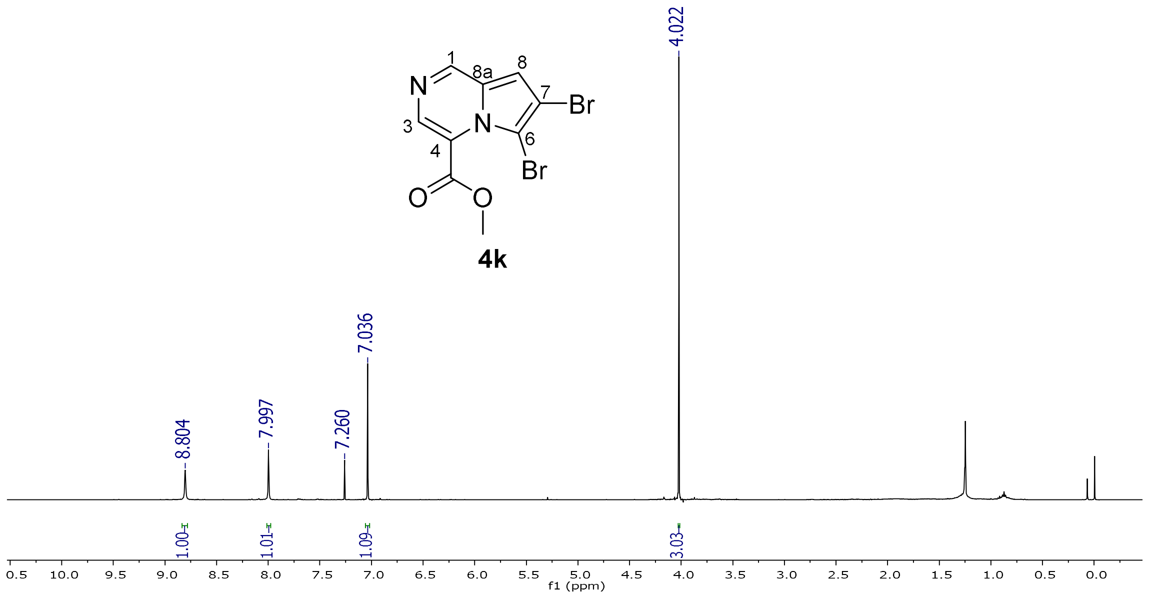

Figure S53. ^{1}H NMR (500 MHz, $CDCl_3$) spectrum of compound **4k**

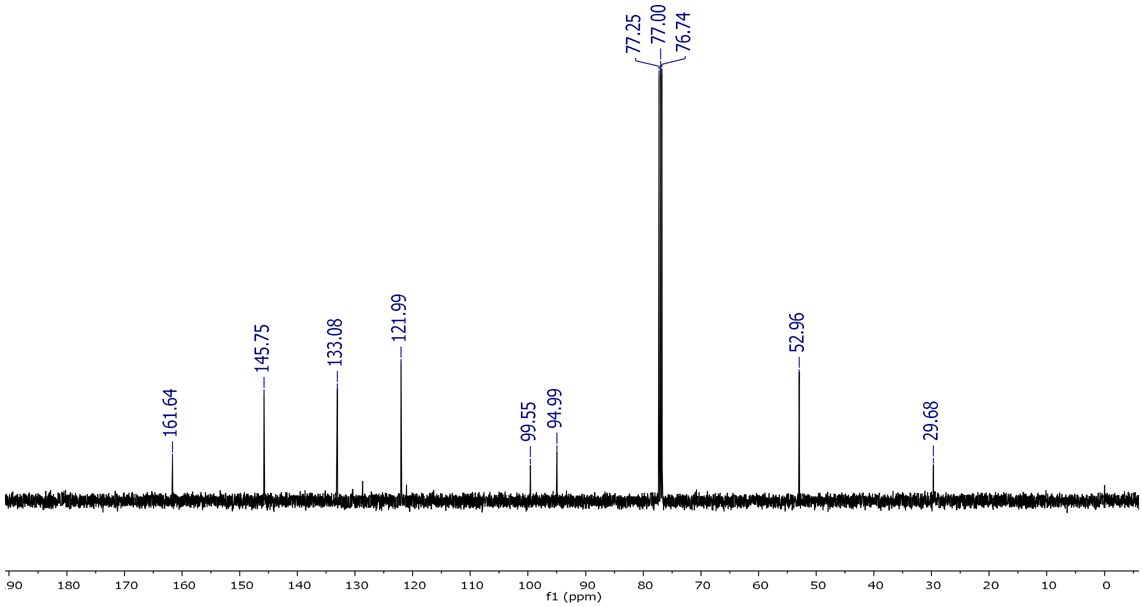

Figure S54. ^{13}C NMR (125 MHz, $CDCl_3$) spectrum of compound **4k**

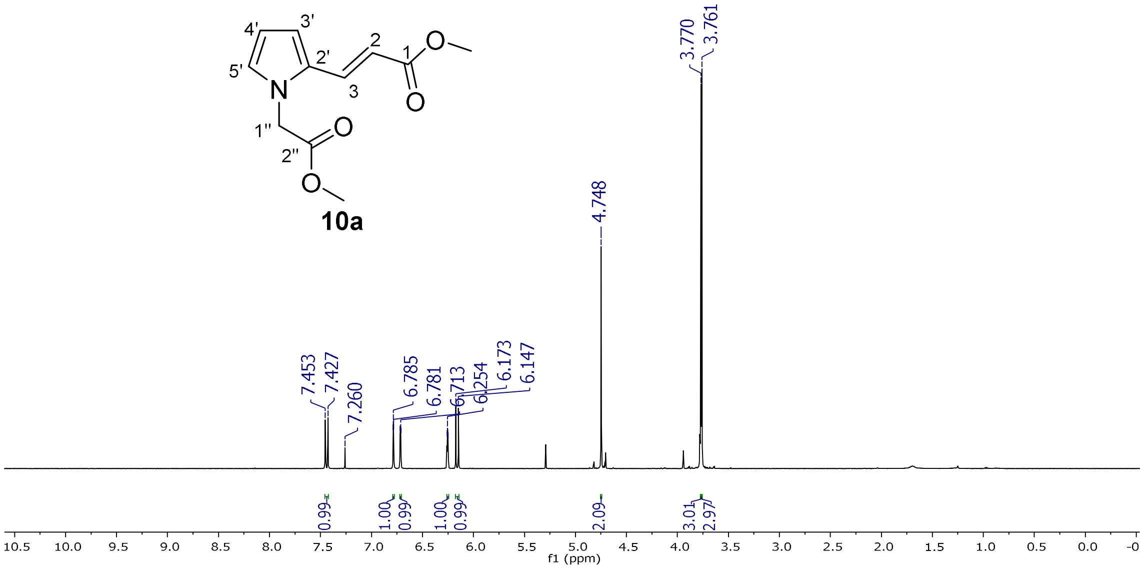

Figure S55. ^{1}H NMR (600 MHz, $CDCl_3$) spectrum of compound **10a**

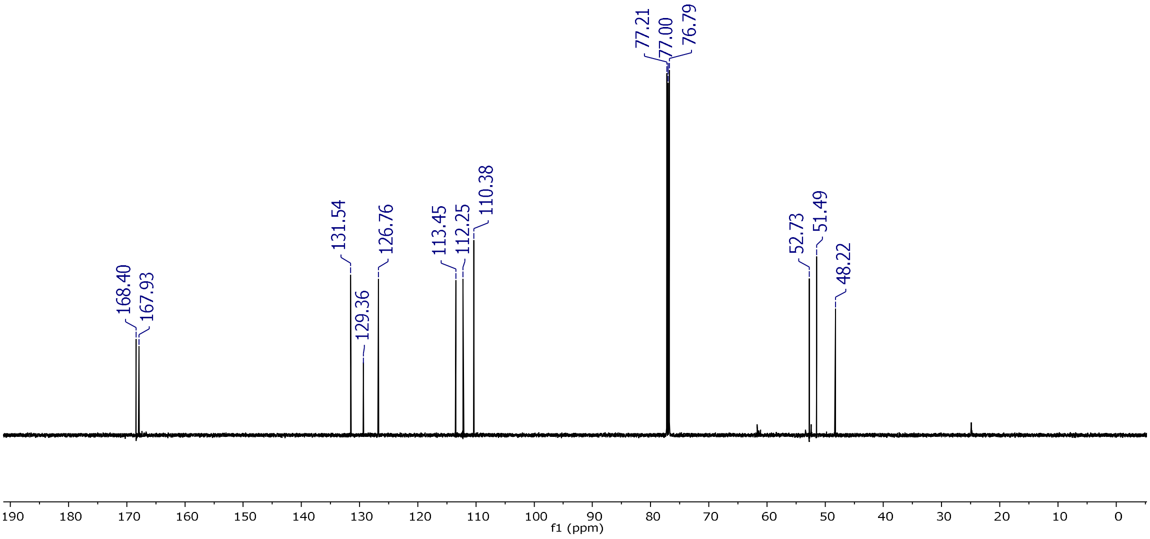

Figure S56. ¹³C NMR (150 MHz, $CDCl_3$) spectrum of compound **10a**

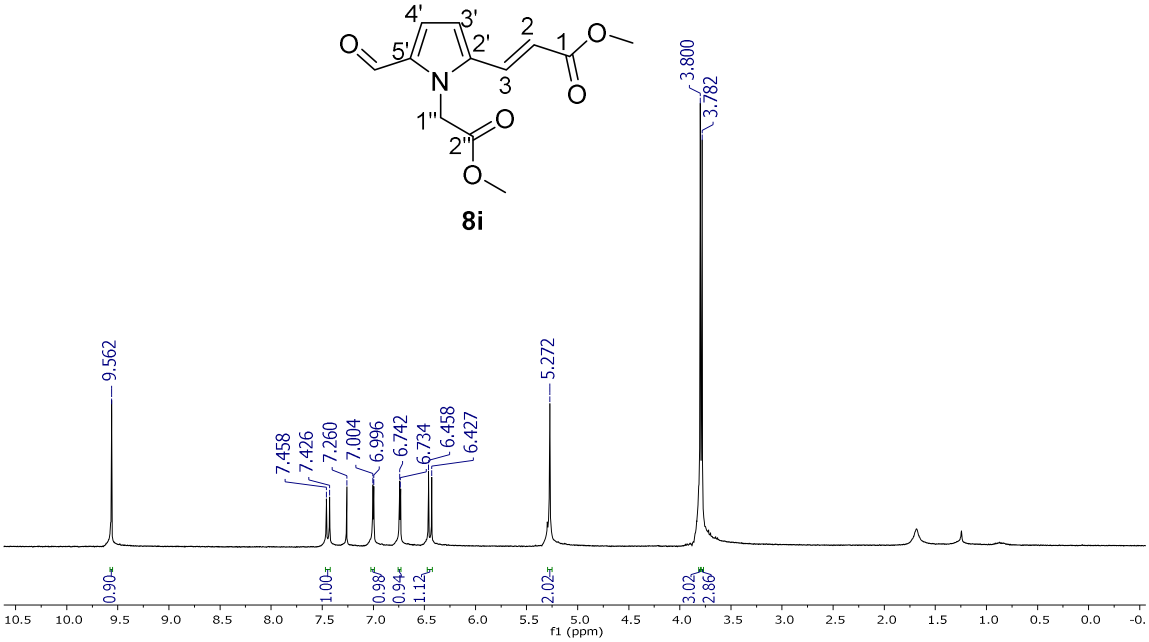

Figure S57. ¹H NMR (500 MHz, $CDCl_3$) spectrum of compound **8i**

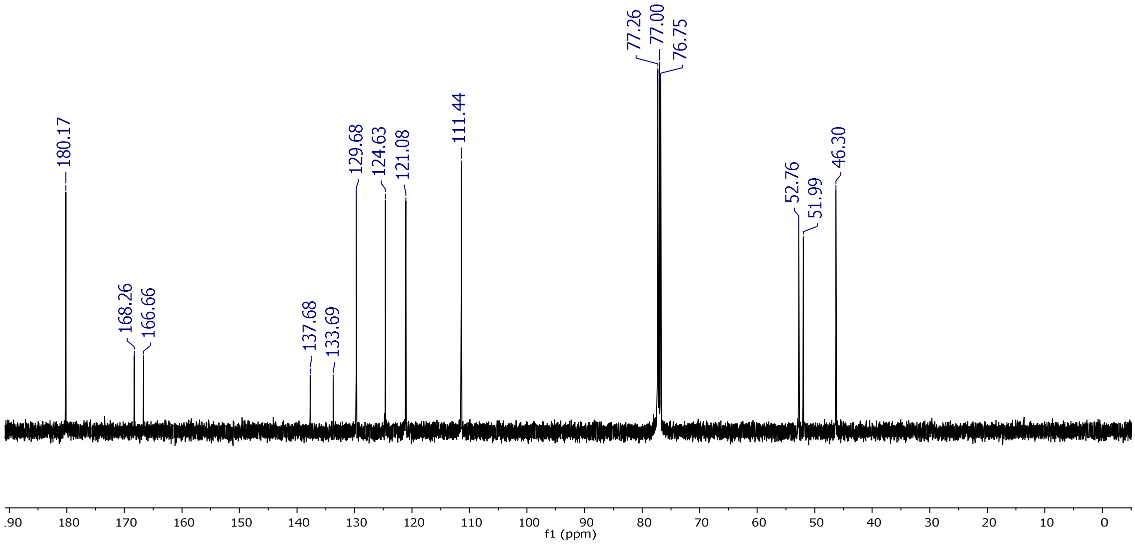

Figure S58. ^{13}C NMR (125 MHz, $CDCl_3$) spectrum of compound **8i**

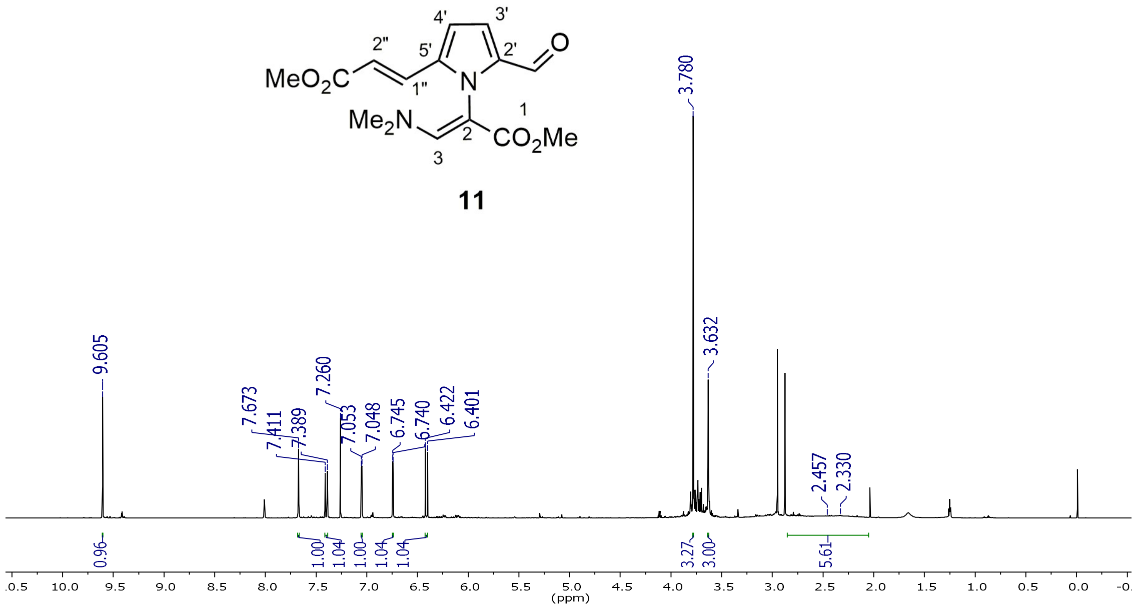

Figure S59. ^{1}H NMR (750 MHz, $CDCl_3$) spectrum of compound **11**

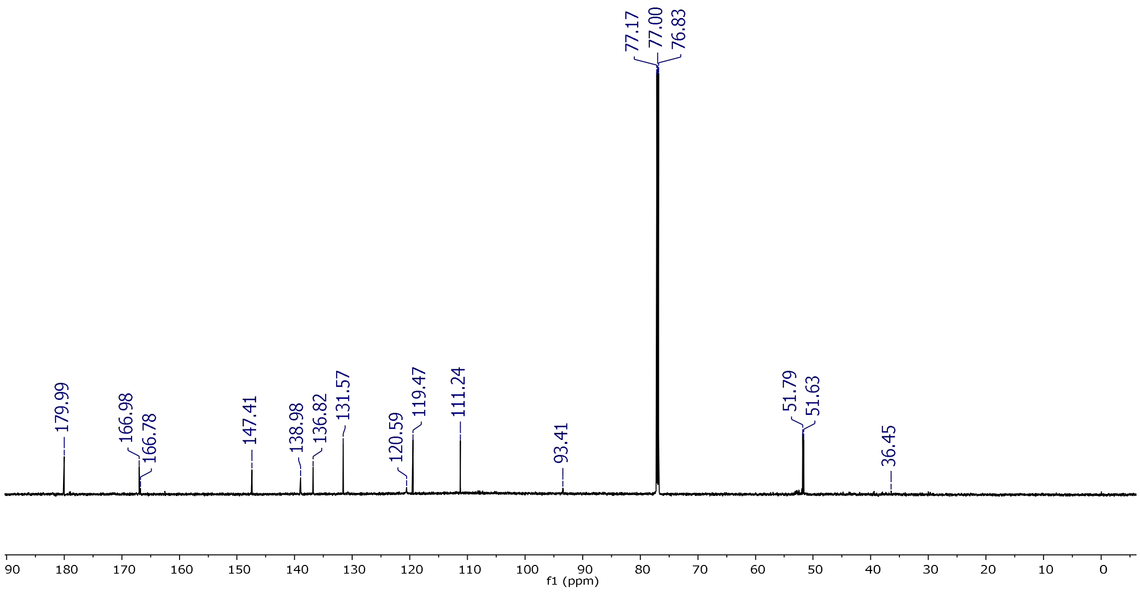

Figure S60. ^{13}C NMR (187.5 MHz, $CDCl_3$) spectrum of compound **11**

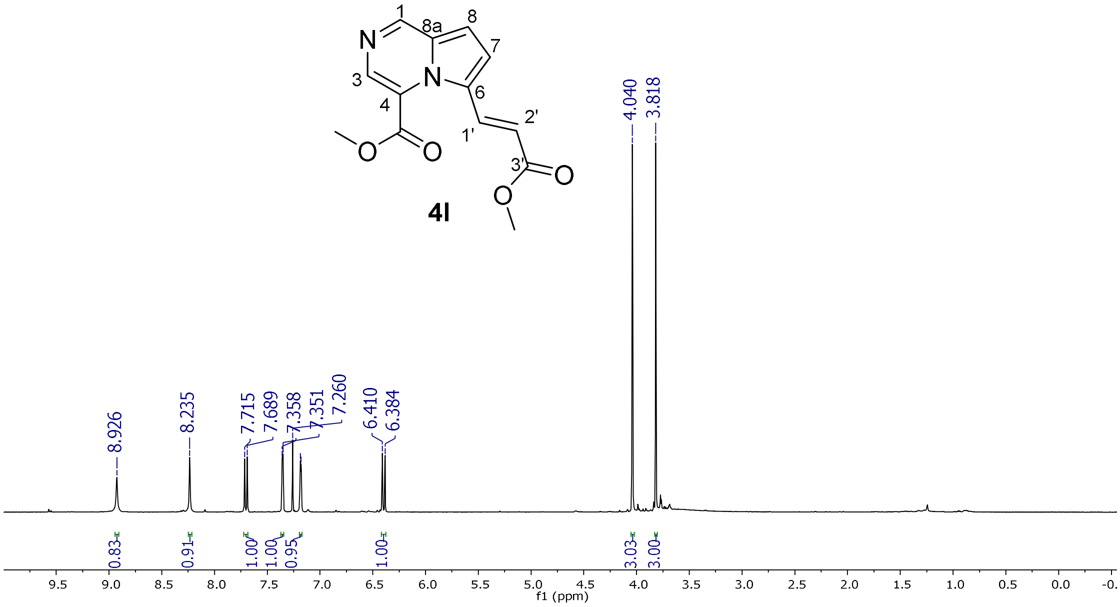

Figure S61. ^{1}H NMR (600 MHz, $CDCl_3$) spectrum of compound **4l**

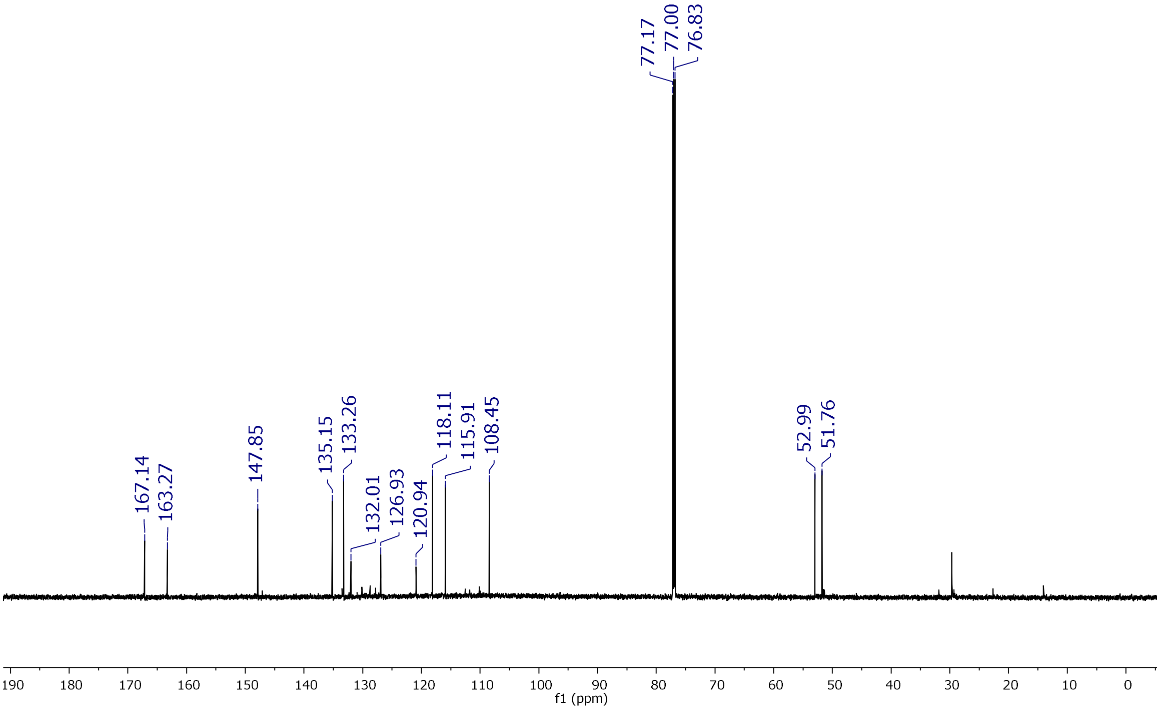

Figure S62. ^{13}C NMR (187.5 MHz, $CDCl_3$) spectrum of compound **4l**

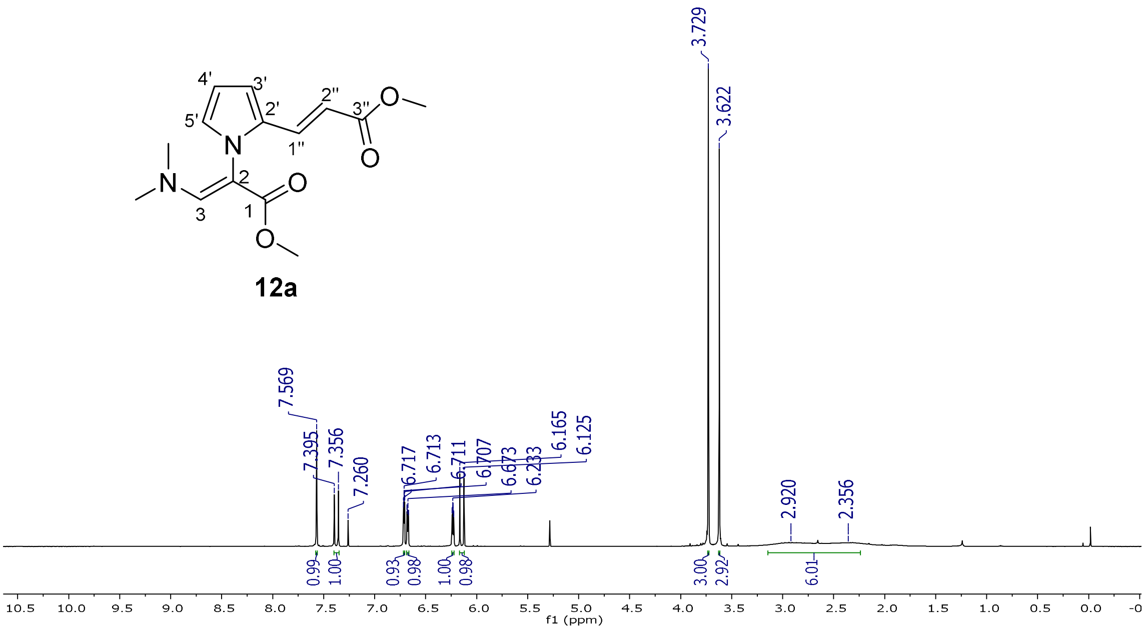

Figure S63. ^{1}H NMR (400 MHz, $CDCl_3$) spectrum of compound **12a**

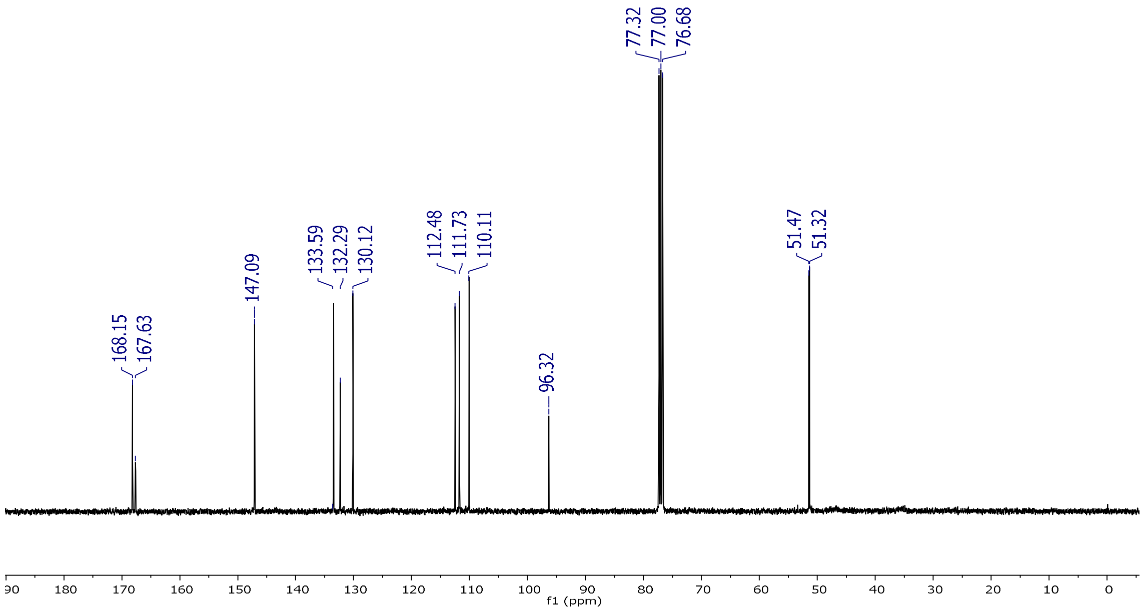

Figure S64. ^{13}C NMR (100 MHz, $CDCl_3$) spectrum of compound **12a**

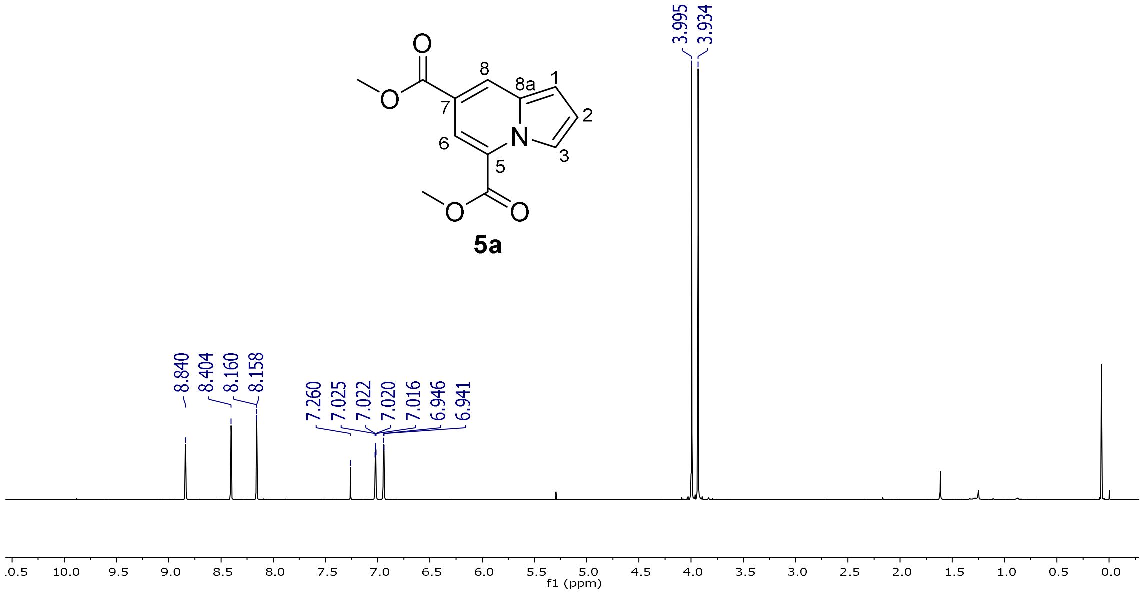

Figure S65. ^{1}H NMR (750 MHz, CDCl$_3$) spectrum of compound **5a**

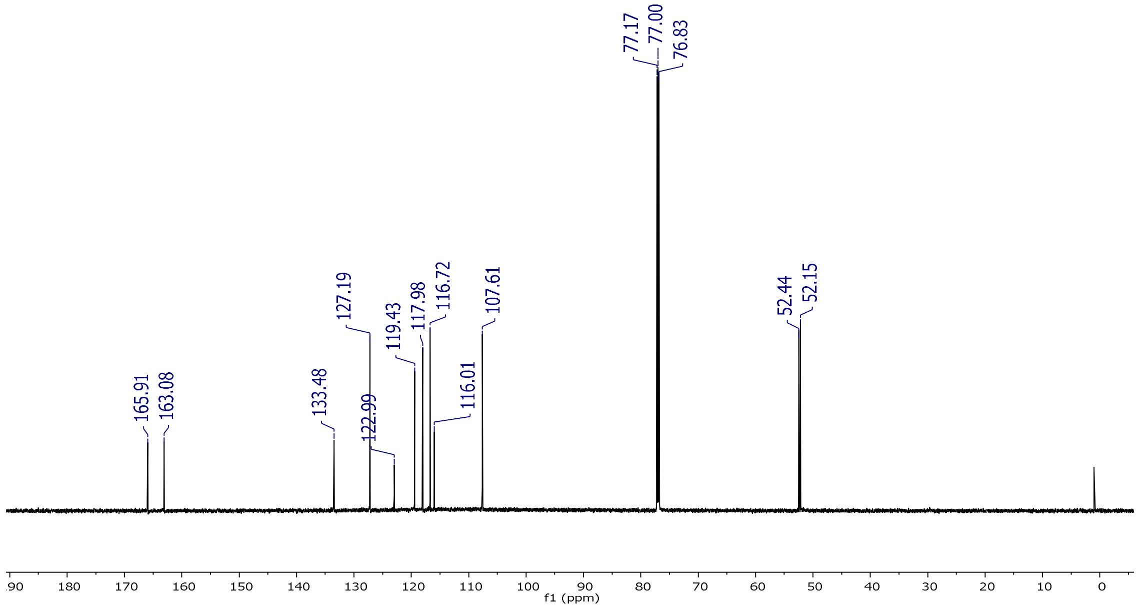

Figure S66. ^{13}C NMR (187.5 MHz, CDCl$_3$) spectrum of compound **5a**

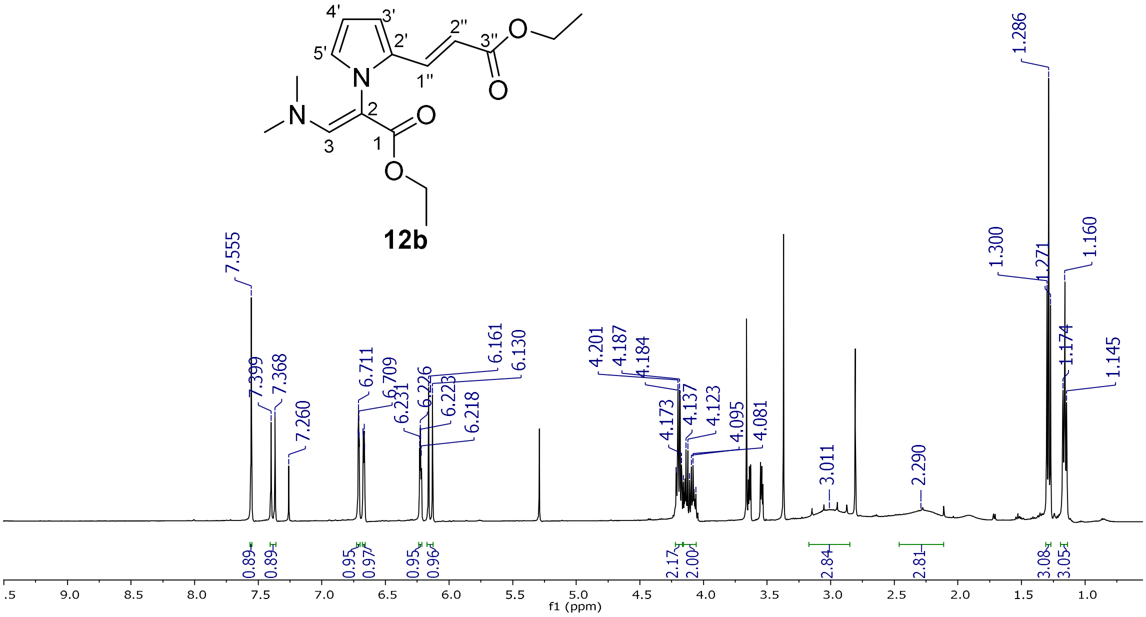

Figure S67. ^{1}H NMR (500 MHz, $CDCl_3$) spectrum of compound **12b**

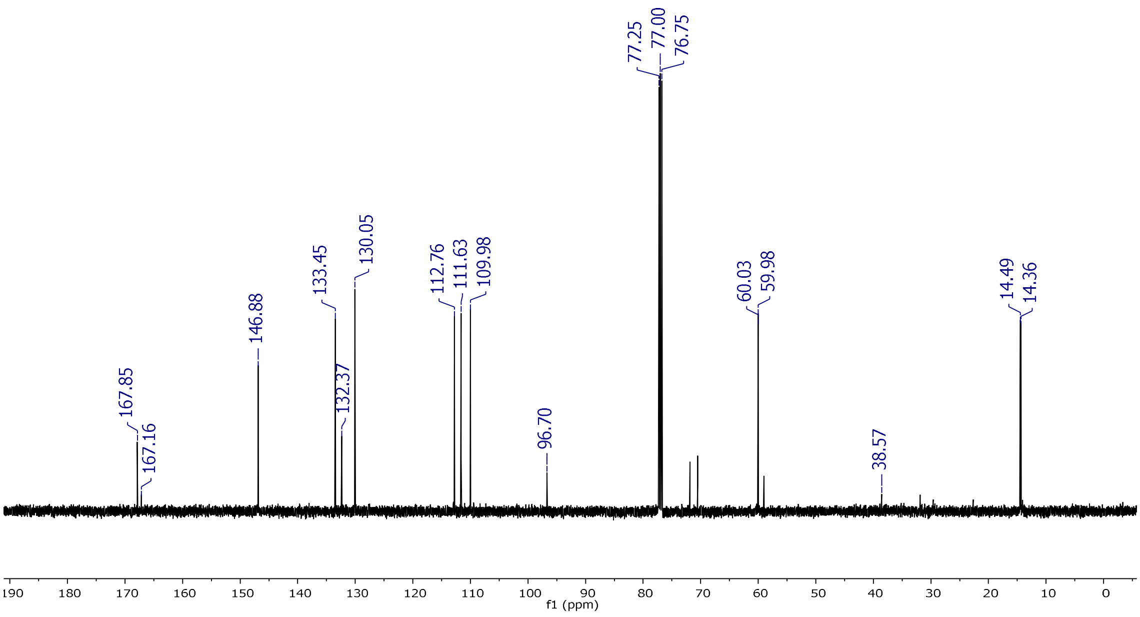

Figure S68. ^{13}C NMR (125 MHz, $CDCl_3$) spectrum of compound **12b**

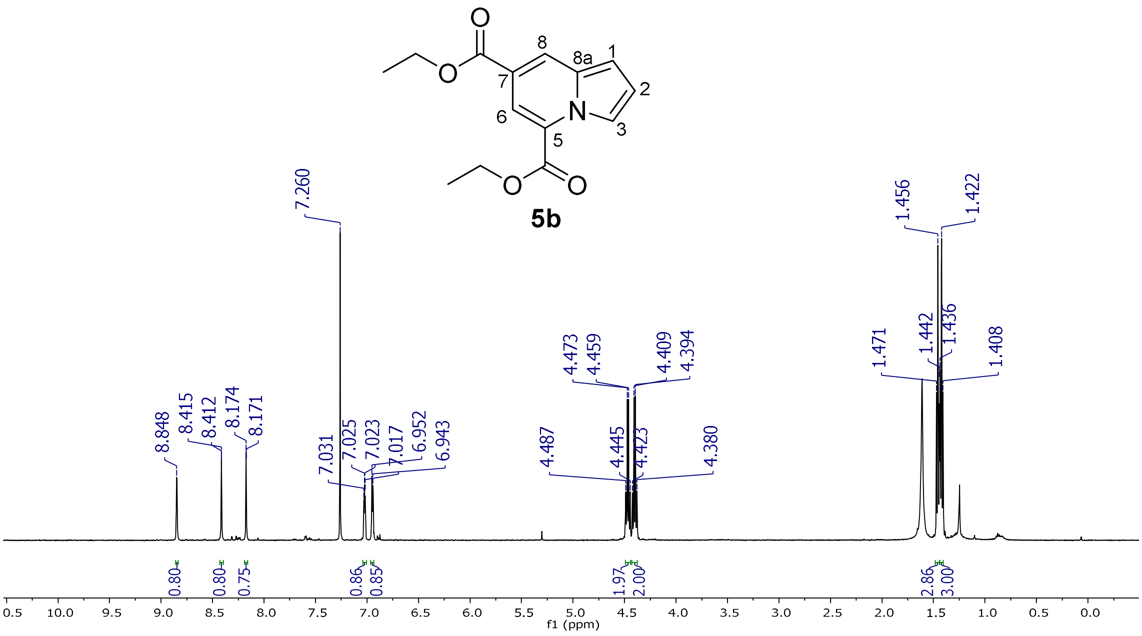

Figure S69. ^{1}H NMR (500 MHz, $CDCl_3$) spectrum of compound **5b**

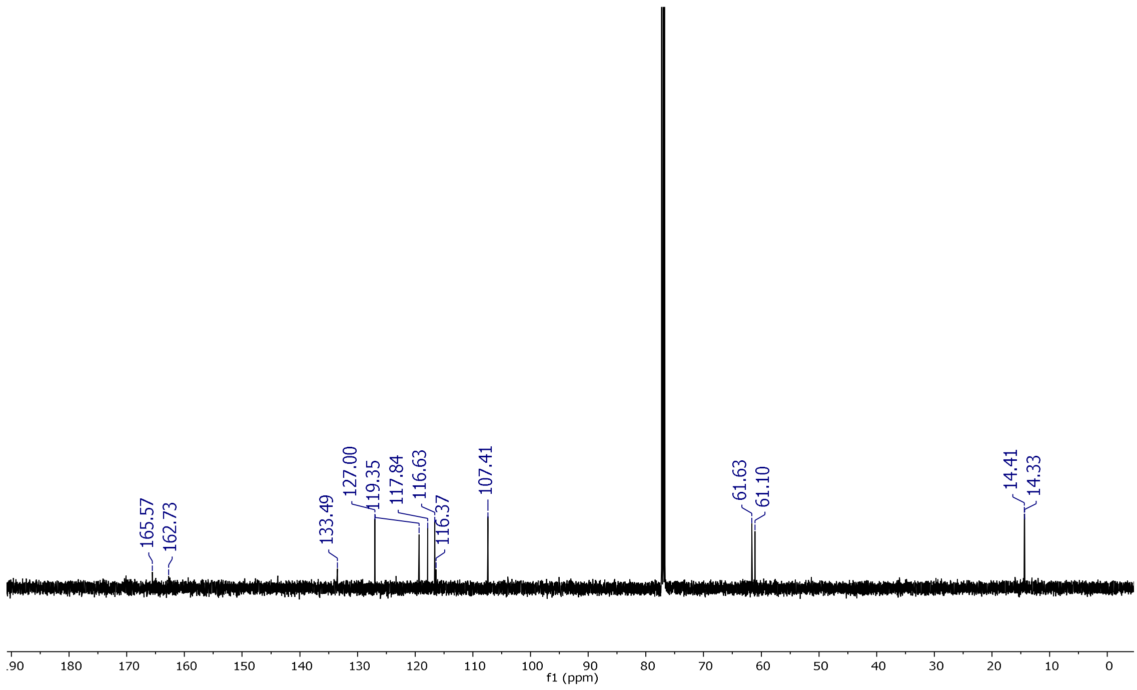

Figure S70. ^{13}C NMR (125 MHz, $CDCl_3$) spectrum of compound **5b**

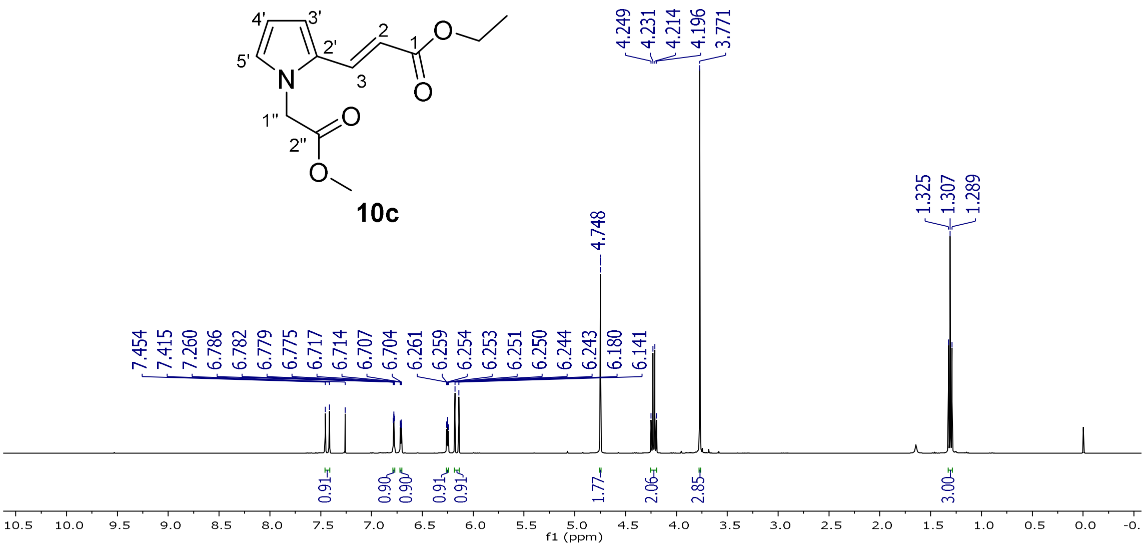

Figure S71. ^{1}H NMR (400 MHz, $CDCl_3$) spectrum of compound **10c**

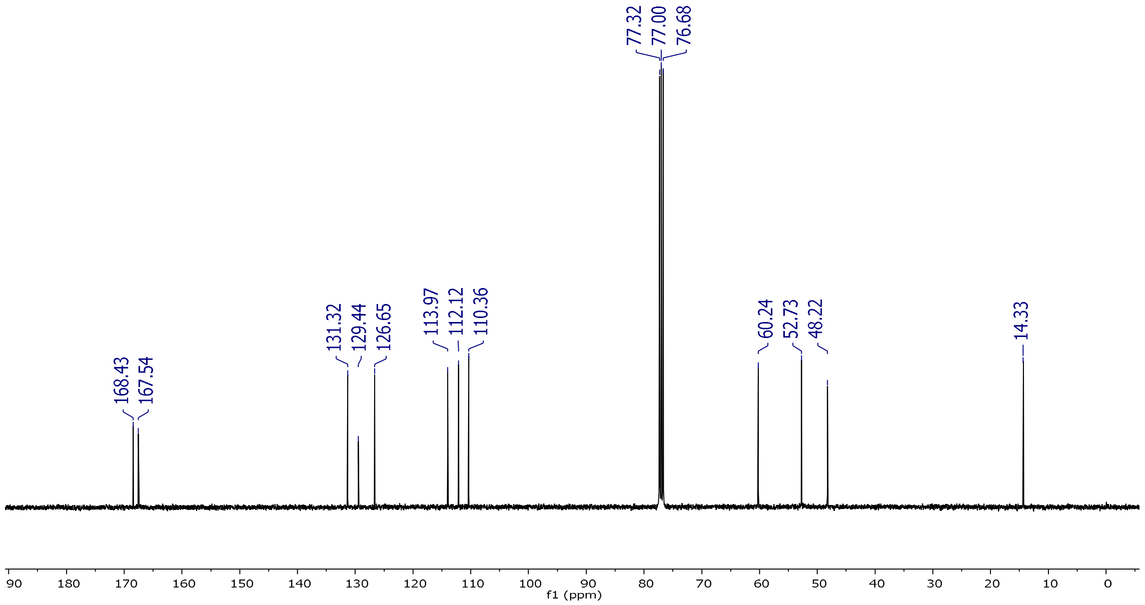

Figure S72. ^{13}C NMR (100 MHz, $CDCl_3$) spectrum of compound **10c**

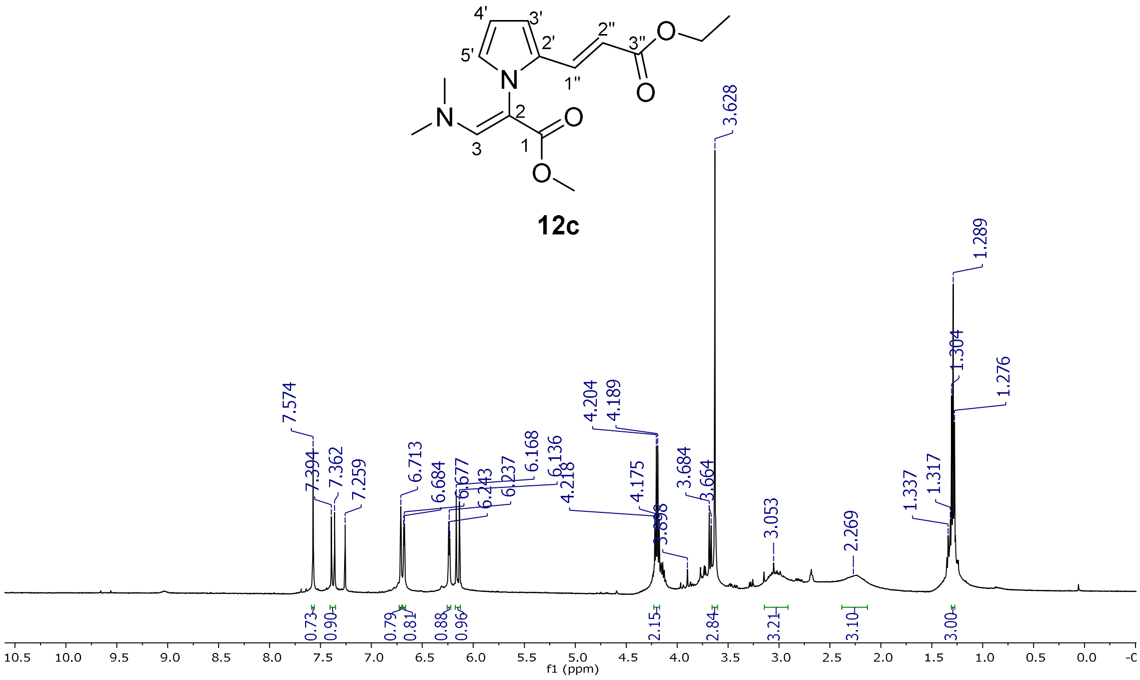

Figure S73. ^{1}H NMR (500 MHz, $CDCl_3$) spectrum of compound **12c**

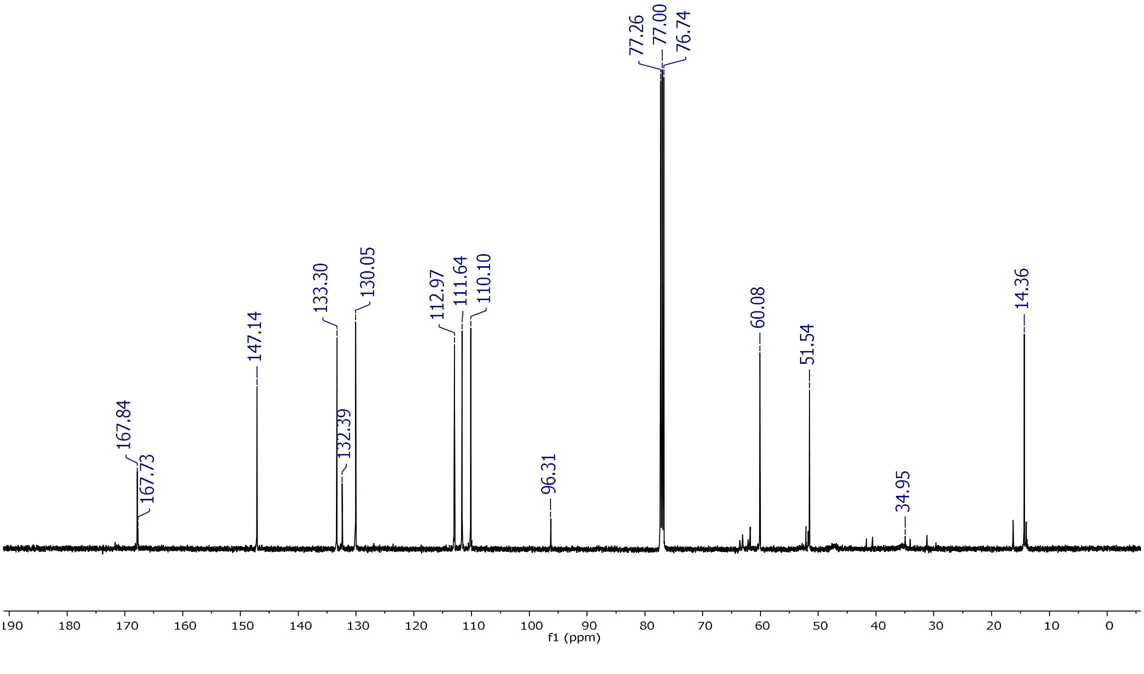

Figure S74. ^{13}C NMR (125 MHz, $CDCl_3$) spectrum of compound **12c**

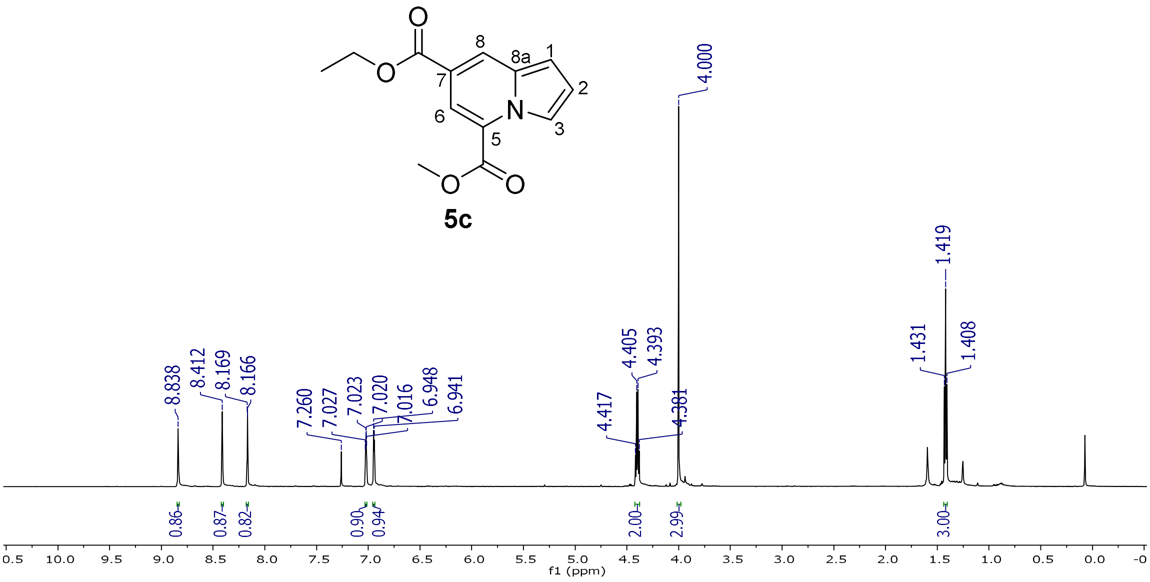

Figure S75. ^{1}H NMR (600 MHz, $CDCl_3$) spectrum of compound **5c**

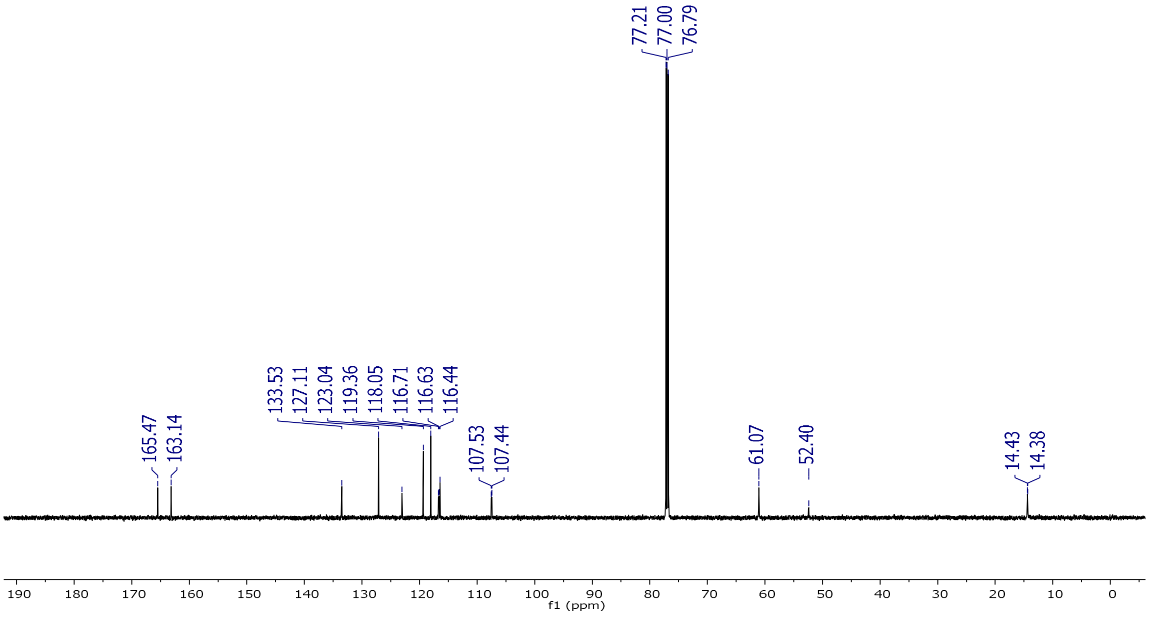

Figure S76. ^{13}C NMR (150 MHz, $CDCl_3$) spectrum of compound **5c**

A

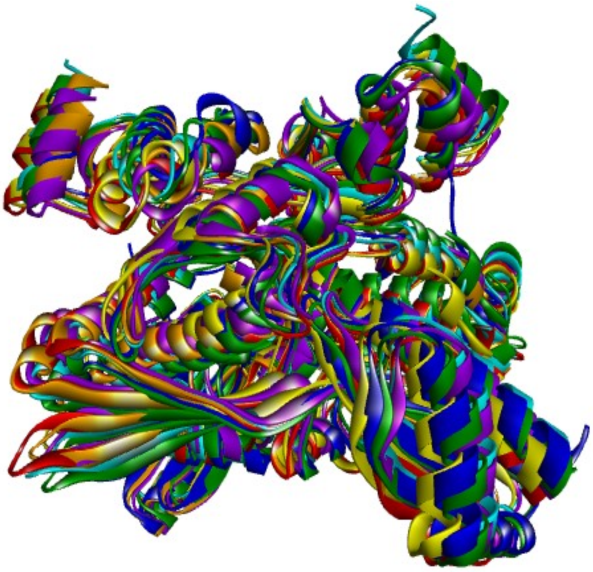

B

Model	% residues in favorable regions
CaHMGR	95.3
CgHMGR	94.5
CdHMGR	93.9
CkHMGR	94.1
CauHMGR	95.0
ChaHMGR	95.6

Figure S77. Acquisition and evaluation of the models of the HMGRs of the *Candida* spp. A) The overlap of CaHMGR (red), CgHMGR (blue), CdHMGR (green), CkHMGR (purple), CauHMGR (orange), ChaHMGR (yellow) and hHMGR (cyan) is shown. B) Results of the percentage (%) of amino acid residues that were located within favorable regions located on a Ramachandran plot (see Figures S78-S83). Values higher than 90% indicate that the model obtained is of excellent quality.

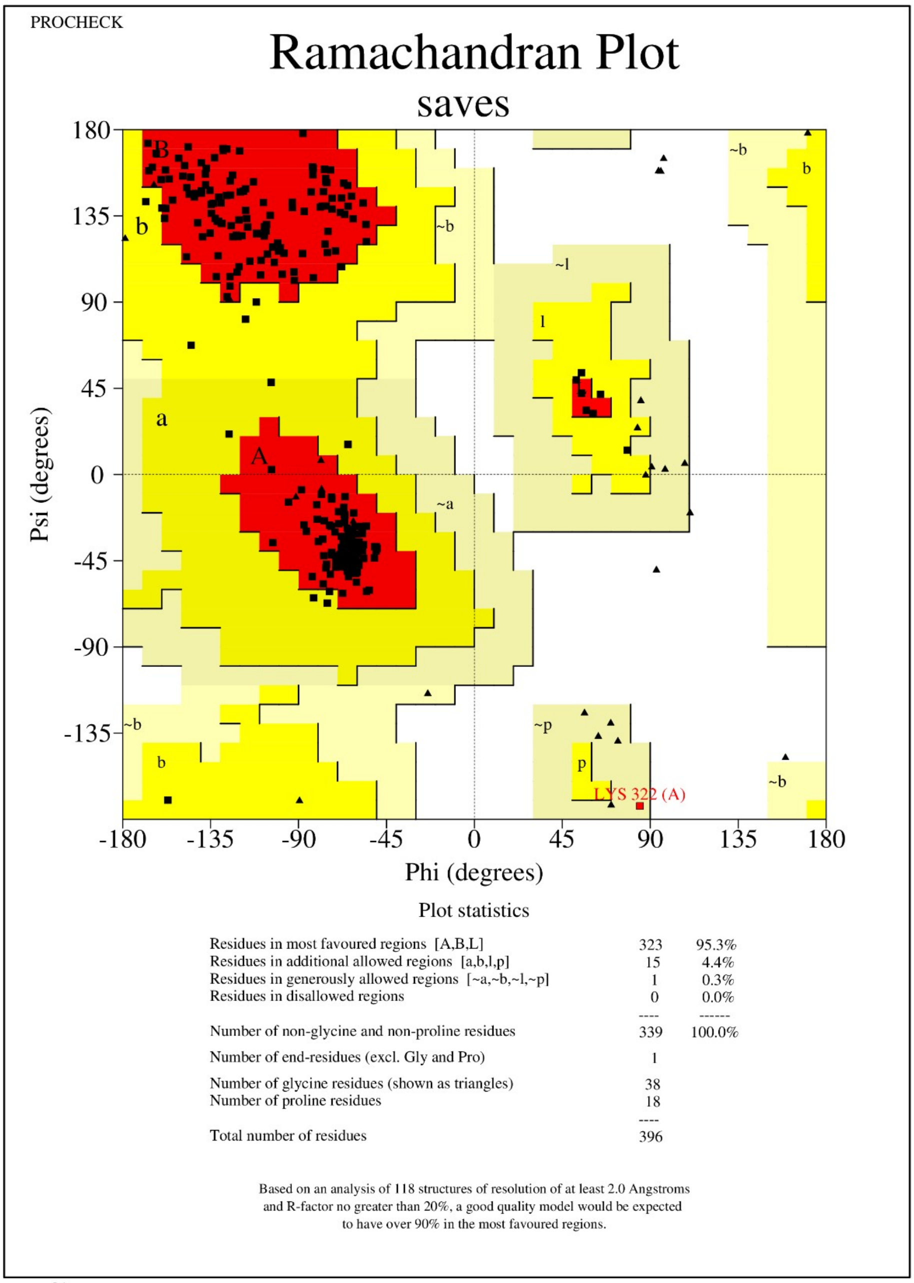

saves_01.ps

Figure S78. Ramachandran plot of CaHMGR. Allowed regions are shown in red, in yellow the less favorable regions are shown, in beige and white are depicted the unfavorable regions.

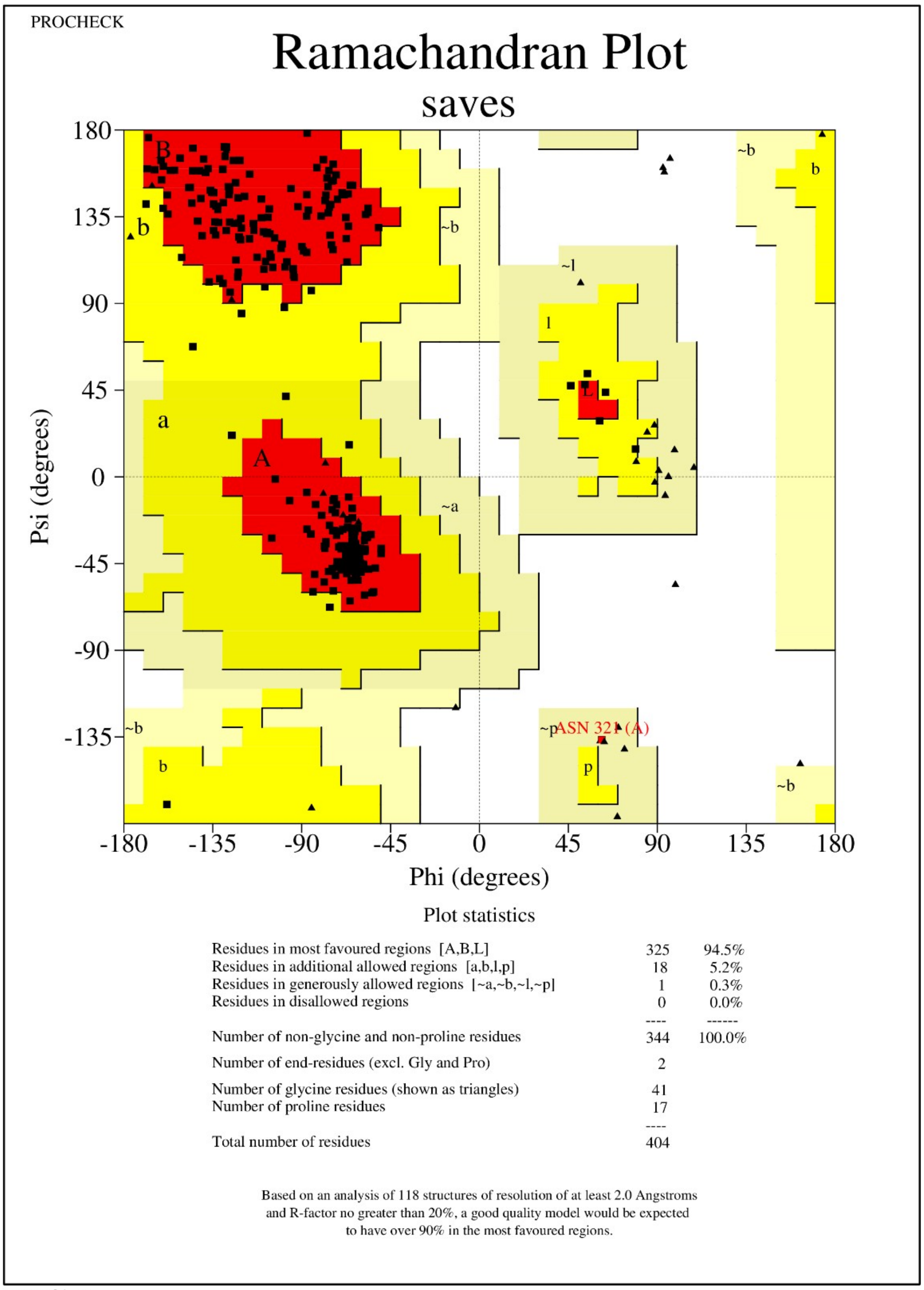

saves_01.ps

Figure S79. Ramachandran plot of CgHMGR. Allowed regions are shown in red, in yellow the less favorable regions are shown, in beige and white are depicted the unfavorable regions.

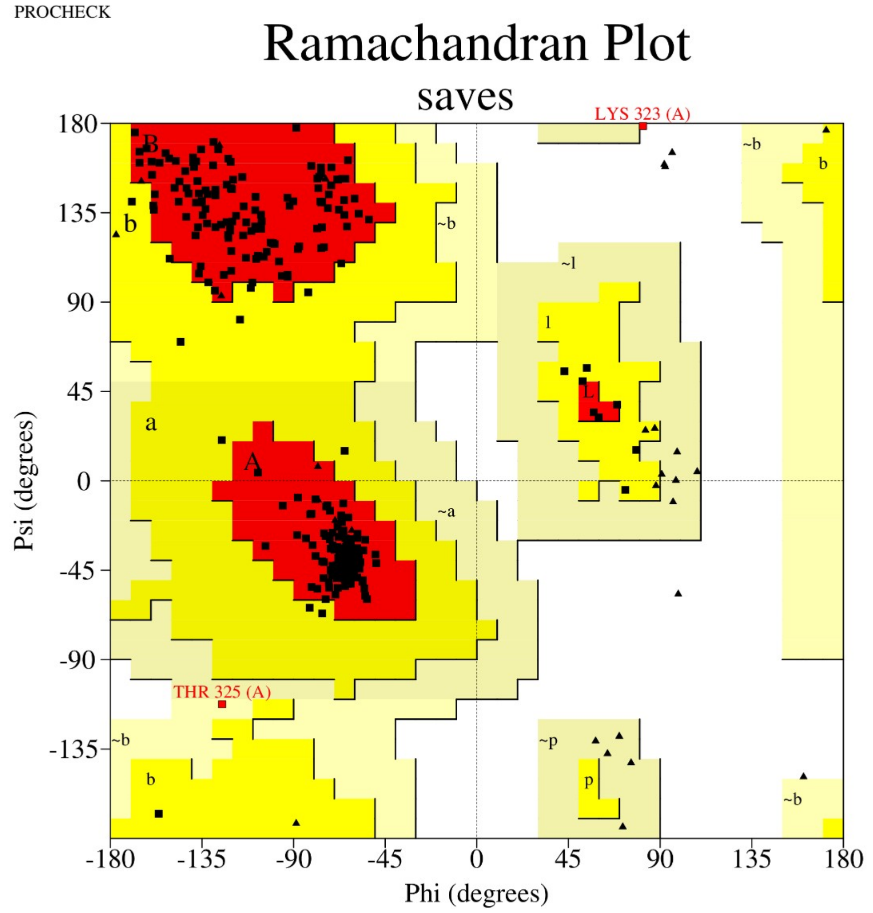

Plot statistics

Residues in most favoured regions [A,B,L]	321	93.9%
Residues in additional allowed regions [a,b,l,p]	19	5.6%
Residues in generously allowed regions [~a,~b,~l,~p]	1	0.3%
Residues in disallowed regions	1	0.3%
	----	------
Number of non-glycine and non-proline residues	342	100.0%
Number of end-residues (excl. Gly and Pro)	2	
Number of glycine residues (shown as triangles)	38	
Number of proline residues	18	

Total number of residues	400	

Based on an analysis of 118 structures of resolution of at least 2.0 Angstroms and R-factor no greater than 20%, a good quality model would be expected to have over 90% in the most favoured regions.

saves_01.ps

Figure S80. Ramachandran plot of CdHMGR. Allowed regions are shown in red, in yellow the less favorable regions are shown, in beige and white are depicted the unfavorable regions.

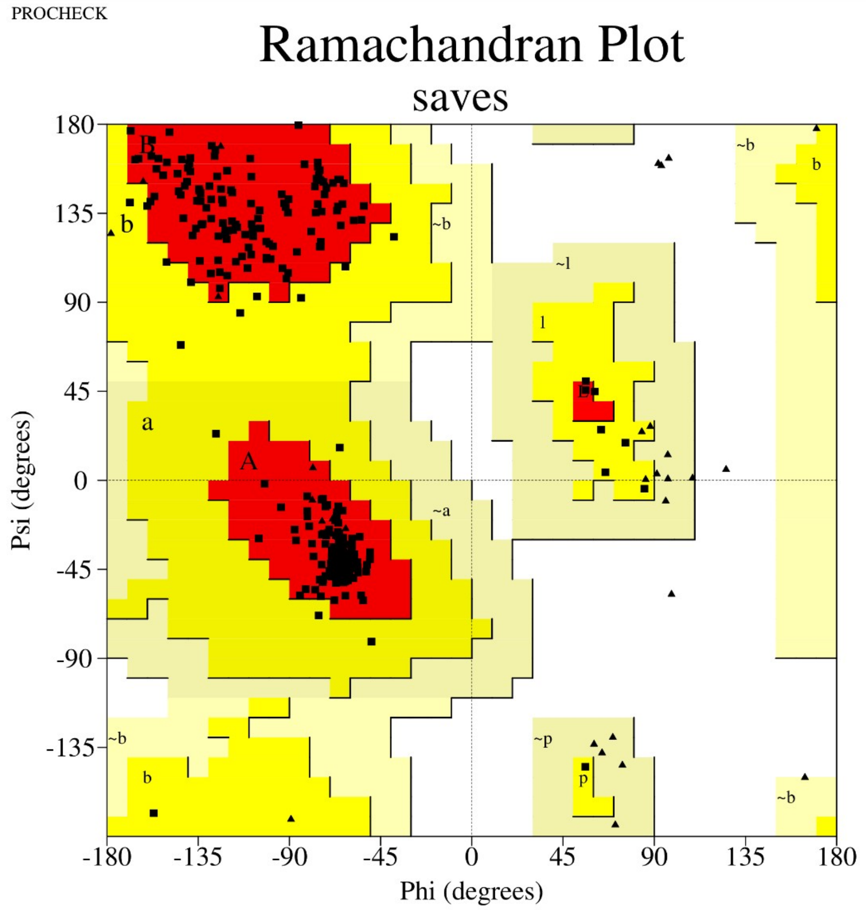

Plot statistics

Residues in most favoured regions [A,B,L]	320	94.1%
Residues in additional allowed regions [a,b,l,p]	20	5.9%
Residues in generously allowed regions [~a,~b,~l,~p]	0	0.0%
Residues in disallowed regions	0	0.0%
	----	------
Number of non-glycine and non-proline residues	340	100.0%
Number of end-residues (excl. Gly and Pro)	2	
Number of glycine residues (shown as triangles)	39	
Number of proline residues	18	

Total number of residues	399	

Based on an analysis of 118 structures of resolution of at least 2.0 Angstroms and R-factor no greater than 20%, a good quality model would be expected to have over 90% in the most favoured regions.

saves_01.ps

Figure S81. Ramachandran plot of CkHMGR. Allowed regions are shown in red, in yellow the less favorable regions are shown, in beige and white are depicted the unfavorable regions.

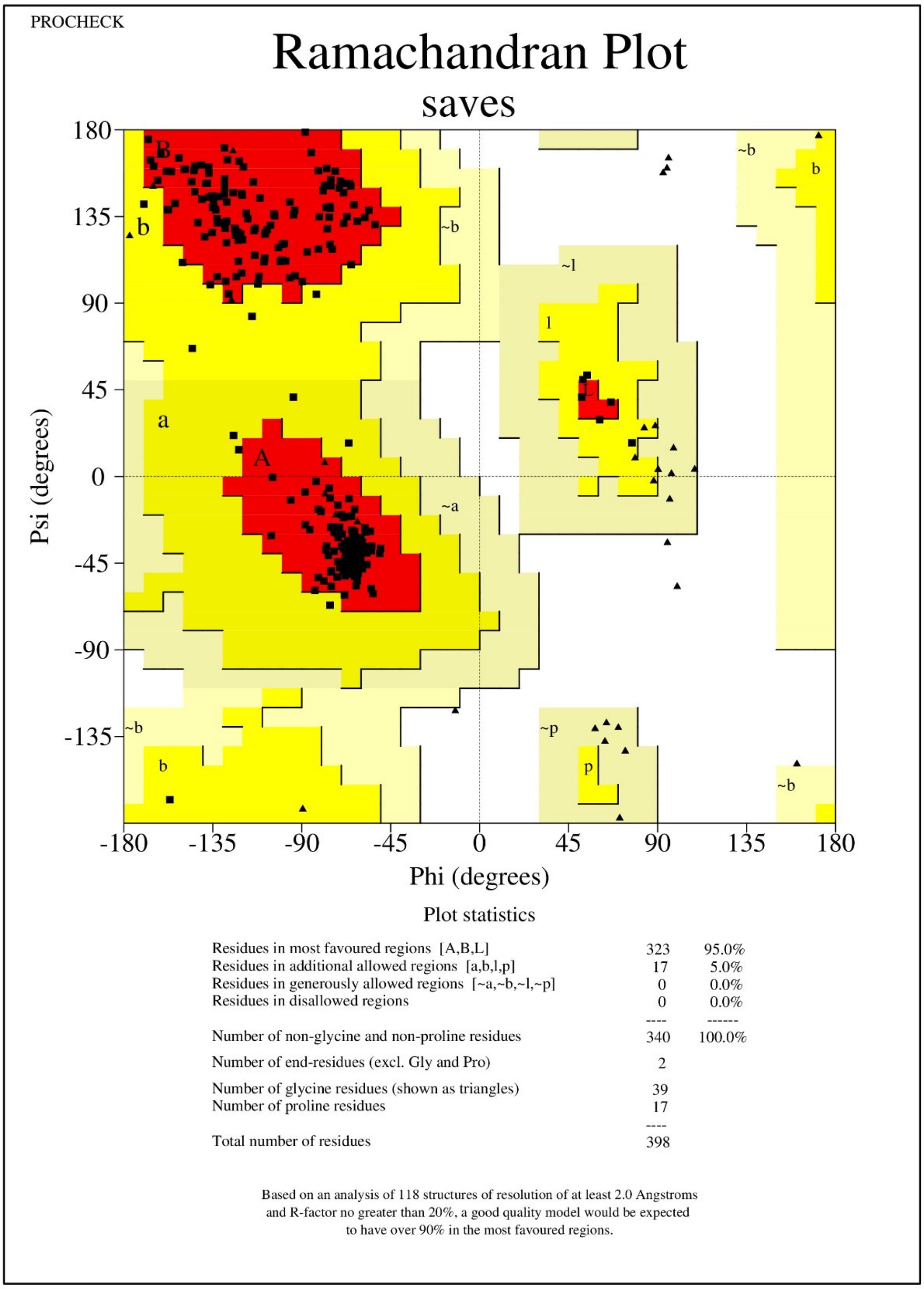

saves_01.ps

Figure S82. Ramachandran plot of CauHMGR. Allowed regions are shown in red, in yellow the less favorable regions are shown, in beige and white are depicted the unfavorable regions.

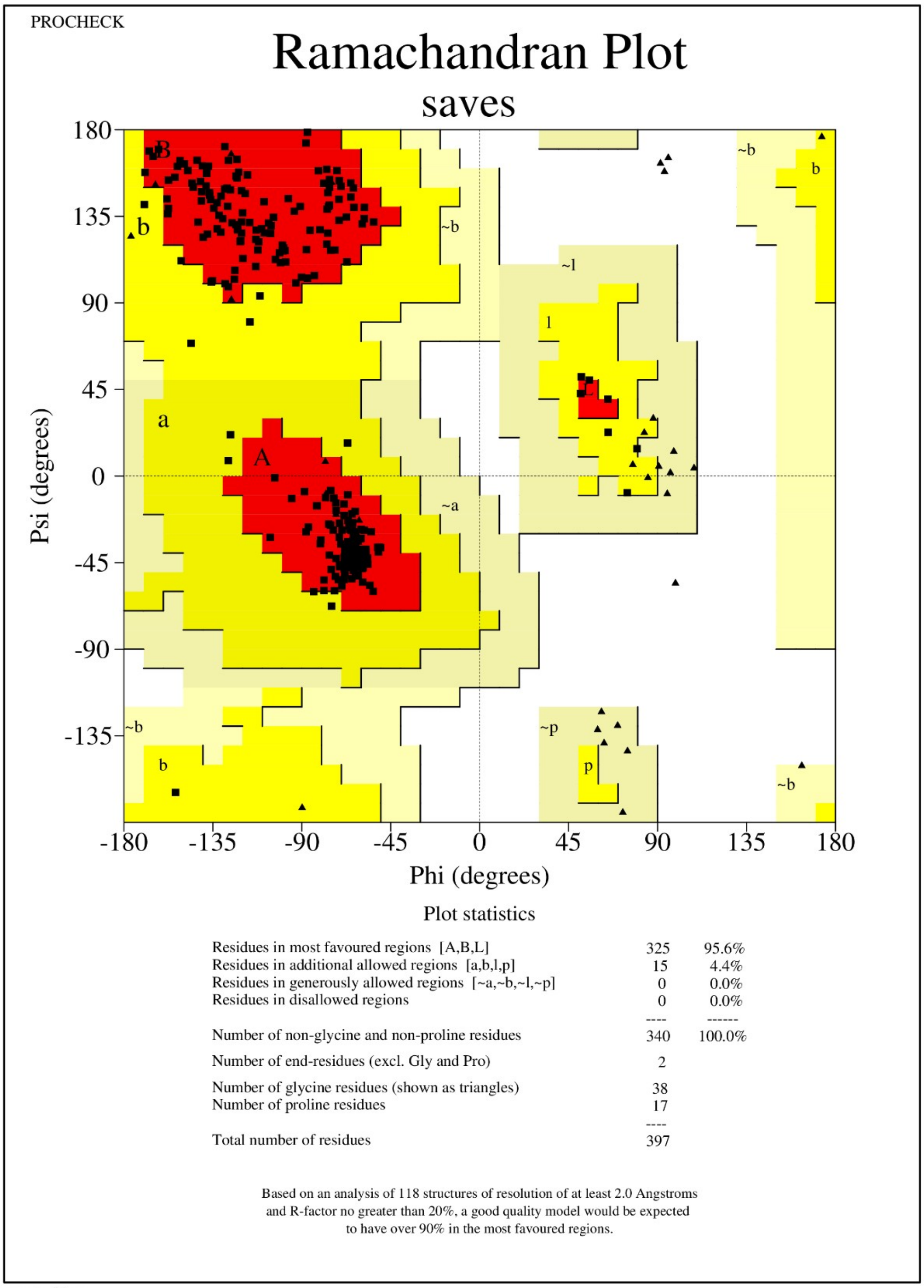

saves_01.ps

Figure S83. Ramachandran plot of ChaHMGR. Allowed regions are shown in red, in yellow the less favorable regions are shown, in beige and white are depicted the unfavorable regions.

Table S1. Data of the interactions of enaminones **1a**, **2a**, and **2c**, pyrrolo[1,2-*a*]pyrazines **4b**, **4g**, and **4l**, indolizine **5a**, *N*-alkyl 2-formylpyrroles **8a**, **8c**, and **8g**, and pyrrole-based alkyl acrylates **10a** and **12a** at the active site of the HMGR enzyme of *C. glabrata* (CgHMGR).

Compound	Enzyme interacting residues with the ligand	Polar interactions	Hydrophobic interactions
simvastatin	Ala59, Thr91, Thr92, Glu93, Leu96, Met189, Met191, Asn192, Met192, Asp226, Lys227, Lys228, Asn291, Thr294, Asp303, Pro304, Gln306, Gly340, Gly341	Thr92, Glu93, Asp303	Met189
atorvastatin	Ala59, Thr91, Thr92, Glu93, Leu96, Met189, Asn192, Lys227, Asn291, Thr294, Gly301, Gln302, Asp303, Pro304, Gly336, Gly339, Gly340, Gly341, Thr342	Al59, Gln302, Asp303, Thr342	Thr92, Met189, Asp303, Pro304, Gly336, Gly339
**1a**	Leu70, Ala90, Thr91, Thr92, Glu93, Leu96, Asn291, Thr294, Leu298, Gln302, Asp303, Pro304	Thr92, Glu93, Gln302	Leu70, Leu298, Pro304
**2a**	Thr92, Glu93, Met189, Asn192, Gly301, Gln302, Asp303, Gly336, Ile338, Gly339, Gly340, Gly341, Thr342	-	Met189, Asp303, Gly339
**2c**	Ala59, Cys60, Leu70, Ala90, Thr91, Thr92, Glu93, Leu96, Lys227, Asn291, Thr294, Ala295, Gly301, Gln302, Asp303, Pro304	Thr92, Lys227	Leu70, Glu93, Thr294, Pro304
**4b**	Leu70, Ala90, Thr91, Thr92, Glu93, Asn291, Thr294, Ala295, Leu298, Asp303, Pro304, Ala305	Thr92, Glu93, Thr294	Leu70, Leu298, Pro304
**4g**	Leu70, Ala90, Thr91, Thr92, Glu93, Thr294, Leu298, Pro304	Thr92, Ala90	Leu70, Leu298, Pro304
**4l**	Leu70, Ala90, Thr91, Thr92, Glu93, Asn291, Thr294, Leu298, Gln302, Asp303, Pro304	Thr294	Asp303, Pro304
**5a**	Leu70, Ala90, Thr91, Thr92, Glu93, Leu96, Asn291, Thr294, Ala295, Leu298, Asp303, Pro304	Glu93, Asn291, Thr294	Leu70, Leu298, Pro304
**8a**	Ser220, Asn222, Asp226, Lys227, Lys228, Lys271, Ala287, His288, Asn291	Asp226, Lys271	Lys227, Lys228
**8c**	Leu70, Ala90, Thr91, Thr92, Glu93, Leu96, Asn291, Thr294, Ala295, Leu298, Asp303, Pro304, Ala305	Thr92, Asp303, Ala305	Leu70, Leu298, Pro304
**8g**	Met189, Gly301, Gln302, Asp303, Gln306, Glu334, Gly336, Thr337, Ile338, Gly339, Gly340	-	Met189, Gly339
**10a**	Leu70, Ala90, Thr92, Thr294, Ala295, Leu298, Gly301, Gln302, Pro304	Thr294, Tyr297, Pro304	Leu70, Leu298
**12a**	Ala59, Cys60, Thr92, Glu93, Met189, Gly301, Gln302, Asp303, Pro304, Gly336,	Thr92, Asp303, Gly336, Gly339	Asp303

	Gly339, Gly340, Gly341, Thr342		

Table S2. Data of the interactions of enaminones **1a**, **2a**, and **2c**, pyrrolo[1,2-*a*]pyrazines **4b**, **4g**, and **4l**, indolizine **5a**, *N*-alkyl 2-formylpyrroles **8a**, **8c**, and **8g**, and pyrrole-based alkyl acrylates **10a** and **12a** at the active site of the HMGR enzyme of *C. auris* (CauHMGR).

Compound	Enzyme interacting residues with the ligand	Polar interactions	Hydrophobic interactions
simvastatin	Ala63, Cys64, Leu73, Ala93, Thr94, Thr95, Glu96, Leu99, Met193, Lys229, Asn292, Thr295, Ala296, Leu299, Gly303, Gln304, Asp305, Pro306, Gly341, Gly342, Gly343	Cys64, Ala93, Thr94, Lys229, Asn292, Thr295, Asp305	Pro306
atorvastatin	Thr94, Thr95, Glu96, Met193, Met195, Asn196, Aps228, Lys229, lys230, Ala288, Asn292, Thr295, Gly303, Gln304, Asp305, Ile340, Gly341, Gly342, Gly343	-	Thr95, Glu96, Asp228, Lys229, Asp305, Gly342
**1a**	Leu73, Ala93, Thr94, Thr95, Glu96, Thr295, Ala296, Leu299, Leu300, Gln304, Asp305, Pro306	Ala93, Thr95, Thr295, Gln304	Asp305, Pro306
**2a**	Met71, Leu73, Ala93, Thr94, Thr95, Glu96, Thr295, Leu299, Tyr299, Leu300, Gly303, Gln304, Pro306, Gly343, Gln349	Thr95, Glu96	Met71, Leu299, Leu300, Pro306
**2c**	Leu73, Ala93, Thr94, Thr95, Glu96, Met193, Lys229, Asn292, Thr295, Leu299, Gly303, Gln304, Asp305, Pro306, Ala307, Gly341, Gly343, Thr344	Thr94, Thr95, Glu96, Lys229, Thr295, Gly303, Gln304, Asp305, Gly341, Thr344	Leu73, Gln304, Pro306
**4b**	Leu73, Ala93, Thr94, Thr95, Glu96, Leu99, Asn292, Thr295, Ala296, Leu299, Gln304, Asp305	Thr95, Glu96, Asn292	Ala296, Leu299, Asp305
**4g**	Leu73, Ala93, Thr94, Thr95, Glu96, Leu99, Asn292, Thr295, Ala296, Leu299	Thr94, Thr95, Asn292	Ala296, Leu299
**4l**	Leu73, Ala93, Thr94, Thr95, Glu96, Leu99, Asn292, Thr295, Ala296, Leu299, Leu300, Gly303, Gln304, Asp305, Pro306, Gly343, Gln349	Thr95, Asn292, Gln304	Ala296, Leu299, Asp305, Pro306
**5a**	Met71, Leu73, Ala93, Thr94, Thr95, Thr295, Ala296, Leu299, Tyr299, Leu300,	Ala93, Thr95, Thr295, Gly303	Asp305, Pro306

	Gly303, Gln304, Asp305, pro306, Gly343, Gln349		
**8a**	Leu73, Ala93, Thr94, Thr95, Glu96, Lys229, Asn292, Thr295, Ala296, Leu299, Asp305, Pro306, Ala307	Thr95, Thr295	Asp305, Ala307
**8c**	Cys64, Leu73, Ala93, Thr94, Thr95, Glu96, Leu99, Lys229, Asn292, Thr295, Ala296, Leu299, Gln304, Asp305, Pro306, Ala307	Glu96, Thr94, Thr95	Thr295, Asp305, Pro306, Ala307
**8g**	Cys64, Ala93, Thr94, Thr95, Glu96, Leu99, Lys229, Asn292, Thr295, Ala296, Leu299, Aps305, Ala307	Thr94, Glu96, Thr295	Ala296, Leu299
**10a**	Leu74, Ala94, Thr95, Thr96, Glu97, Thr296, Tyr298, Leu299, Leu300, Gly302, Gln303, Pro305, Pro306, Gly342, Gln348	Ala94, Thr95, Thr96, Leu299, Gly302, Gly342, Gln348	-
**12a**	Ala63, Ala93, Leu73, Thr94, Thr95, Glu96, Thr295, Leu299, Gly303, Gln304, Asp305, Pro306, Gly341, Gly342, Gly343	Ala63, Thr94, Thr95, Gly303, Gln304, Gly343	Pro306

***C. glabrata*-simvastatin**

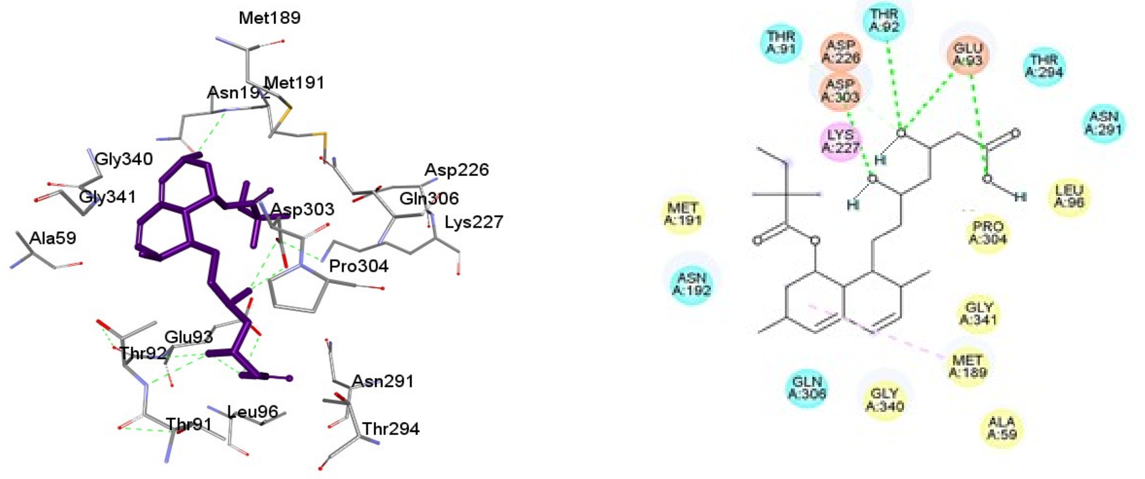

***C. glabrata*-atorvastatin**

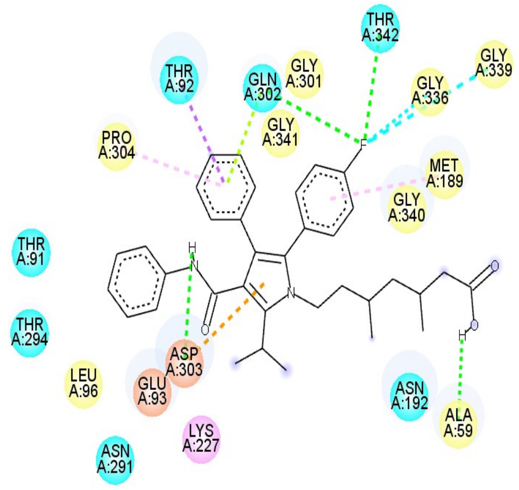

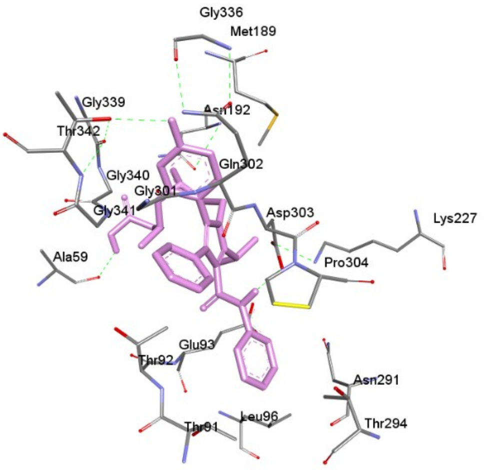

C. glabrata-**1a**

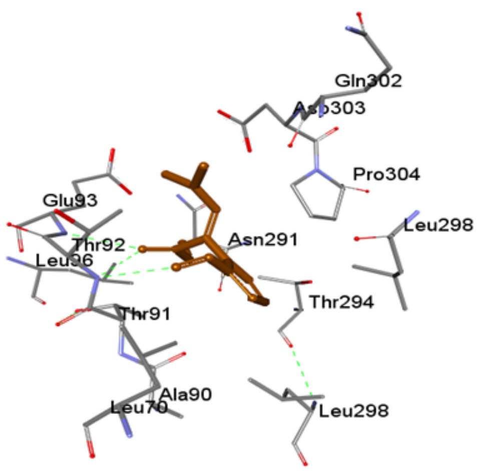

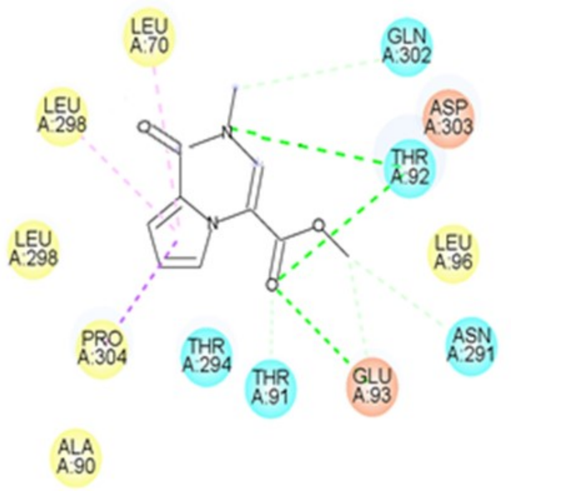

C. glabrata-**2ª**

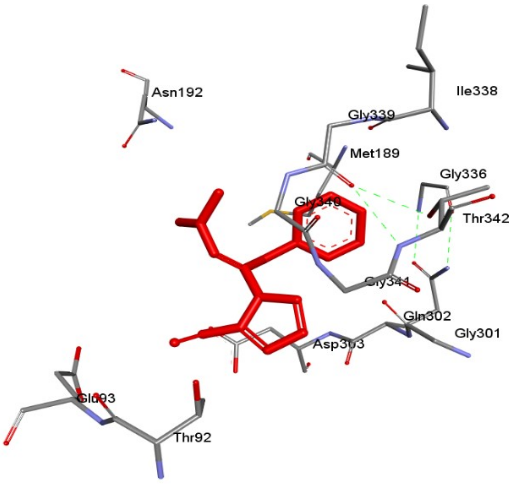

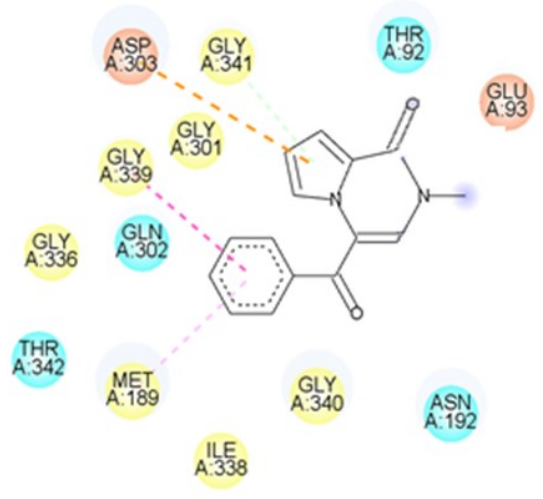

C. glabrata-**2c**

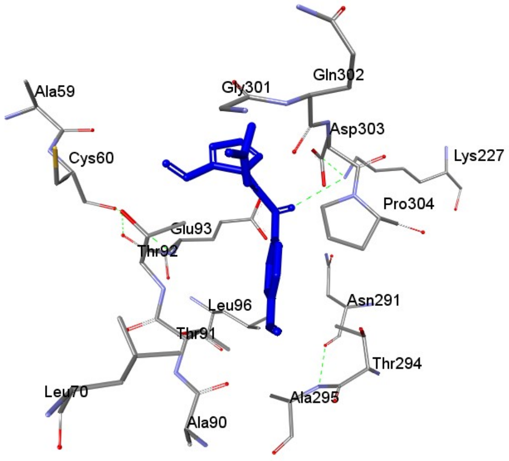

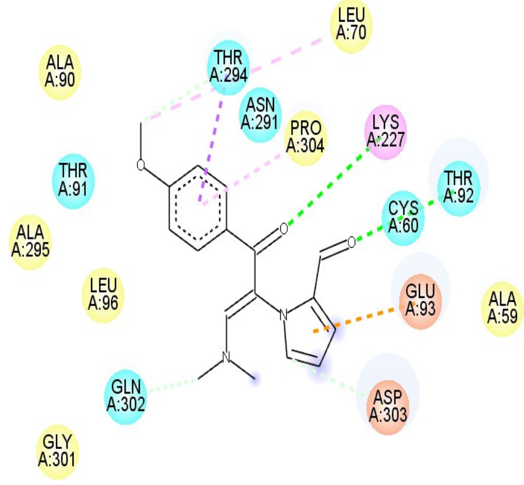

C. glabrata-**4b**

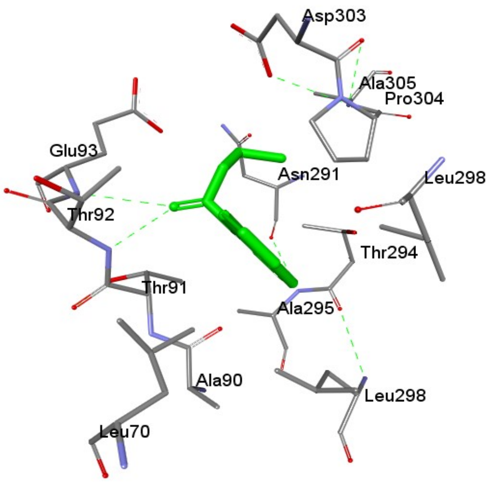

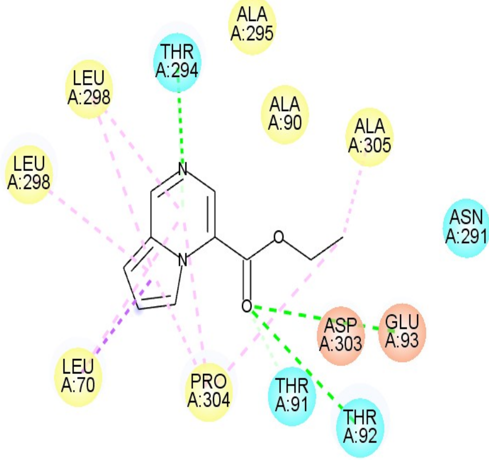

C. glabrata-**4g**

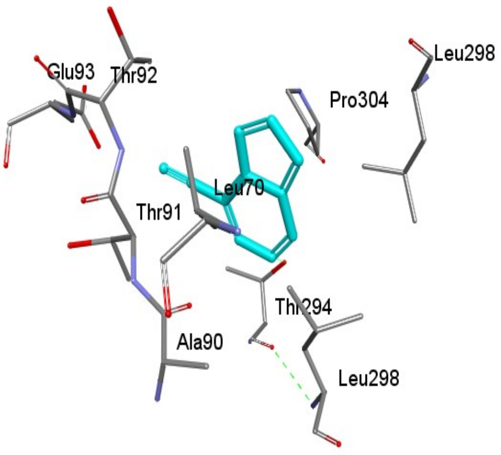

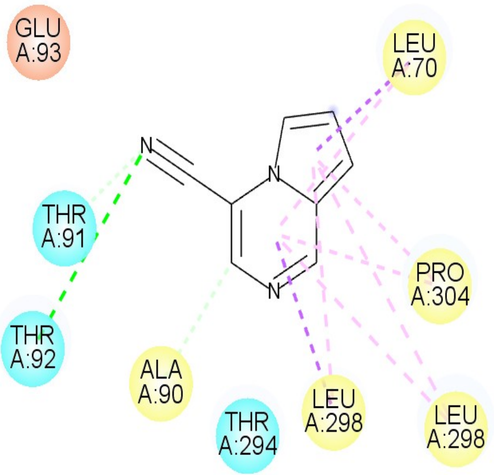

C. glabrata-**4I**

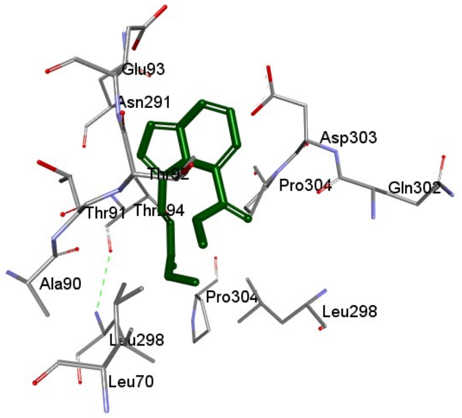

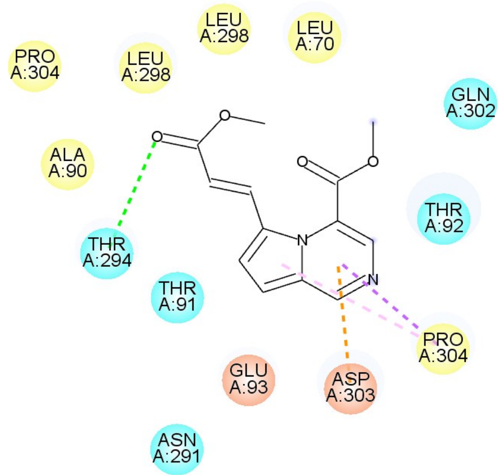

C. glabrata-**5a**

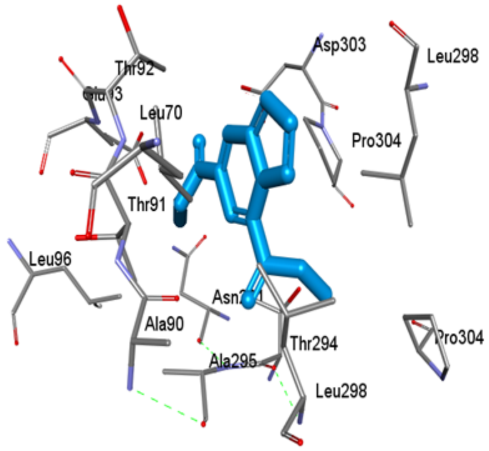

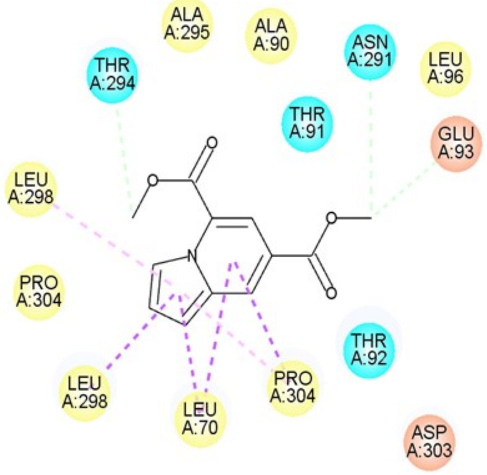

C. glabrata-**8a**

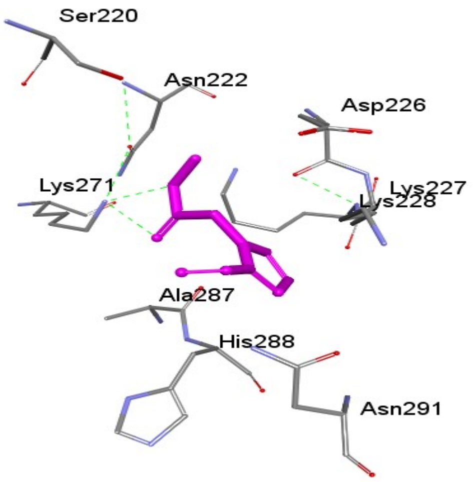

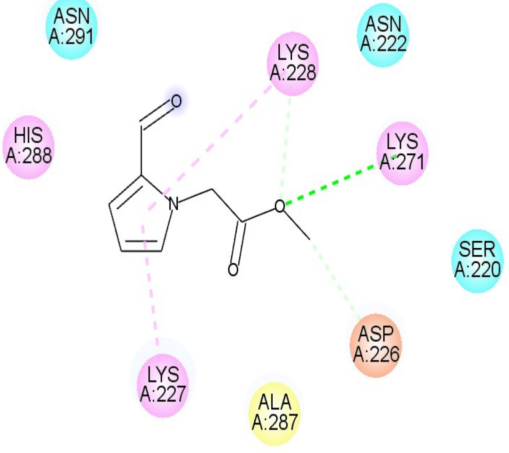

C. glabrata-8c

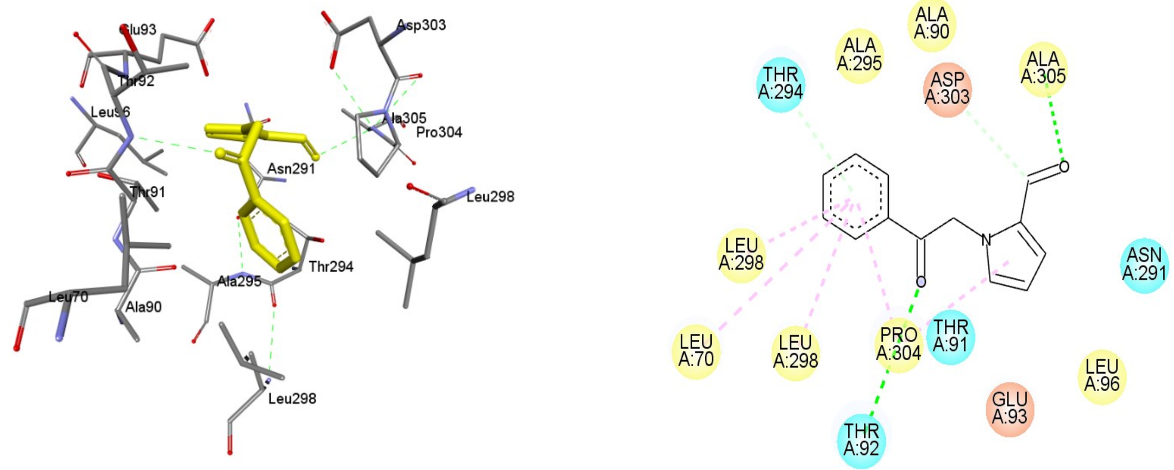

C. glabrata-8g

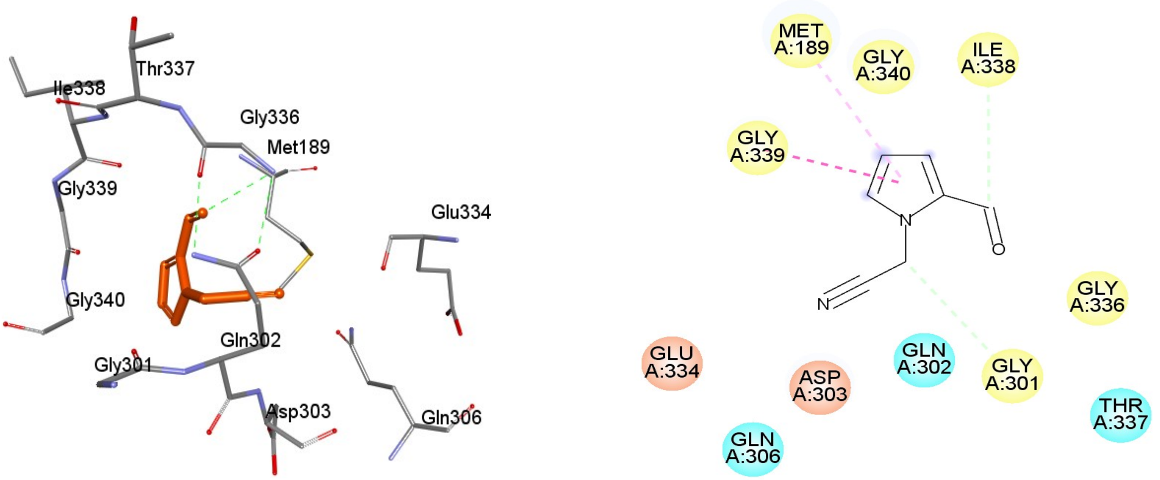

C. glabrata-**10a**

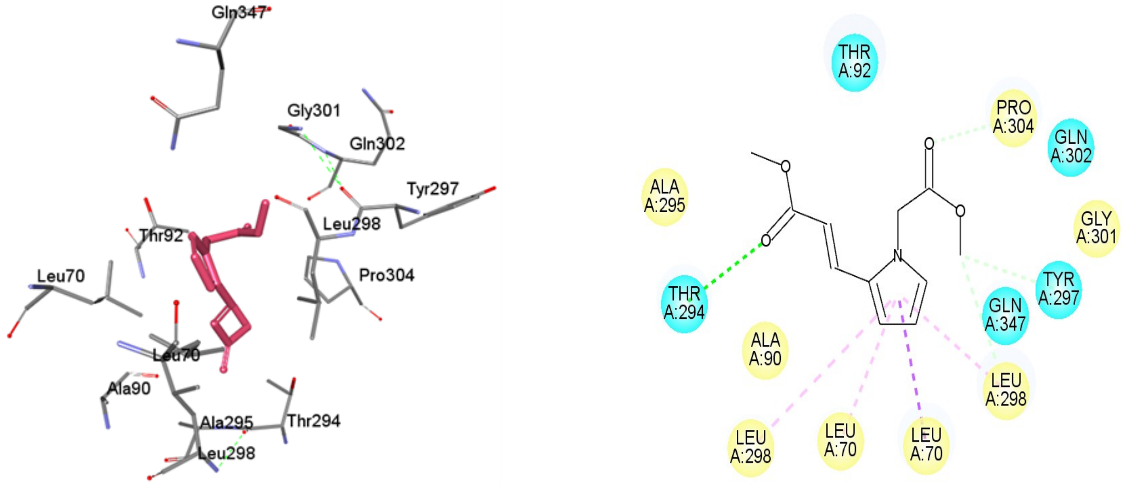

C. glabrata-**12a**

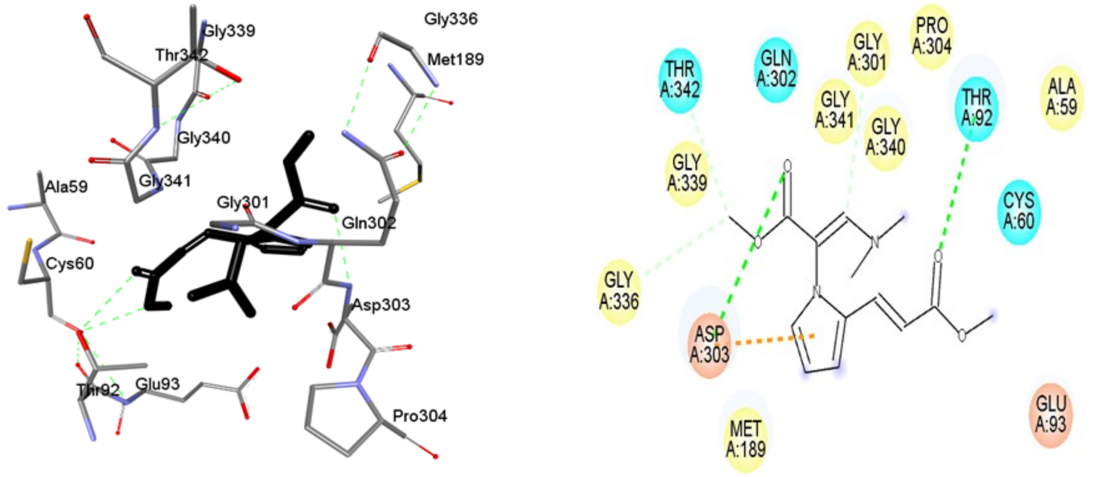

Figure S84. Representation of the interactions between the active site of HMGR of *C. glabrata* and simvastatin, atorvastatin, and enaminone-containing pyrroles **1a**, **2a**, and **2c**, pyrrolo[1,2-*a*]pyrazines **4b**, **4g**, and **4l**, indolizine **5a**, *N*-alkyl 2-formylpyrroles **8a**, **8c**, and **8g**, and pyrrole-based alkyl acrylates **10a** and **12a**. The 3D model shows the amino acid residues involved in ligand binding to the active site of the enzyme. In the 2D model, the following interactions are depicted with dotted lines: conventional hydrogen bond (dark green), carbon hydrogen (light green), π-sigma (purple), π-π T-shaped and π-π stacked (fuchsia), π-alkyl and alkyl (pink), π-anion (orange) and π-cation (yellow). The amino acids were represented in pink circles (for basic amino acids), orange (for acid), cyan (for polars), and yellow (for non-polar).

***C. auris*-simvastatin**

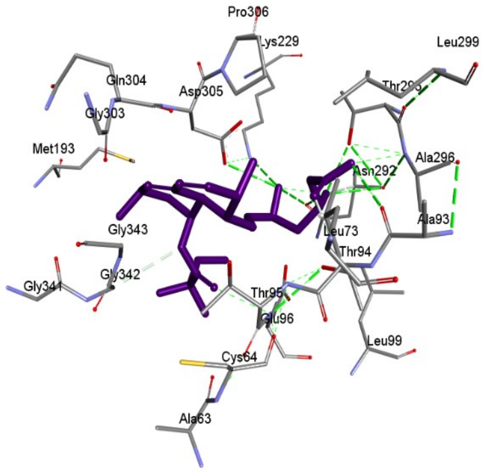

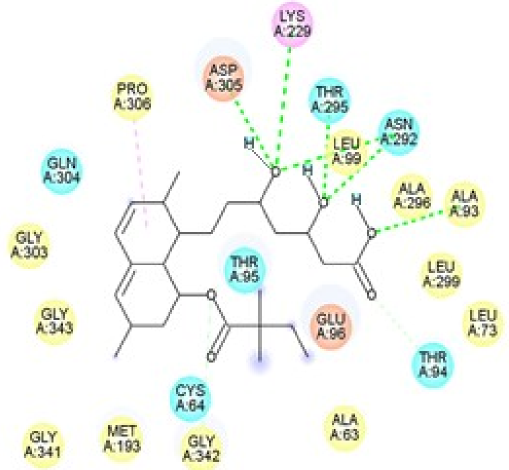

***C. auris*-atorvastatin**

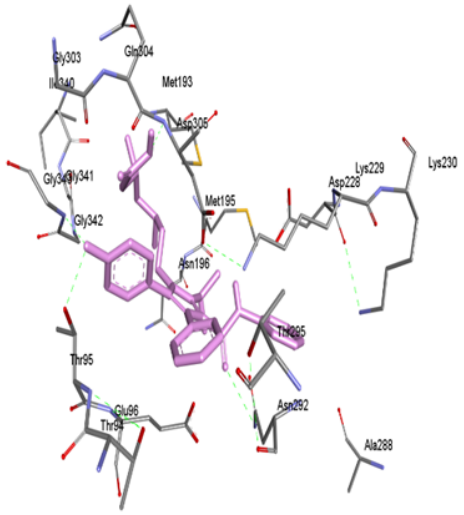

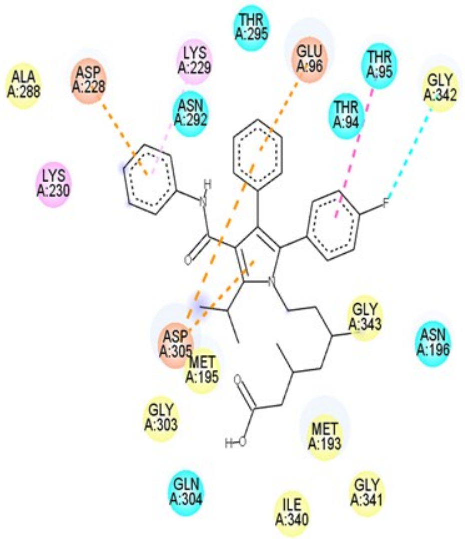

C. auris-**1a**

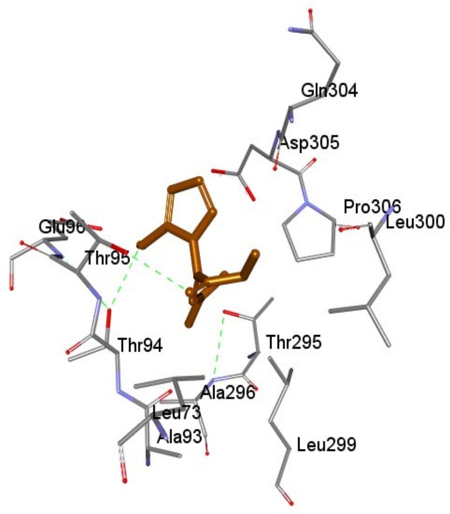

C. auris-**2a**

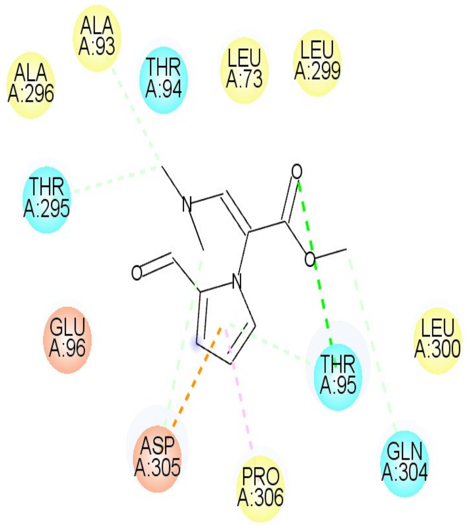

C. auris-**2a**

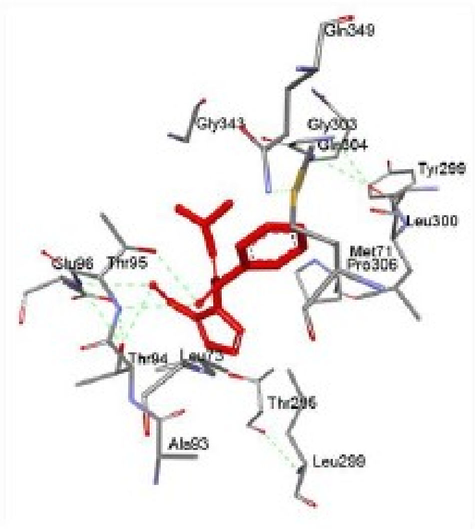

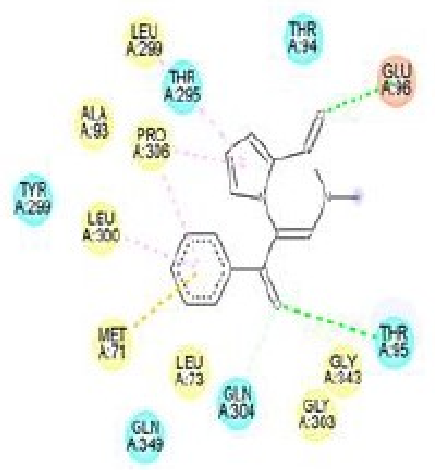

C. auris-**2c**

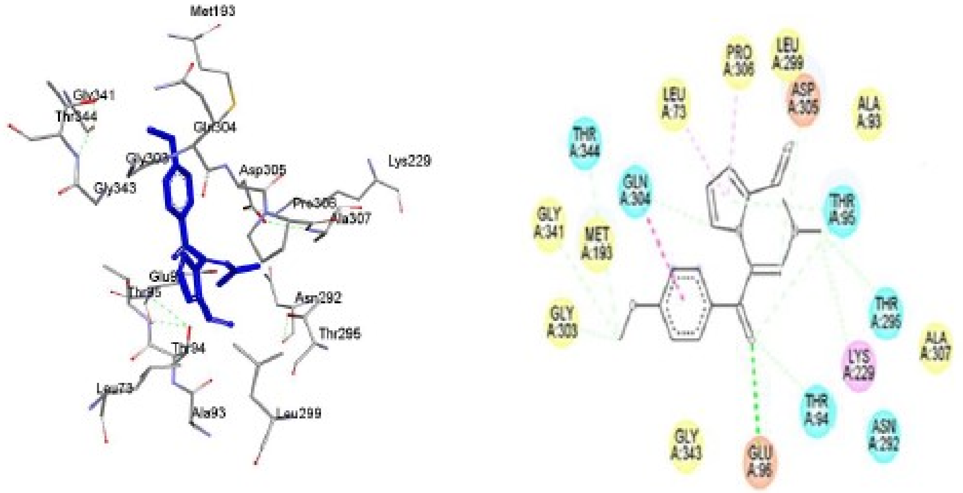

C. auris-**4b**

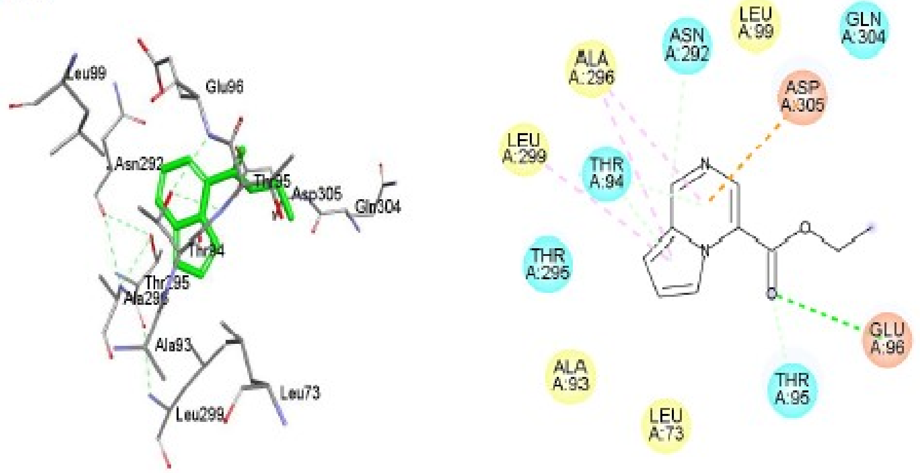

C. auris-**4g**

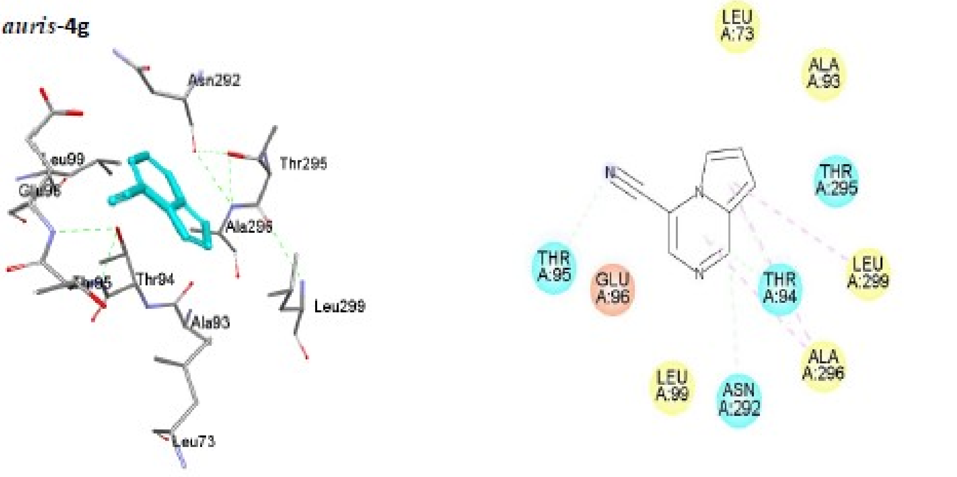

***C. auris*-4I**

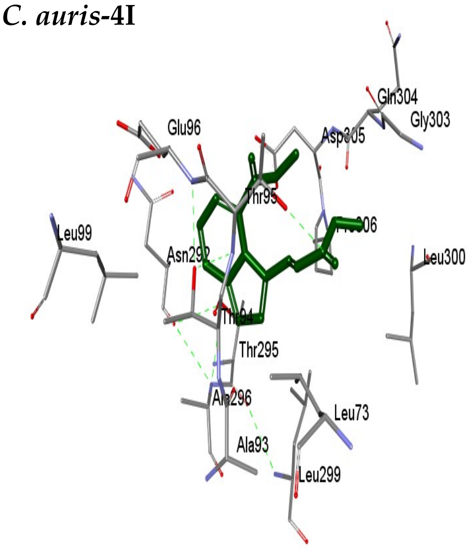

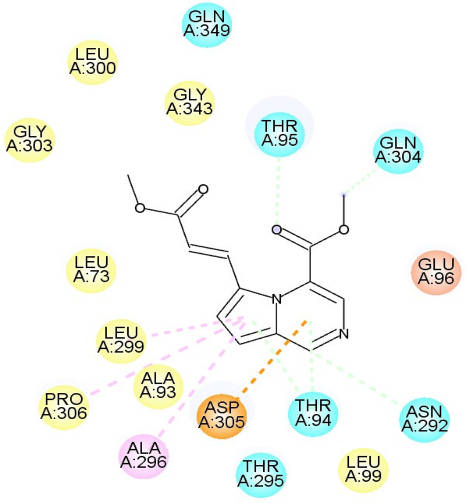

***C. auris*-5a**

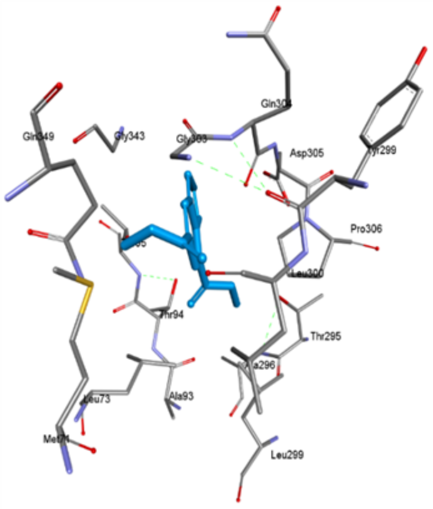

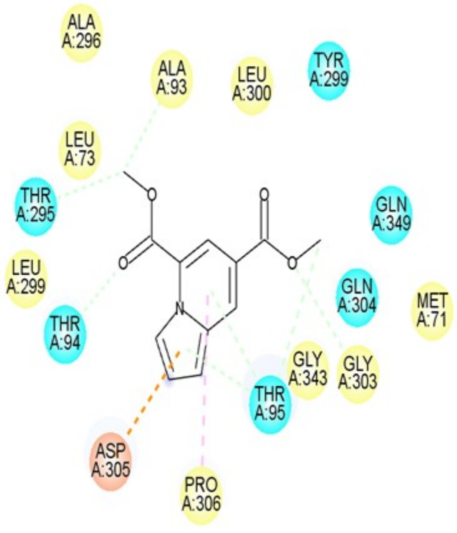

***C. auris*-8a**

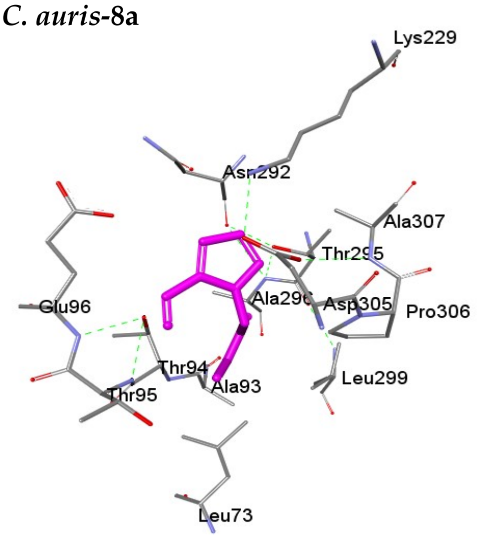

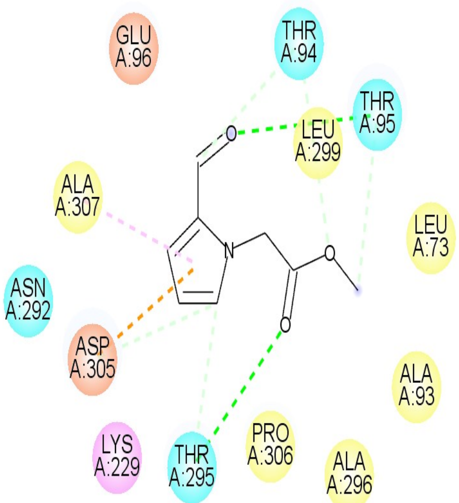

C. auris-**8c**

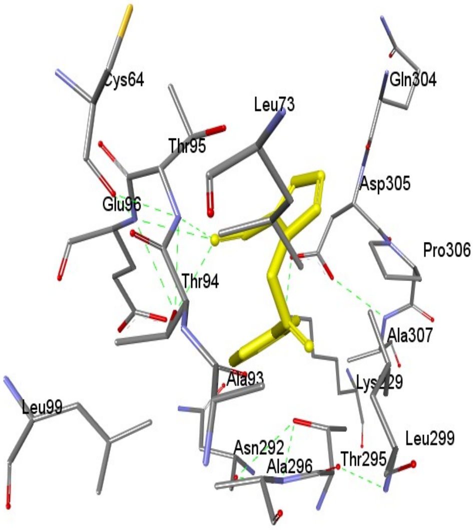

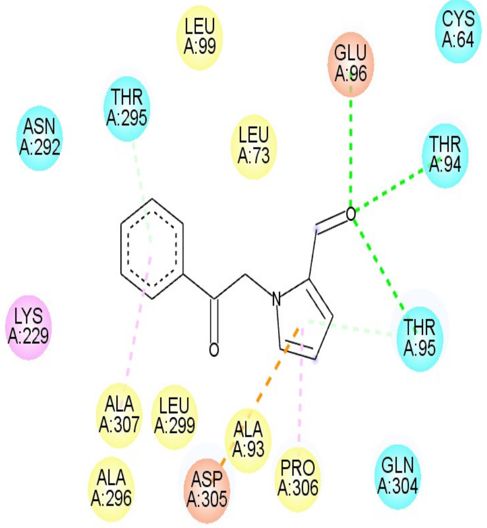

C. auris-**8g**

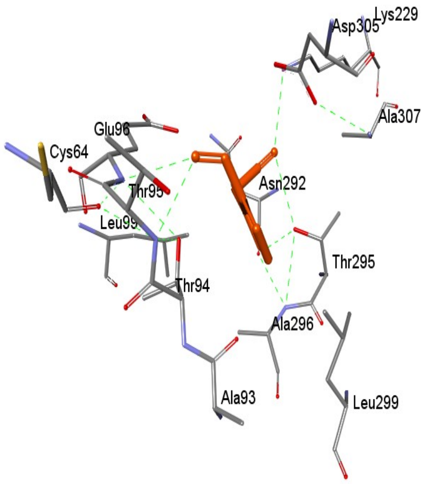

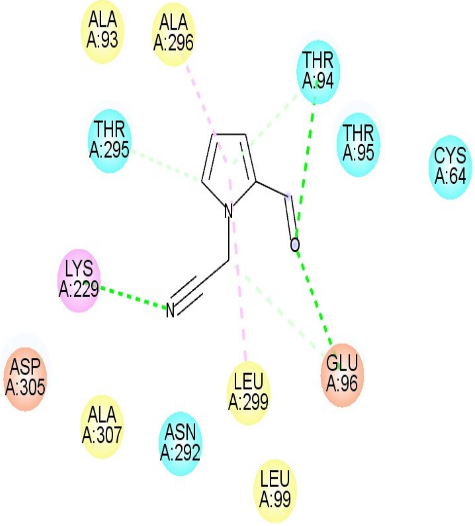

C. auris-**10a**

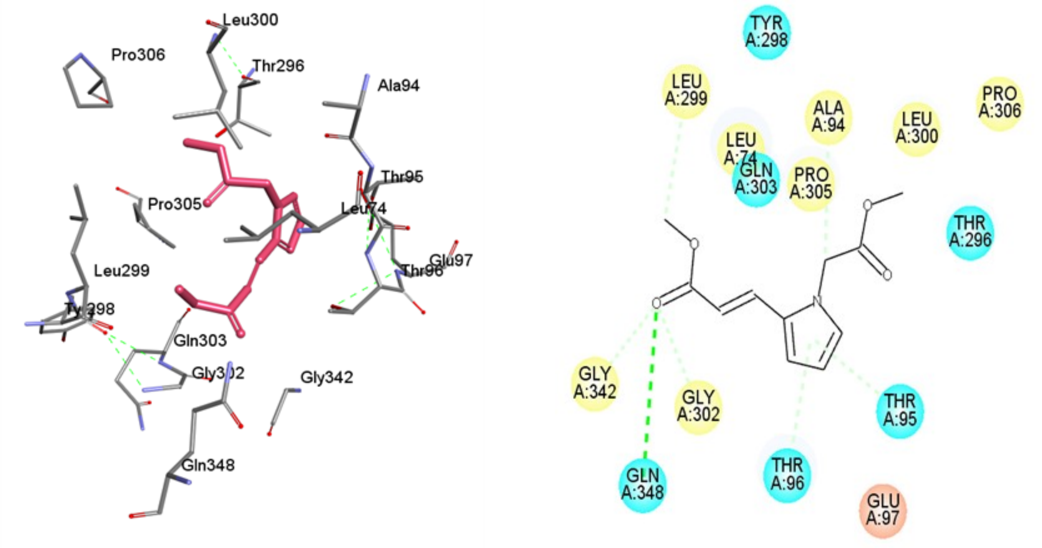

C. auris-**12a**

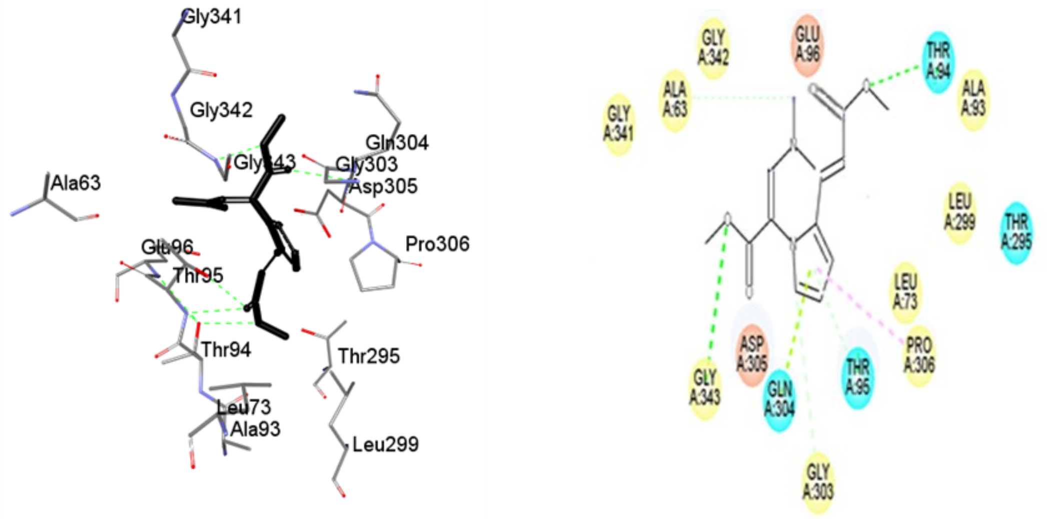

Figure S85. Representation of the interactions between the active site of HMGR of *C. auris* and simvastatin, atorvastatin, and enaminone-containing pyrroles **1a**, **2a**, and **2c**, pyrrolo[1,2-*a*]pyrazines **4b**, **4g**, and **4l**, indolizine **5a**, *N*-alkyl 2-formylpyrroles **8a**, **8c**, and **8g**, and pyrrole-based alkyl acrylates **10a** and **12a**. The 3D model shows the amino acid residues involved in ligand binding to the active site of the enzyme. In the 2D model, the following interactions are depicted with dotted lines: conventional hydrogen bond (dark green), carbon hydrogen (light green), π-sigma (purple), π-π T-shaped and π-π stacked (fuchsia), π-alkyl and alkyl (pink), π-anion (orange) and π-cation (yellow). The amino acids were

represented in pink circles (for basic amino acids), orange (for acid), cyan (for polars), and yellow (for non-polar).
